# A Bayesian two-stage framework for lineup-independent assessment of individual rebounding ability in the NBA

**DOI:** 10.1515/jqas-2023-0097

**Published:** 2024-12-25

**Authors:** Nicholas Kiriazis, Christian Genest, Alexandre Leblanc

**Affiliations:** Department of Mathematics and Statistics, 5620 McGill University, Montréal, Québec H3A 0B9, Canada; Department of Statistics, University of Manitoba, Winnipeg, Manitoba R3T 2N2, Canada

**Keywords:** basketball, Bayesian modeling, clustering, predictive inference, rebounding

## Abstract

In basketball, traditional methods of assessing individual rebounding ability are problematic because they depend on all players present on the court rather than just on the player of interest. Although there exist modeling approaches to correct for this dependence, they are generally unsuitable for events with binary outcomes. In this paper, a Bayesian two-stage model is proposed to predict both individual and team rebound allocation. This approach makes it possible to identify players who help their team win the fight for rebounds, regardless of their individual rebounding totals. Although similar in flavor to the popular Adjusted Plus-Minus (APM) framework, the proposed strategy is different in that it does not assume that individual contributions are linearly additive on the response scale. Furthermore, the regularization approach is improved through rebounding-specific heuristics. A simulation study is performed to show the effectiveness of the proposed model, and the parameters are estimated using data from the 2020–21 NBA season. Predictions are then made for rebounding in the 2021–22 season. This study confirms that relying exclusively on individual rebounding rates could lead to the mis-evaluation of players’ rebounding abilities.

## 1 Introduction

When assessing the viability of a potential lineup, basketball coaches generally need to know each player’s rebounding ability. The importance of understanding individual contributions to team rebounding is reflected in the historical evolution of score-keeping and analytics. Rebounds first made their way onto official score sheets in the 1950–51 season of the National Basketball Association (NBA), making them one of the earliest box-score statistics ever to be recorded; see Basketball-Reference.com (2023). At the time, “Rebounds per game” (RPG) was the gold standard for assessing individual rebounding ability. However, this measure is fundamentally flawed for player comparisons because raw rebound counts don’t consider the number of shots missed during a game. To correct for this deficiency, Oliver (2004) introduced the notions of offensive and defensive rebounding rate, which estimate the percentage of rebounds collected by a given player.

Despite the obvious improvement they provide, Oliver’s metrics still do not account for two important factors. First, given that a rebound allocated to one player is a missed rebound for another, rebounding rates do not only convey information about the individual’s ability. The fact that these rates also depend on the other players present on the court makes their use problematic for the assessment of individual players. Second, conventional wisdom suggests that it is possible for players to contribute to a team’s rebounding success without collecting rebounds themselves (players such as Steven Adams or Robin Lopez come to mind). Conversely, it is also commonly thought that, although some players collect many rebounds, those missed shots could have been collected by their teammates, regardless of their involvement. If these assumptions are accurate, then player assessment based purely on collected rebounds could be misleading in some cases.

The idea that true impact cannot be directly measured by counting stats is certainly not new: for example, using previously available tracking data, Franks et al. (2015) showed that some defenders excel by reducing their opponents’ field goal percentage, while others achieve a similar effect by reducing the frequency of opponents’ shots. Therefore, their defensive effectiveness cannot be captured by traditional box-scores. This is in fact the core idea behind the Adjusted Plus-Minus (APM) model of Rosenbaum (2004), which ignores box-scores entirely, and relies exclusively on players being on the court. However, in its original form, the APM framework is not suitable for binary events like rebounding, because it assumes that team performance can be expressed as a linear combination of individual player contributions. Much like RPG, a direct implementation of APM to rebounding would ignore the number of missed shots.

The purpose of this paper is to propose a Bayesian two-stage model to conditionally predict both individual and team rebound allocation using publicly available data. This approach allows for a distinction between players who keep rebounds away from the opposing team and players who claim rebounds from their own team. Crucially, it also explains how rebounding should work in arbitrary lineups and, in particular, in yet-to-be-seen lineups. Although similar in flavor to the popular APM framework, the proposed strategy is different in that it does not assume that individual contributions are linearly additive on the response scale. Furthermore, the regularization approach is improved by using rebounding-specific heuristics.

The high-level ideas of the new approach are outlined in Section 2. The rebounding models and accompanying prior structure are formally introduced in Section 3. The models are validated in Section 4 by making out-of-sample predictions for rebounding in the 2021–22 NBA season, and some of its practical applications are discussed. Section 5 concludes with a brief discussion.

The paper is complemented by an Appendix which describes an approach to reducing the dimension of the parameter space. A 30-page Online Supplementary comprising six sections also provides lists of variables used and detailed player statistics based on the analysis of the NBA 2020–21 season.

## 2 Background

### 2.1 Previous work in rebounding

Given the practical importance of rebounding, there is existing literature attempting to better understand the factors influencing rebounding and its strategic implications.

To study team rebounding, Maheswaran et al. (2012) used a support vector machine (SVM), along with optical tracking data, to predict which team will get the rebound, and explore how these predictions change depending on the current height of the yet-to-be-rebounded miss. Similarly, Csátaljay et al. (2017) relied on manually annotated data to test whether missed shots are more likely to be rebounded on the opposite side of the shooter’s location. Another study by Wiens et al. (2013) used optical tracking data to quantify the trade-off between going for the offensive rebound and getting ready for the transition to defense.

There were also studies conducted to explore rebounding at the individual level. Maheswaran et al. (2014) used optical tracking data to decompose rebounding into more interpretable components in order to better understand how individuals are contributing to the rebounding battle. Another study conducted by Hojo et al. (2019) used logistic regression and an SVM to predict individual and team rebound allocation based on the locations of the 10 players on the court.

In spite of their obvious merits, these studies all rely either on optical tracking data, manually annotated features from experts, or possibly both. They would thus be hard to implement with current NBA data. Indeed, tracking data are no longer publicly available, and manual annotation is entirely impractical. In contrast, the models to be considered here depend exclusively on publicly available NBA data.

### 2.2 Brief review of APM and RAPM

The basic principle of the original APM due to Rosenbaum (2004) is straightforward: good players, regardless of their box-score stats, will help their team outscore their opponents when they are on the court. Although simple in theory, this is complicated in practice because not all players enjoy the same quality of teammates on the court, nor do they face the same level of competition or have the same quality of substitutes.

To adjust for this, consider a matrix *X* with one column for each player in the league and an intercept column, and where every row is a portion of a game where no substitutions took place. On a given row, the column is set to 1 if the corresponding player is on their home court, −1 if the player is on an away court, and 0 if the player is not on the court. In the original implementation, the response vector *Y* is the net rating of the home team, which is linked to *X* via weighted linear regression with a diagonal weight matrix *W* whose entries are equal to the number of possessions played during each substitution-less stint. The vector *β* of the players’ individual contributions to the net rating (and the contribution of home court advantage) is then estimated by $\hat{\beta }={\left(X^{⊤}W^{-1}X\right)}^{-1}X^{⊤}Y$ .

There are three main drawbacks to the original APM implementation. First, because of the multicollinearity in *X*, the standard errors of the estimated regression coefficients are so large that the model can be practically unusable on out-of-sample data. Second, because of the design of the predictor matrix, there is no distinction between a player’s separate contributions to offense and defense, only the net difference between the two. Third, it can only be used for responses with infinite support, because it assumes a linear relationship between the individual level and the team level.

Sill (2010) built on the APM framework by using ridge regression. The design, response, and weight matrices are identical, but a hyperparameter *λ* is introduced. The estimates for individual contributions to net rating are then given by $\hat{\beta }={\left(X^{⊤}W^{-1}X+\lambda I\right)}^{-1}X^{⊤}Y$ , where *λ* is chosen to minimize the root-mean squared error (RMSE) in out-of-sample games and *I* denotes the identity matrix. By instilling a Gaussian prior on the parameters, the standard errors of the estimated parameters are more reasonably sized. This regularized APM, or RAPM for short, corrects for the first problem, but still suffers from the last two issues.

Despite these drawbacks, the APM approach has several desirable properties that are worth preserving. First, the main appeal of the APM framework is that it is designed to allow for prediction in unseen lineups. This is obviously very useful in practice, e.g., when negotiating contract extensions or determining who would be a desirable trade acquisition. Second, the APM approach allows to model the interaction between individual players and team response variables, without assuming that the players are making a measurable, direct contribution. Third, the APM approach accounts for the strength of teammates and opponents.

### 2.3 Decomposition of rebounding rate

The model proposed here is based on a decomposition of individual rebounding rates. Let A denote an arbitrary player and consider the probability that A collects a rebound conditional on there being a missed shot. One can then write

(1) $$\begin{array}{ll} \mathrm{Pr}\left(\text{A collects rebound}\right) & =\mathrm{Pr}\left(\text{A collects rebound}\cap \text{A’s team collects rebound}\right)\\ & =\underset{\gamma -level}{\underset{︸}{\mathrm{Pr}\left(\text{A collects rebound}|\text{A’s team collects rebound}\right)}}\times \underset{\beta -level}{\underset{︸}{\mathrm{Pr}\left(\text{A’s team collects rebound}\right)}}. \end{array}$$

Practically speaking, the above factorization implies that individual rebounding rates are a function of two separate abilities: contributions at the *β*-level (called *β*-ability), which involves securing rebounds at the team level, and contributions at the *γ*-level (called *γ*-ability), which involves personally securing rebounds that already belong to your team. There are then four variables of interest for each player, i.e., two offensive measures and two defensive measures.

While *β*- and *γ*-ability likely depend on each other, they differ, as shown by the following scenarios:

1) Player A boxes out Player B, allowing for a teammate to collect the rebound.
2) Player A boxes out Player B, and collects the rebound themselves.
3) Player A leaves Player B to put themselves in better position to collect the rebound, but also increasing the chance that Player B collects the rebound.

In Scenario 1, Player A arguably demonstrates positive *β*-ability and negative *γ*-ability while in Scenario 2, Player A demonstrates positive *β*-ability and positive *γ*-ability. In Scenario 3, Player A demonstrates negative *β*-ability and positive *γ*-ability. Note that this is not an exhaustive list of rebounding strategies; it just serves as an illustration of how different parameterizations can behave. Obviously, other tactical manifestations of a given parameterization are possible. For example, a situation in which a strong rebounder would vacate the paint to draw out their matchup to make rebounding easier for their teammates would be statistically equivalent to Scenario 1 (i.e., positive *β*-ability and negative *γ*-ability).

There are multiple benefits to decomposing rebounding in this way. First, from a practical viewpoint, it is reasonable to think that as long as the team collects the rebound, and hence controls the number of scoring chances, it is irrelevant which player actually secures the ball, and therefore, assessing *β*-ability is more important than assessing *γ*-ability. Second, in theory, this model is expressive enough to allow for players who are good rebounders but who don’t collect rebounds themselves, and players with high individual rebounding rates, but who do not actually help their team very much, which is in line with the conventional definitions of “winning players” and “stat-padders,” as given by Battier (2014). The formal models are given in Section 3.

### 2.4 Data

All data used in the present analysis are publicly available from the NBA. The data were collected using nba_api (Patel 2023), an API Client for www.nba.com. Although the data were drawn from a multitude of API endpoints, they can be categorized into two distinct types of data: box-score data and play-by-play data. Data from the 2020–21 NBA season were used to estimate the models, and data from the 2021–22 NBA season were used to evaluate model performance and validate the approach.

#### 2.4.1 Play-by-play data

The play-by-play dataset is simple: for every line of the play-by-play log of a given game, the substitution times of players were examined to determine who was on the court for each event. For every missed shot, distinguishing labels were given to players who were on offense, and those who were on defense.

One crucial point to note is the following: if the ball goes out of bounds after a missed shot, the team that gains possession is allocated a team rebound. Despite these not being credited to an individual player, they are often generated by players wrestling for position, trying to get their team the out-of-bounds call.

#### 2.4.2 Box-score data

The NBA has two methods for organizing game-level data: data can either be indexed by date or by game ID. Furthermore, most datasets are exclusively stored using one of the indexing schemes, meaning that one cannot directly combine all measurements into a single observation. This indexing hurdle was overcome by creating a correspondence table between game IDs and dates, allowing for a much richer dataset. Each row in the dataset (a player/game pair) is referred to as a performance. A full list of the retained variables is given in Part A of the Online Supplementary. Note that there were two types of measurements in the dataset:

a) Count variables such as steals, 3-point (3PT) shots attempted, etc.
b) Game summary variables such as average offensive speed, average defensive rebound distance, etc.

### 2.5 Parameter reduction

**Figure 1:**
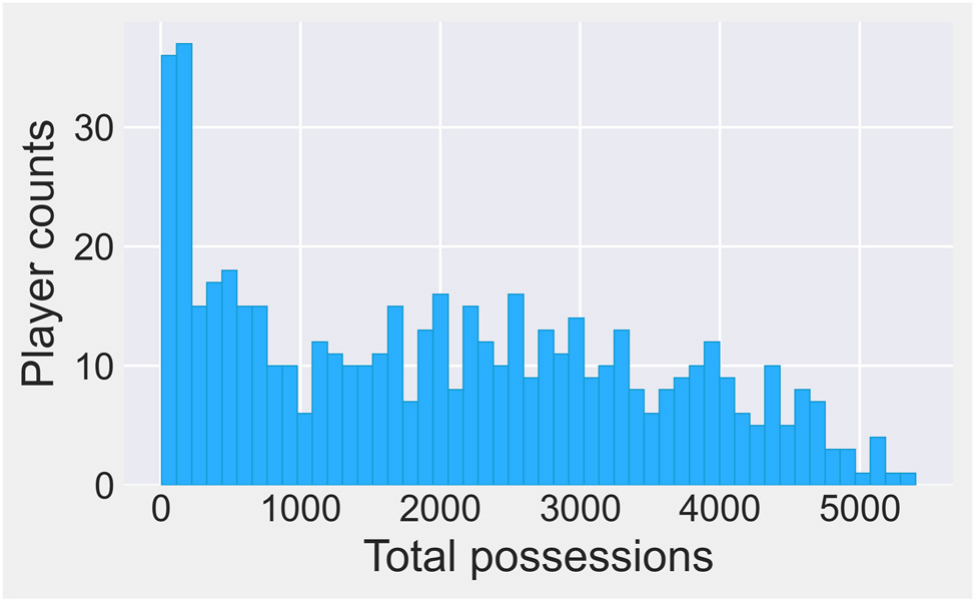
Distribution of possessions played by all 540 players in the 2020–21 NBA season.

Over the course of the 2020–21 NBA season, 540 different players appeared in at least one game. A histogram of the distribution of possessions played during the season is given in Figure 1. As can be surmised from the graph, many of these players had very limited opportunities. Indeed, many of them were merely an injury replacement, were played exclusively to rest starters once a game has been decided, or were simply given a trial run before being cut from the team. These will be referred to as “unusable players.”

Formally, a player is called (statistically) “unusable” if they appeared in fewer than 1,000 possessions. This cutoff, which results in 366 usable players, is subjective but corresponds to a natural break in the playing time histogram; see Figure 1. If a player had exactly 1,000 possessions, they would typically be the 13th player in the rotation on an average team. Given that most teams play 8–10 players, it is difficult to argue that a 1,000-possession player would have a very significant role. In effect, there is only a handful of players who are just below the 1,000-possession mark; most of them appear in under 200 possessions.

From a statistical perspective, unusable players are problematic because of the large number of parameters associated with them. Yet given the paucity of data about such players, it is clear at the outset that it would be impossible to obtain any statistically significant results for them. One can expect that treating them individually can lead to over-fitting and poor out-of-sample performance. Furthermore, a sizable portion of unusable players will never make another NBA appearance, implying that there is no point in estimating their rebounding ability.

To circumvent this issue, Rosenbaum (2004) chose to represent all unusable players by a single player, referred to as the replacement player. In the context of rebounding, however, this would implicitly assume that all these players should be considered as equivalent rebounders. This is almost certainly not the case: a 7-foot rim-running big man is almost certainly a different rebounder than a 5′10″ scoring guard, despite there potentially not being sufficient data to significantly test for this difference.

Therefore, a middle ground approach is used here: instead of representing all unusable players by the same replacement player, players with similar play styles are grouped, and the players in each group are represented by a group-specific replacement player. The rationale is that players with similar latent rebounding abilities are more likely to be grouped together, and therefore, that lineups with replacement players will behave similarly to their raw counterparts, which would lead to more accurate parameter estimates for non-replacement players. Although one could group players by their listed position, it is known that the latter are subjective and often inaccurate, especially for players with limited playing time. For this reason, box score data are used instead to measure similarity, as detailed in the Appendix.

## 3 Rebounding models

As shown in Eq. (1), a choice was made to model individual rebound allocation (*γ*-level) and team rebound allocation (*β*-level) separately. At the *γ*-level, modeling proceeds by treating each rebound allocation as a multinomial random variable with six possible outcomes: one outcome for each of the players, and the sixth for the case where the ball is sent out of bounds and a team rebound is allocated. At the *β*-level, each missed shot is treated as a trial, a defensive rebound as a success, and an offensive rebound as a failure. This makes it possible to handle simultaneously offensive and defensive *β*-ability, which makes sense given that they are in direct competition. As the outcomes are binary, Bayesian logistic regression is used as a modeling tool at the *β*-level. Also, given that individual rebounding outcomes are categorical, a modified form of Bayesian multinomial regression is used at the *γ*-level.

### 3.1 Gamma-level model

Recall that in traditional multinomial regression, some baseline category, say *k*, is chosen and a linear relationship is used to model the log-odds ratio between each non-baseline category and the baseline. Although multinomial regression seems like a natural way to model individual (conditional) rebounding probabilities, the implementation is not trivial: for every missed shot, the rebound must be either allocated to the team (when the ball goes out of bounds after the missed shot) or to one of the five players on the court. In other words, although one can treat each rebounding event as a multinomial random variable, the response categories differ for each trial, meaning that traditional multinomial regression, where each outcome can belong to any of the same possible categories, is not suitable.

To remedy this, it is assumed here that each player has some latent rebound collecting ability both on offense and defense, respectively denoted by $\gamma_{i}^{O}$ and $\gamma_{i}^{D}$ for Player *i*. It is further assumed that these abilities are continuous and that they are constant across all lineups. Furthermore, although players can differ between lineups, every lineup can potentially produce a team rebound, meaning that there is at least one common category for each of the multinomial random variables. Therefore, if one models the log-odds ratio between a specific individual collecting the rebound and there being a team rebound, the resulting framework allows for mixing and matching of lineups that have never been seen before.

Specifically, assume some lineup *L*, consisting of players 1–5, collects a defensive rebound (the same procedure holds for offensive rebounds). Assume that the conditional probability of Player *i* ∈ {1, …, 5} collecting that rebound is given by

$$p_{L,i}=\frac{{\text{e}}^{\gamma_{i}^{D}}}{1+{\text{e}}^{\gamma_{1}^{D}}+\cdots +{\text{e}}^{\gamma_{5}^{D}}},$$

and the probability of there being a team rebound is given by *p*_*L*,*T*_ = 1 − *p*_*L*,1_ − *p*_*L*,2_ − *p*_*L*,3_ − *p*_*L*,4_ − *p*_*L*,5_. Note that the probability of a team rebound therefore depends on the players on the court.

Because the response categories differ between responses, the likelihood is different from that of traditional multinomial regression. Let ***x***_1_, …, ***x***_*N*_ denote the 5-dimensional vectors of multinomial responses, where *N* denotes the total number of observed lineups. Let *n*_*i*_ denote the number of trials for the *i*th lineup (i.e., the number of rebounds needing to be allocated), and let *x*_*i*,*j*_ denote the number of rebounds collected by the *j*th player in this *i*th multinomial variable. The likelihood *L*(***γ***^***D***^; ***x***) of the above model, with the multinomial coefficient omitted for the sake of readability, is then proportional to

$$\begin{array}{ll} & \;\overset{N}{\underset{L=1}{\prod }}{\left(p_{L,1}\right)}^{x_{L,1}}\times {\left(p_{L,2}\right)}^{x_{L,2}}\times {\left(p_{L,3}\right)}^{x_{L,3}}\times {\left(p_{L,4}\right)}^{x_{L,4}}\\ & \;\times {\left(p_{L,5}\right)}^{x_{L,5}}\times {\left(p_{L,T}\right)}^{n_{L}-x_{L,1}-x_{L,2}-x_{L,3}-x_{L,4}-x_{L,5}} \end{array}$$

$$=\overset{N}{\underset{L=1}{\prod }}\frac{\exp\left(\gamma_{1}^{D}x_{L,1}+\gamma_{2}^{D}x_{L,2}+\gamma_{3}^{D}x_{L,3}+\gamma_{4}^{D}x_{L,4}+\gamma_{5}^{D}x_{L,5}\right)}{{\left(1+{\text{e}}^{\gamma_{1}^{D}}+\cdots +{\text{e}}^{\gamma_{5}^{D}}\right)}^{n_{L}}}.$$

Note that the same player can appear across multiple lineups with a different index, meaning that technically, the *γ*’s should also depend on the lineup, and that *γ*’s with different indices can actually be associated with the same underlying player. It is simple to show that the log-likelihood is proportional to

$$\begin{array}{ll} & \overset{N}{\underset{L=1}{\sum }}\left\{\gamma_{1}^{D}x_{L,1}+\gamma_{2}^{D}x_{L,2}+\gamma_{3}^{D}x_{L,3}+\gamma_{4}^{D}x_{L,4}+\gamma_{5}^{D}x_{L,5}\right.\\ & \;\left.-n_{L}\ln\left(1+{\text{e}}^{\gamma_{1}^{D}}+{\text{e}}^{\gamma_{2}^{D}}+{\text{e}}^{\gamma_{3}^{D}}+{\text{e}}^{\gamma_{4}^{D}}+{\text{e}}^{\gamma_{5}^{D}}\right)\right\}. \end{array}$$

Directly maximizing the log-likelihood in this instance is less problematic than most APM-like approaches: instead of players confounding each other’s contributions to the response, one actually has more “complete” information, because one knows exactly which player collected the rebound. This means that although player appearances are correlated, they don’t lead to an explosion in the variance of parameter estimates. That being said, lineup mixture still obviously eases parameter estimation.

However, because this is not a traditional implementation of multinomial regression, standard techniques for maximizing the likelihood are unsuitable. As an alternative, it is proposed here to estimate the parameters using Markov Chain Monte Carlo (MCMC) by instilling (improper) uniform priors over the real line. To this end, note that the posterior distribution is proportional to the product of the prior *π* and the likelihood L , i.e., for all possible values of ***γ***, ***x***,

π(γ∣x)∝π(γ)×L(γ∣x).

Therefore, by using the posterior mean to estimate the parameters, one is effectively using a weighted average over the parameter space, where the weight is proportional to the likelihood at the given point.

### 3.2 Beta-level model

Logistic regression is a natural modeling approach for the team rebounding problem: it allows one to assume that team rebounding ability is additive on the log odds scale. This preserves the predictive versatility of the original APM framework as one can just add up the individual contributions to team rebounding. Logistic regression also allows one to capture “diminishing returns” in team rebounding rates: one would expect that adding a strong *β*-level rebounder to a strong rebounding lineup has a less pronounced impact on the team’s rebounding rate than if one added that same player to a weak rebounding lineup. For the sake of tractability, variables such as days of rest or home court advantage are not controlled for, but instead it is assumed that rebounding ability is constant from game to game.

Let *L* denote some arbitrary combination of five offensive players and five defensive players. Further let $\beta_{1}^{D}$ , …, $\beta_{5}^{D}$ denote the defensive *β*-abilities of the defensive players, and let $\beta_{1}^{O}$ , …, $\beta_{5}^{O}$ denote the offensive *β*-abilities of the offensive players. The most straightforward model for the probability of the defensive team collecting the rebound is given by

(2) $$q_{L}=\frac{{\text{e}}^{\beta_{1}^{D}+\cdots +\beta_{5}^{D}-\beta_{1}^{O}-\cdots -\beta_{5}^{O}}}{1+{\text{e}}^{\beta_{1}^{D}+\cdots +\beta_{5}^{D}-\beta_{1}^{O}-\cdots -\beta_{5}^{O}}}.$$

With this parameterization, the larger the value of a parameter, the larger the *β*-ability of the associated player, regardless of whether one is talking about offensive or defensive rebounding. Although appealing because of its simplicity and how it naturally opposes offense and defense, a traditional implementation of this model suffers two main drawbacks: multicollinearity and unidentifiability.

#### 3.2.1 Dealing with multicollinearity

When estimating the parameters of a Generalized Linear Model (GLM) by maximizing the likelihood, the distribution for the estimator is asymptotically Gaussian. More precisely, one has

$$\hat{\beta }\approx N\left[\beta ,{\left(X^{⊤}WX\right)}^{-1}\right],$$

where ***W*** is a diagonal matrix whose *i*th element is of the form $w_{i}={\left(\partial \mu_{i}/\partial \eta_{i}\right)}^{2}/var\left(y_{i}\right)$ , where *μ*_*i*_ is the *i*th mean response, *y*_*i*_ is the *i*th observation, and *η*_*i*_ = ln{*μ*_*i*_/(1 − *μ*_*i*_)}. See Agresti (2015) for details.

As is the case with linear regression, the variance of the components of $\hat{\beta }$ can become arbitrarily large if there is severe multicollinearity in the design matrix. One could replicate the approach of Sill (2010) by using ridge regression, but in the context of rebounding, there is reason to believe one could do better.

Although individual rebounding rates may be misleading when determining whether, e.g., Jonas Valanciunas is a better *β*-level offensive rebounder than Steven Adams, it is probably telling when the difference in individual rates is large, i.e., Jonas Valanciunas (individual OREB% of 13.4 %) is almost certainly helping his team’s offensive rebounding more than Duncan Robinson (individual OREB% of 0.3 %). Although the modeling approach allows to distinguish between *γ*-ability and *β*-ability, it can be argued that instances where there is a drastic difference in these two latent variables are probably rare, and that one should only assume that they exist when the evidence is overwhelming.

With this in mind, instead of instilling an identical prior across all players, it is proposed here that individual rebounding rate be reflected in the choice of prior. Although this dictates the stochastic ordering, because rebounding ability is modeled on the log odds scale, it is not obvious what exactly the priors should be. To better understand measuring rebounding on the log-odds scale, model identifiability is discussed first.

#### 3.2.2 Dealing with unidentifiability

Assume that the model described above is an accurate representation of the true rebounding process, and let $\beta_{*}^{D}$ and $\beta_{*}^{O}$ denote the underlying parameter values. In its proposed form, the model is not identifiable: adding the same constant *c* to every component of $\beta_{*}^{D}$ and to every component of $\beta_{*}^{O}$ does not alter the likelihood. This is not a problem, however, considering that the main goal of the model is to rank the rebounders and predict their performance in unseen lineups. For this reason, the actual parameter values themselves are not important: if the same constant is added to every component, these rankings and model predictions are unchanged.

To illustrate a more serious issue, consider a hypothetical case where there would be only two defensive players, with defensive *β*-abilities denoted by $\beta_{1}^{D}$ and $\beta_{2}^{D}$ , and two offensive players, with offensive *β*-abilities denoted by $\beta_{1}^{O}$ and $\beta_{2}^{O}$ . Also assume that there is only one player per team, and that there is limited lineup mixture, so that one has only observed $\beta_{1}^{D}$ against $\beta_{1}^{O}$ , and $\beta_{2}^{D}$ against $\beta_{2}^{O}$ , viz.

$$\ln\left(\frac{q_{1}}{1-q_{1}}\right)=\beta_{1}^{D}-\beta_{1}^{O},\;\ln\left(\frac{q_{2}}{1-q_{2}}\right)=\beta_{2}^{D}-\beta_{2}^{O}.$$

In this simple case, one could add some constant *c*_1_ to $\beta_{1}^{D}$ and to $\beta_{1}^{O}$ , and add some constant *c*_2_ to $\beta_{2}^{D}$ and to $\beta_{2}^{O}$ . One could astutely pick the constants such that either defensive player can be chosen to be the best rebounder, or either offensive player can be chosen to be the best rebounder. Any such model solution is much more problematic. However, if all possible lineup combinations are used, all model parameterizations will preserve the ordering of the parameter magnitudes. For a proof of this result, refer to the first author’s Master’s thesis (Kiriazis 2023). Of course in practice, not all possible lineups are observed, which means that the estimated solution is not guaranteed to preserve the true underlying parameter ranking.

By looking at the simple two-player example, it is obvious that any amount of shift in the defensive parameters must induce an equal (in the aggregate) shift in the offensive parameters, and hence, given an incomplete system, one can find a valid parameterization that makes any player appear as the best rebounder. It seems heuristically reasonable to believe that the more lineup mixture one has, the less likely it is for there to be re-ordering of the parameters. This is why grouping rarely observed players is so important: it makes it highly unlikely for there to be subgroups (containing both offensive and defensive players) who have played exclusively among themselves, and for these “subsystems” of independent equations to arise. Furthermore, notice that if one were to restrict the parameter space of the model, one would limit how extreme any potential re-ordering of the parameters can be.

Although there is no mathematically rigorous way to restrict the parameter space, one can consider the following thought experiment. Suppose that one could clone the best defensive rebounder, with ability $\beta_{\max}^{D}$ , and play them against an average offensive rebounding lineup, whose aggregated offensive rebounding ability is given by *c*. It seems reasonable to assume that, although one doesn’t know the exact defensive rebounding rate for this lineup, it is certainly not larger than 90 %. Suppose one could also clone the worst defensive rebounder, with ability $\beta_{\min}^{D}$ , and play them against an average offensive rebounding lineup. Again, one can’t say for sure what the true defensive rebounding rate is in this case, but it seems heuristically reasonable that the percentage must be larger than 50 %.

These bounds seem reasonable if not abundantly cautious: of lineups having played at least 200 possessions, the lowest empirical defensive rebounding rate was 64 %, and the greatest empirical defensive rebounding rate was 84 %, according to Falk (2021). Referring to Eq. (2), one can express these restrictions as follows:

$$\frac{{\text{e}}^{5\beta_{\max}^{D}-c}}{1+{\text{e}}^{5\beta_{\max}^{D}-c}}\leq 0.9,\;\frac{{\text{e}}^{5\beta_{\min}^{D}-c}}{1+{\text{e}}^{5\beta_{\min}^{D}-c}}\geq 0.5.$$

The interest is in finding a support for the defensive parameters that would allow for predictions as extreme as those laid out above, but that is as “narrow” as possible, to limit the potential for re-ranking of the parameters. This can be formulated as follows in terms of an optimization problem, namely minimize $\beta_{\max}^{D}-\beta_{\min}^{D}$ under the constraints

$$\frac{{\text{e}}^{5\beta_{\max}^{D}}}{1+{\text{e}}^{5\beta_{\max}^{D}}}-\frac{{\text{e}}^{5\beta_{\min}^{D}}}{1+{\text{e}}^{5\beta_{\min}^{D}}}\geq 0.4\;\text{and}\;\beta_{\max}^{D}\geq \beta_{\min}^{D}.$$

Note that one can just add *c*/5 to each parameter, re-parameterize, and solve this slightly simpler but equivalent form of the problem, given that $\left(\beta_{\max}^{D}-c/5\right)-\left(\beta_{\min}^{D}-c/5\right)$ has the same solution, and the interest only lies in the difference between the two parameters. Given that the constraint is simply the difference of two independent sigmoids, the gradient can easily be computed, and the optimal solution is readily found using the Lagrangian multiplier. One can, therefore, compute that $\beta_{\max}^{D}-\beta_{\min}^{D}\leq 0.340$ .

If the same thought experiment is repeated but for offensive rebounding parameters (and instead allowing for there to be a difference of 35 % instead of 40 %, given that offensive rebounding rates tend to exhibit less variability than defensive rebounding rates), one finds $\beta_{\max}^{O}-\beta_{\min}^{O}\leq 0.292$ . Note that this range is very similar to that of empirical individual rebounding rates.

Although the assumptions imply that there is some meaningful parameterization of the model in which the offensive parameters are “close” to each other, and defensive parameters are “close” to each other, nothing has been said about the distance between the collection of offensive parameters and the collection of defensive parameters. However, note that the model can be re-parameterized as follows:

$$\begin{array}{ll} q_{L} & =\frac{{\text{e}}^{\beta_{1}^{D}+\cdots +\beta_{5}^{D}-\beta_{1}^{O}-\cdots -\beta_{5}^{O}}}{1+{\text{e}}^{\beta_{1}^{D}+\cdots +\beta_{5}^{D}-\beta_{1}^{O}-\cdots -\beta_{5}^{O}}}=\frac{\exp\left\{\beta_{1}^{D}+\alpha_{D}+\cdots +\beta_{5}^{D}+\alpha_{D}-\left(\beta_{1}^{O}+\alpha_{O}\right)-\cdots -\left(\beta_{5}^{O}+\alpha_{O}\right)-5\alpha_{D}+5\alpha_{O}\right\}}{1+\exp\left\{\beta_{1}^{D}+\alpha_{D}+\cdots +\beta_{5}^{D}+\alpha_{D}-\left(\beta_{1}^{O}+\alpha_{O}\right)-\cdots -\left(\beta_{5}^{O}+\alpha_{O}\right)-5\alpha_{D}+5\alpha_{O}\right\}}\\ & =\frac{\exp\left(\beta_{1}^{*D}+\cdots +\beta_{5}^{*D}-\beta_{1}^{*O}-\cdots -\beta_{5}^{*O}+\alpha \right)}{1+\exp\left(\beta_{1}^{*D}+\cdots +\beta_{5}^{*D}-\beta_{1}^{*O}-\cdots -\beta_{5}^{*O}+\alpha \right)}, \end{array}$$

where the new parameterization simply shifts all the defensive parameters by some fixed amount, and all the offensive parameters by some other fixed amount.

As explained above, solutions to these two alternative parameterizations would be equivalent in practice, given that they preserve the ordering of the parameters and yield identical predictions. Note that as long as the heuristics about the maximal difference between parameters is correct, and as long as *α* is free, one can restrict the rebounding parameters to their respective ranges, and still be able to obtain useful estimates.

#### 3.2.3 A Bayesian solution

In summary, to ensure that a workable solution can be found, the following assumptions were made about the underlying model parameters:

1) Extreme differences in individual rebounding rates likely suggest a difference in *β*-ability.
2) The defensive model parameters are probably close to each other and the offensive model parameters are probably close to each other; moreover, they probably exhibit a similar spread to those of individual offensive and defensive rebounding rates.
3) If the offensive and defensive parameters are both restricted to some subspace, one needs an unrestricted parameter to allow for each group to be adequately far apart.

Thus far, a formal meaning has not been given to the word “restricted,” and the above discussion has relied almost exclusively on heuristics, but a Bayesian model offers a natural, mathematically rigorous framework to implement parameter restrictions. Indeed, in a Bayesian model one can effectively “restrict” the parameter space by instilling some informative prior distribution on the parameters and, conversely, leave parameters unrestricted by instilling an uninformative prior.

Accordingly, the following hierarchical Bayesian framework is proposed. For the *i*th defensive player and *j*th offensive player, assume

$$\begin{array}{ll} & \beta_{i}^{D}|DREB{\%}_{i},\sigma ∼N\left(DREB{\%}_{i},\sigma^{2}\right),\\ & \beta_{j}^{O}|OREB{\%}_{j},\sigma ∼N\left(OREB{\%}_{j},\sigma^{2}\right), \end{array}$$

and letting *Y*_*L*,*k*_ denote whether the *k*th missed shot in the lineup *L* is a defensive rebound (a success) or an offensive rebound (a failure), assume

$$Y_{L,k}|\left(\beta_{i_{1}}^{D},\ldots ,\beta_{i_{5}}^{D},\beta_{j_{1}}^{O},\ldots ,\beta_{j_{5}}^{O},\alpha \right)∼\text{Bernoulli}\left(q_{L}\right),$$

where

$$q_{L}=\frac{{\text{e}}^{\beta_{i_{1}}^{D}+\cdots +\beta_{i_{5}}^{D}-\beta_{j_{1}}^{O}-\cdots -\beta_{j_{5}}^{O}+\alpha }}{1+{\text{e}}^{\beta_{i_{1}}^{D}+\cdots +\beta_{i_{5}}^{D}-\beta_{j_{1}}^{O}-\cdots -\beta_{j_{5}}^{O}+\alpha }},$$

and *α* has a flat prior on the real line. Note that in the case of replacement players, all the rebounds and rebounding opportunities of the grouped players were aggregated to create the rebounding rate.

To justify the choice of mean for each of the parameters, note that the largest individual offensive rebounding rate was 15.5 % (Clint Capela) and the smallest was 0.3 % (Duncan Robinson). The largest individual defensive rebounding rate was 33.6 % (Andre Drummond) and the smallest was 4.7 % (Trey Burke). These spreads are slightly smaller than the ones implied by the above thought experiments.

Beyond the rough parameter restrictions suggested by the thought experiment, there is no obvious choice for the variance of the priors. Although Bayesian applications often rely on high variance and minimally informative priors, using a broad prior would be akin to just maximizing the likelihood given the very severe data limitations of the current context. Ideally, one would like to pick the maximal prior variance that allows for meaningful and useful parameter estimates, so as to maximize the impact of the data on parameter estimates and minimize the impact of the prior information. With this in mind, the prior variance is set equal to four times the variance of the observed individual rebounding rates, which yields a prior standard deviation of 0.1. There are a few reasons for this.

First, since it was hypothesized that the effect on the log odds can be modeled using a similar scale to that of the rebounding rates, it follows naturally that scaling up the variance of the rebounding rates is a sufficiently cautious approach.

Second, with this choice, the upper bound on a 95 % prior interval for the best *β*-level defensive rebounder is 0.532, and the lower bound on a 95 % confidence interval for the worst *β*-level defensive rebounder is −0.149. If the thought experiment is broadly reasonable, then there is thus ample “room” to capture the variability of different players. Similarly, the likely range of the offensive parameters lies between −0.193 and 0.351.

Third, from a practical standpoint, the chosen variance seems to be sufficiently cautious when passing judgment about relative player quality. For example, the priors suggest that *a priori*, there is a 93 % chance that Clint Capela is a better defensive rebounder than Trey Burke, which seems a bit too optimistic about Burke’s ability; the priors also suggest only a 59 % chance that Andre Drummond is better than Jonas Valanciunas, who had a defensive rebounding rate of 28.9 %. Although this doesn’t truly restrict the possible parameter values, it does achieve a similar effect, by making extreme values unlikely.

#### 3.2.4 A simulation study

Given that the proposed model relies heavily on heuristics about how to reduce the parameter space, and that in the case of non-identifiability, the posterior distributions are influenced by the choice of priors, it seems wise to see how well the proposed model can recover the parameters under realistic (albeit simplified) conditions. Before running a simulation study, define the following data structures:

1. **Players**: Each player has a known defensive rebounding attribute, *β*^*D*^, which is generated from a Normal distribution, and a known offensive rebounding attribute, *β*^*O*^, which is generated from a separate Normal distribution. The choice of parameters for these Normal distributions is discussed below.
2. **Teams**: Each team has eight players. A single lineup is created by sampling five players without replacement. This is done 30 times to create a list of 30 lineups that will be used when playing games. Each lineup is then assigned a weight by sampling from a symmetric Dirichlet distribution over the 30-dimensional simplex. The weight of the *i*th lineup of team A is denoted by $w_{i}^{A}$ .
3. **Games**: For each game, eight lineups are drawn from each of the two teams, with the probability of being drawn equal to each lineup weight. The weights of the drawn lineups are scaled to determine the proportion of playing time of each lineup, i.e., $L_{i}^{A}$ will play $w_{i}^{A}/\left(w_{i_{1}}^{A}+\cdots +w_{i_{8}}^{A}\right)$ of the game. Each game consists of 100 missed shots (50 per team) that need to be allocated to a player.
4. **Allocating rebounds**: Within a given lineup, the team is first allocated a rebound with probability
$$q=\frac{{\text{e}}^{\beta_{i_{1}}^{D}+\cdots +\beta_{i_{5}}^{D}-\beta_{j_{1}}^{O}-\cdots -\beta_{j_{5}}^{O}}}{1+{\text{e}}^{\beta_{i_{1}}^{D}+\cdots +\beta_{i_{5}}^{D}-\beta_{j_{1}}^{O}-\cdots -\beta_{j_{5}}^{O}}}.$$
  Then, based on which team collected the rebound, that rebound is randomly allocated to an individual player. The probability that Player *i* is assigned an individual defensive rebound is given by
$$p_{i}^{D}=\frac{{\text{e}}^{4\beta_{i}^{D}}}{{\text{e}}^{4\beta_{1}^{D}}+\cdots +{\text{e}}^{4\beta_{5}^{D}}},$$
  where the numerator is the sum across all other players appearing in the same lineup as Player *i*. Similarly, team offensive rebounds are conditionally allocated to individual *i* with probability
$$p_{i}^{O}=\frac{{\text{e}}^{7\beta_{i}^{O}}}{{\text{e}}^{7\beta_{1}^{O}}+\cdots +{\text{e}}^{7\beta_{5}^{O}}}.$$
  Effectively, to get the *γ*-level parameters, one is just scaling up the corresponding *β*-level parameters. The scaling values were chosen so that the simulated individual probabilities more closely resemble observed probabilities. Directly using the parameters does not allow for sufficient variability in the individual rebounding rates to match observed rates. Also note that it was not deemed worthwhile to simulate team rebounds because they do not affect the stochastic ordering of the priors.

Given these structures, the actual simulation algorithm is straightforward: every team plays each opponent thrice (meaning each team plays a total of 87 games instead of the usual 82). During each game, each team will miss 50 shots, and their opponents will miss 50 shots (teams on average miss 47 field goals per game, according to Basketball-Reference.com), each of which is then allocated to an individual player.

Obviously there is nowhere near as much complexity in this simulation as there is in actual NBA games. Therefore, briefly reviewed below are the facilitating simplifications and impeding simplifications.

Facilitating simplifications are simplifications which make parameter estimation easier than real-world conditions. The most notable of the simplifications is that not only are team rebounding ability and individual rebound collecting correlated, they are perfectly dependent. This was done mainly because it was unclear how to link the two latent variables without snooping through the data. Another significant simplification is that lineups were generated randomly, implying that there is probably less multicollinearity in the simulation than there is in the true league. Lastly, there are the practical simplifications, like having fewer players per team, players not getting injured, or there not being any team rebounds. It seems reasonable to believe that these simplifications have a minimal impact on parameter estimates.

Impeding simplifications are simplifications which make parameter estimation more difficult than real-world conditions. One key simplification is that there are no replacement-like players and no trades, which greatly decreases the amount of lineup mixture, and makes it more difficult to construct priors that are consistent with each other (since lineup mixture is key to preserving the ordering of rebounding ability). Also, given that the lineups and players are truly random, there are probably instances of very unrealistic lineup combinations, implying that the variability between lineups is much greater than in the true league.

The hope is that both the impeding and facilitating simplifications roughly cancel each other out, and make for broadly reasonable test conditions.

Parameters for the two Normal distributions used to generate the players were chosen based on the observed rebounding rates of five-man lineups having appeared in at least 100 offensive possessions and 100 defensive possessions during the 2020–21 NBA season. Note that instances with the same 10 players have too little game time for the probabilities to be meaningful.

To judge whether the Normal parameters were appropriate, 5,000 ten-man combinations were created by sampling five players from the defensive distribution and five players from the offensive distribution. The probabilities for these lineups were computed using the logistic function, and the simulated distribution was compared to the observed data.

The mean of the defensive and offensive distributions were chosen to be 0.22 and 0, respectively. One can achieve nearly identical lineup rebounding rates to the observed ones by setting the standard deviation to 0.1 for each of the two Normal distributions. However, the observed rates are empirical, can contain as few as 50 trials, and are marginalized across all opponents, which means that the variance in the observed lineup rebounding is likely larger than the variance of the true underlying probabilities that will be used in the simulation. With this in mind, a second simulation was also run, but with a standard deviation of 0.07.

The simulation was carried out for each choice of variance, as described above. The parameters were estimated in Stan, using the Hamiltonian Monte Carlo implementation of Carpenter et al. (2017). The pseudo-code for the estimation procedure is given in Algorithm 1. See Brooks et al. (2011) and Neal (2012) for a more detailed discussion about the Metropolis–Hastings algorithm and Hamiltonian dynamics. The Markov chains consisted of 1,000 warm-up iterations and 1,000 sampling iterations. To evaluate the convergence of the process, four separate chains were used and the values of $\hat{R}$ , as described by Vehtari et al. (2021), were calculated for each marginal distribution. In all instances, these were close to 1. This was especially important for the estimate $\hat{\alpha }$ of the intercept, given that the prior was improper.

To evaluate the accuracy of the model, however, one cannot simply compare the estimated parameters to the known ones. For, as mentioned earlier, one can shift any parameterization by a constant, and end up with an equivalent model. This is why the inclusion of an intercept term, when estimating the parameters, was crucial. To make comparisons possible, it was assumed that the shift across all parameters is constant, and the total shift for each lineup is aggregated into *α*.

**Algorithm 1.**

Markov Chain Monte Carlo for sampling from target distribution *p*.

**Table:** 

Initialize *x*_1_
**for** *i* in {1, …, *N*} **do**
Generate u∼U(0,1)
Generate proposal $x^{*}∼q\left(x^{*}|x_{i}\right)$
**if** $u<\frac{p\left(x^{*}\right)q\left(x_{i}|x^{*}\right)}{p\left(x_{i}\right)q\left(x^{*}|x_{i}\right)}$ **then**
*x*_*i*+1_ = x*
**else**
*x*_*i*+1_ = *x*_*i*_
**end if**
**end for**

For this reason, one can rewrite the linear predictor as

$$\begin{array}{ll} & \beta_{1}^{D}+\beta_{2}^{D}+\cdots +\beta_{5}^{D}-\beta_{1}^{O}-\beta_{2}^{O}-\cdots -\beta_{5}^{O}+\alpha \;\\ & \;=\left(\beta_{1}^{D}+\alpha /10\right)+\left(\beta_{2}^{D}+\alpha /10\right)+\cdots +\left(\beta_{5}^{D}+\alpha /10\right)\\ & \;\;-\left(\beta_{1}^{O}-\alpha /10\right)-\left(\beta_{2}^{O}-\alpha /10\right)-\cdots -\left(\beta_{5}^{O}-\alpha /10\right). \end{array}$$

**Figure 2:**
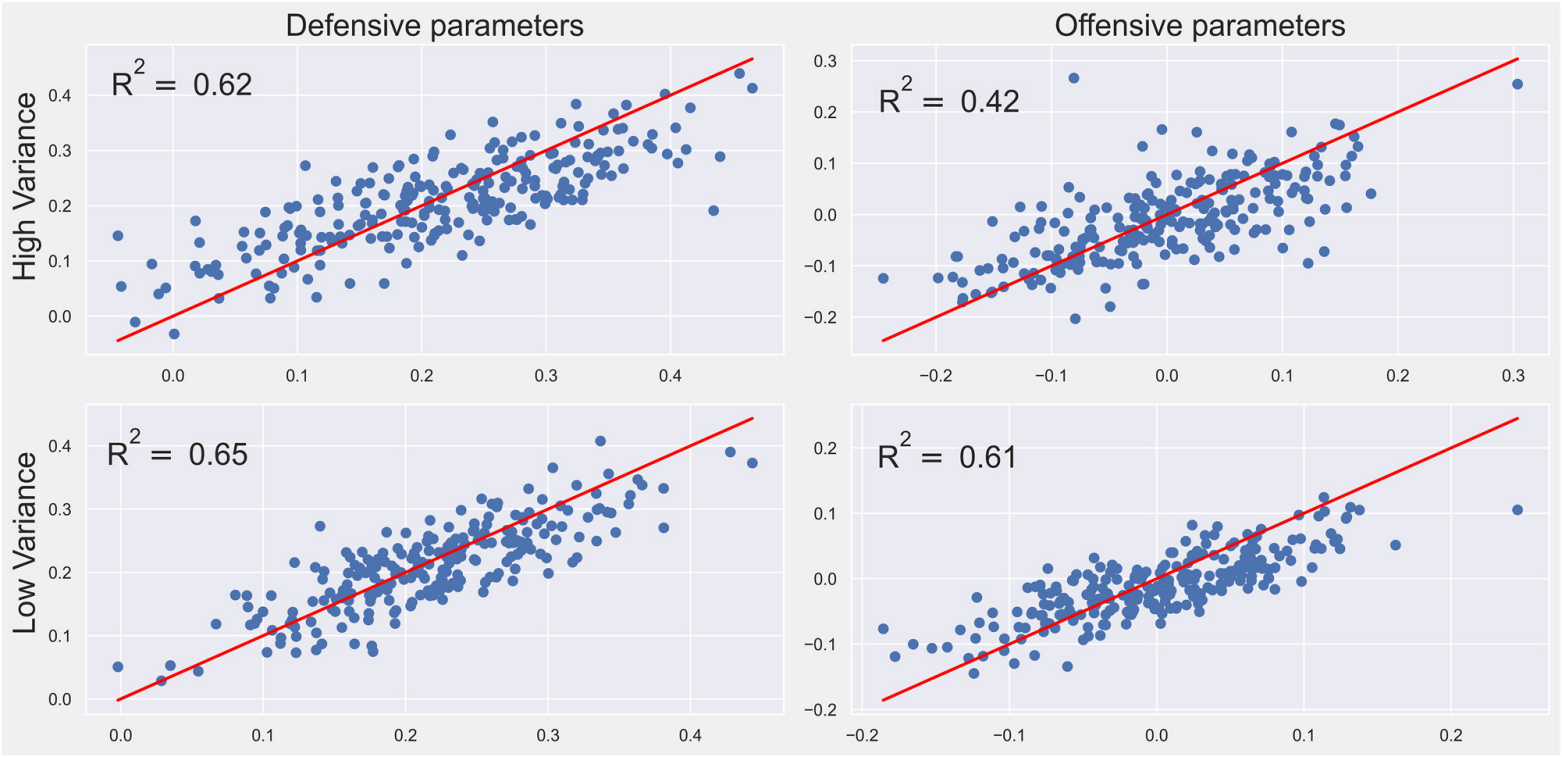
Scatter plot of shifted estimated parameters (*y*-axis) against their true values (*x*-axis). Note that because of the support of the prior distributions, it is more difficult to capture players with extreme *β*-level parameters.

Therefore, the shifted parameter estimates were compared with the parameter values used to create the simulated data. Figure 2 contains scatter plots of the shifted estimated parameters against their true values.

Although the method seems to work reasonably well in general, there are a few outliers, the most concerning/interesting of which is the one player found far above the cloud in the offensive high-variance graph. This could be an artefact of lineup construction: given that the lineups were completely random, if a weak rebounder just happened to have teammates and/or opposition that were even weaker, it would be nearly impossible to detect that they were weak. In practice, lineup strategy probably makes for more homogeneous lineups that the randomly generated ones. Removing this point alone increases the value of *R*^2^ from 0.42 to 0.48. Given the satisfactory behavior of the suggested estimation strategy, the proposed methodology could be applied with confidence to the NBA dataset. This is done in Section 4.

## 4 Results

### 4.1 Posterior distributions of the parameters

Before formally evaluating the model fit, it is relevant to explore the posterior distributions, to see if they seem heuristically reasonable. As in the simulation study, the parameters for both *β*-ability and *γ*-ability were estimated using the method of Carpenter et al. (2017), with Markov chains consisting of 1,000 warm-up iterations and 1,000 sampling iterations. Again, the convergence was evaluated by running four chains, and computing the value of $\hat{R}$ for all marginal distributions. All parameters, including the estimated intercept $\hat{\alpha }$ , yielded $\hat{R}\approx 1$ . The estimates for each player having appeared in at least 1,000 possessions can be found in Part B of the Online Supplementary.

#### 4.1.1 Beta-level

The average of the defensive posterior means is equal to 0.139, the average of the offensive posterior means is equal to 0.0406, and the posterior mean for the intercept is equal to 0.535. Thus if one were to play five average defensive rebounders against five average offensive rebounders, the predicted defensive rebounding rate of the team would be 73.63 %, which is nearly identical to the league-wide average defensive rebounding rate of 73.8 %. This feature is particularly interesting and helps further validate the approach.

**Figure 3:**
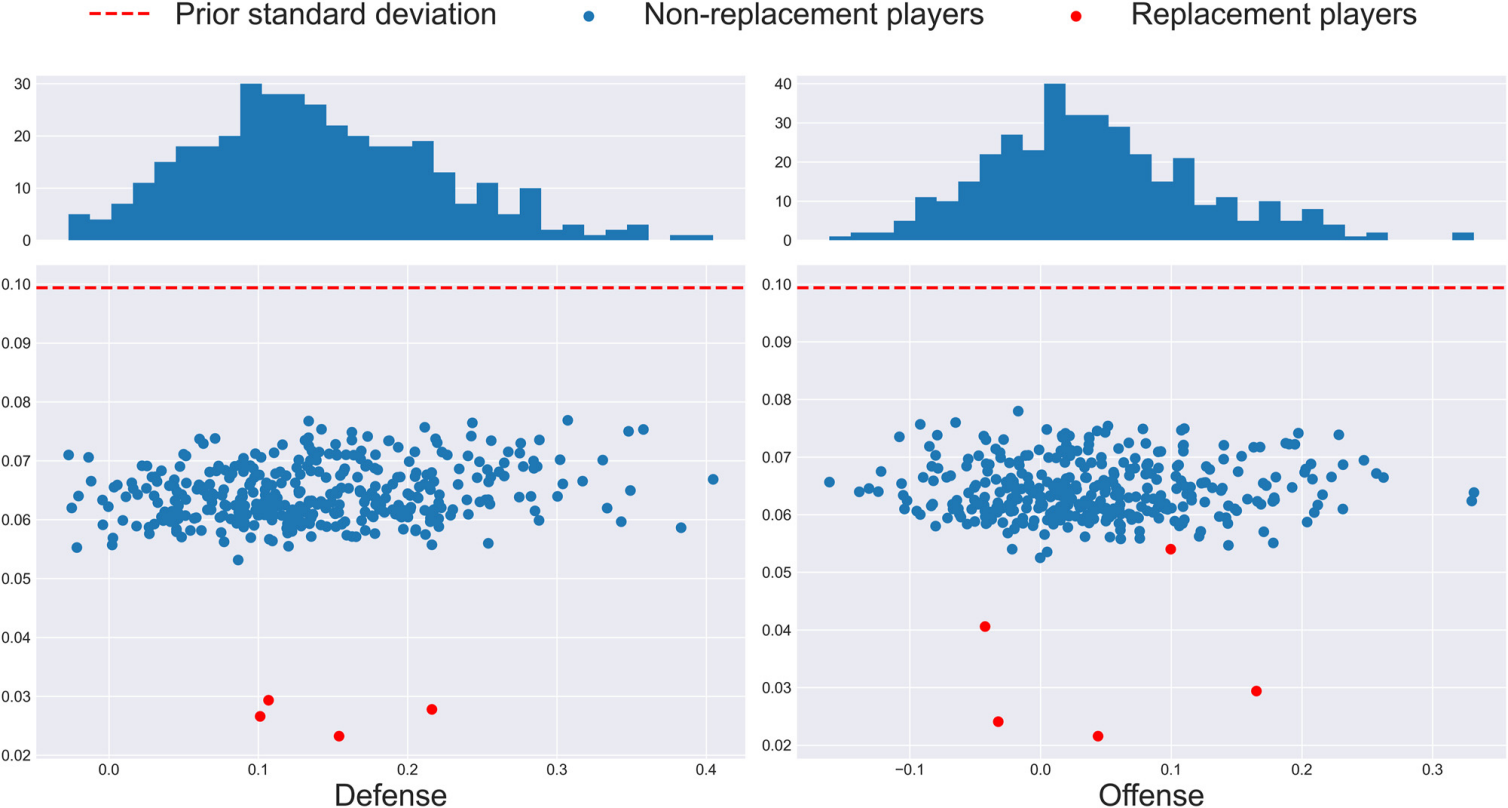
Standard deviation (*y*-axis) of each posterior distribution against the posterior mean (*x*-axis) of the *β*-level parameters. Given the unequal replacement player partition, the difference in replacement player posterior variance is to be expected.

As shown by Figure 3, for all players, the variance of the posterior distribution is smaller than that of the prior, which suggests that the choice of prior distributions was compatible with the likelihood. The average posterior standard deviation was 0.064 for both offense and defense. Furthermore, note that the posterior variances are much smaller for the replacement players (except for offensive Position 2, which only contained five replacement players). If there were a lot of heterogeneity in the team rebounding ability of all the replacement players who were grouped together, one would expect the variance of the respective replacement player parameter to be larger than if there was homogeneity. Therefore, the fact that the posterior variances are small suggests that the replacement player grouping was appropriate.

Further note that despite the fact that the distribution of prior means was heavily positively skewed, the posterior means appear to be symmetrical and roughly Normal. This is in line with how one would expect traits to be distributed within a population: if one thinks as team rebounding ability being a linear combination of many latent variables (like strength, size, positioning, effort, age, etc.), the Central Limit Theorem suggests that the combination of the factors should be approximately Normal.

One final feature worth exploring is whether the estimated parameters (which are given by the posterior means) tell a different story than the empirical rebounding rates. For otherwise, one might just as well directly use individual rebounding rates to measure contributions to team rebounding. To verify this, Kendall’s tau between the posterior means and the empirical individual rebounding rates (i.e., the prior means) was computed; its value was found to be 0.51 and 0.41 on defense and offense, respectively. This further supports the idea that individual rebounding rates don’t tell the whole story when measuring contributions to team rebounding. This also suggests that the prior variances were not too small, because the likelihood function clearly plays a part in the stochastic ordering of the posterior distributions.

#### 4.1.2 Gamma-level

The average posterior mean is equal to 1.148 for the defensive parameters and −0.785 for the offensive parameters. The shift between offensive and defensive parameters has a practical explanation: there are a lot more offensive team rebounds than there are defensive team rebounds, because blocked shots are often swatted out of bounds. These values suggest that for the average defensive lineup, only about 6 % of defensive rebounds are team rebounds, whereas for the average offensive lineup, that number skyrockets to about 30 %, which is inline with the empirical rates of 5.6 % and 25.1 %, respectively. This quirk of the data, and its practical implications, are discussed further in Section 5.1.

**Figure 4:**
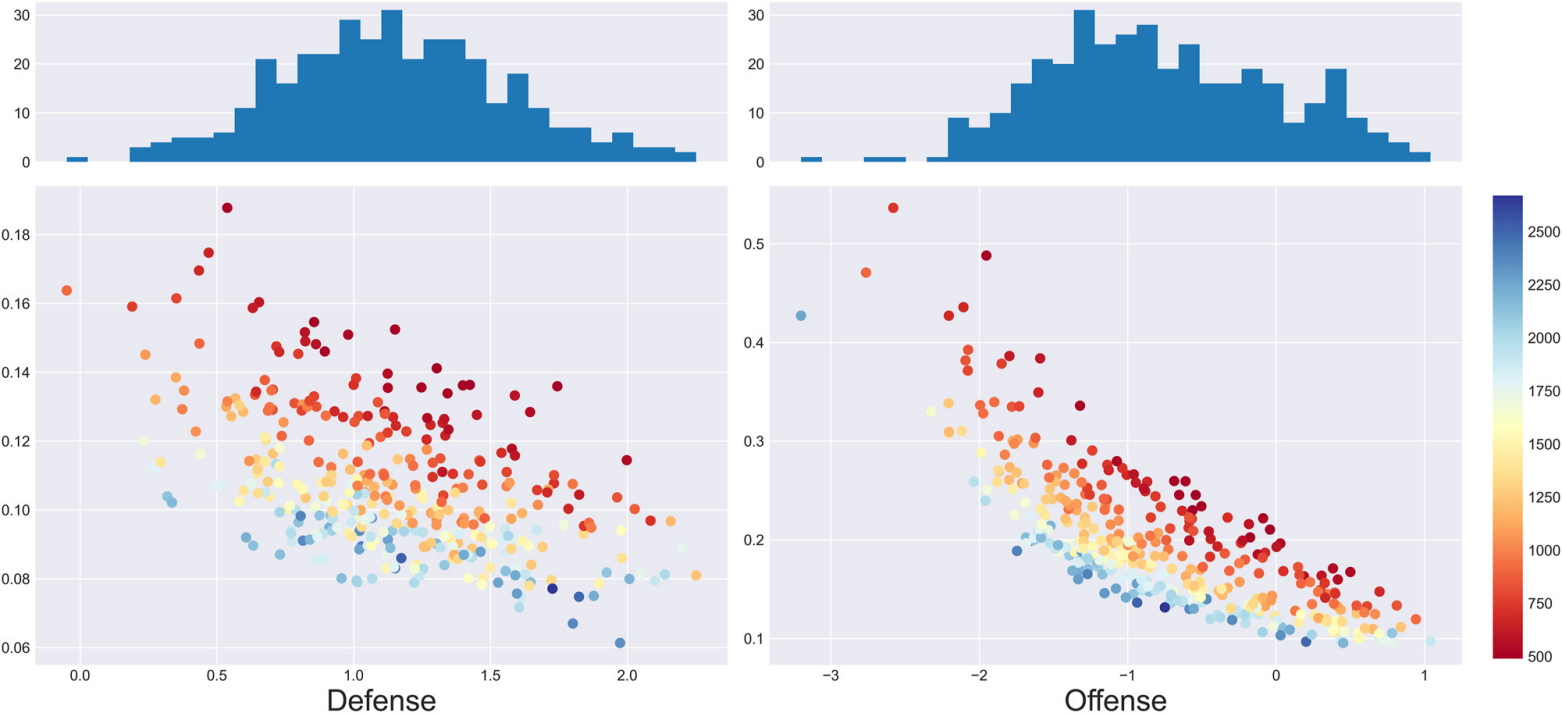
Standard deviations (*y*-axis) of each posterior distribution against the posterior mean (*x*-axis) of the *γ*-level parameters. Color represents the number of minutes of the player in question, and is a proxy for the number of multinomial observations used to estimate the parameter. Replacement players were omitted due to their much lower standard deviations, which made the mean-variance relationship less apparent.

As illustrated by Figure 4, the posterior variances exhibit a lot more heterogeneity than at the *β*-level. First, at the *γ*-level, offensive posterior distributions generally have larger variance than the defensive posterior distributions. This makes sense: considering that estimation is performed conditionally on which team has collected the rebound, and given that there are a lot more defensive rebounds than offensive ones, one ends up using far fewer samples for the estimation of the offensive parameters than for the estimation of defensive ones. Furthermore, there is an obvious negative relationship between the posterior mean and variance. This has to do with the flatness of the logistic function: because conditional individual rebounding rates tend to be lower than 50 %, very small values of *γ*-level parameters are nearly indistinguishable from each other as they lead to nearly identical probabilities. In other words, although it may be difficult to compare poor *γ*-level rebounders, one can rest easy knowing that they won’t be getting the ball anyways. Further note that the posterior means look roughly normally distributed, which makes intuitive sense.

### 4.2 Model validation

To make sure that the model is picking up a meaningful signal, predictions were made for the 2021–22 NBA season using the estimated parameters. By virtue of having a new season, one has new players introduced into the dataset from two principal sources: rookies who were just signed to their first NBA contract, or players who were formerly replacement players, but who saw a significant increase in playing time relative to 2020–21. This second group was a combination of established players who were returning from long-term injuries (such as Spencer Dinwiddie or Jaren Jackson Jr.), and players who had improved enough to warrant more playing time (such as Isaiah Joe or Gary Payton II).

Although one could have just used the replacement player parameters to predict rebounding rates in such lineups, in general, this did not seem like an adequate assessment of model fit: a superstar coming back from injury or a first overall pick are probably not comparable to a player who is signed to a ten-day contract as an injury replacement. Given that the main goal is to assess rebounding ability, and that the purpose of predicting is to ensure that one hasn’t just picked up random noise in the training data, the decision was made to predict only in instances where all players on the court were non-replacement players in the training set. Thus predictions were made on approximately 25,000 missed shots, which represented a bit more than 20 % of all shots missed during the 2021–22 season.

Within this subset of “predictable” data, one can further distinguish between two types of samples: seen lineups and unseen lineups. Seen lineups represent lineups where the exact five-man defensive lineup combination also appeared in the training set. A distinction is made between these two types of samples so that one can better detect over-fitting: given the data limitations and the simplicity of the model, it seems likely that formal assessment of fit would deem the model inadequate. However, if the model performs far better in seen lineups than unseen ones, the model was probably over-fitted to the training data.

The testing dataset contains about 19,000 instances with unseen lineups, and about 6,000 instances with seen ones. Furthermore, when assessing fit graphically, on top of plotting observed and predicted counts based on the groups used for the Hosmer–Lemeshow test (Hosmer et al. 2013), one can also sum predictions over teams and players, to allow for a more practically interpretable assessment of fit.

#### 4.2.1 Team-level predictions

**Figure 5:**
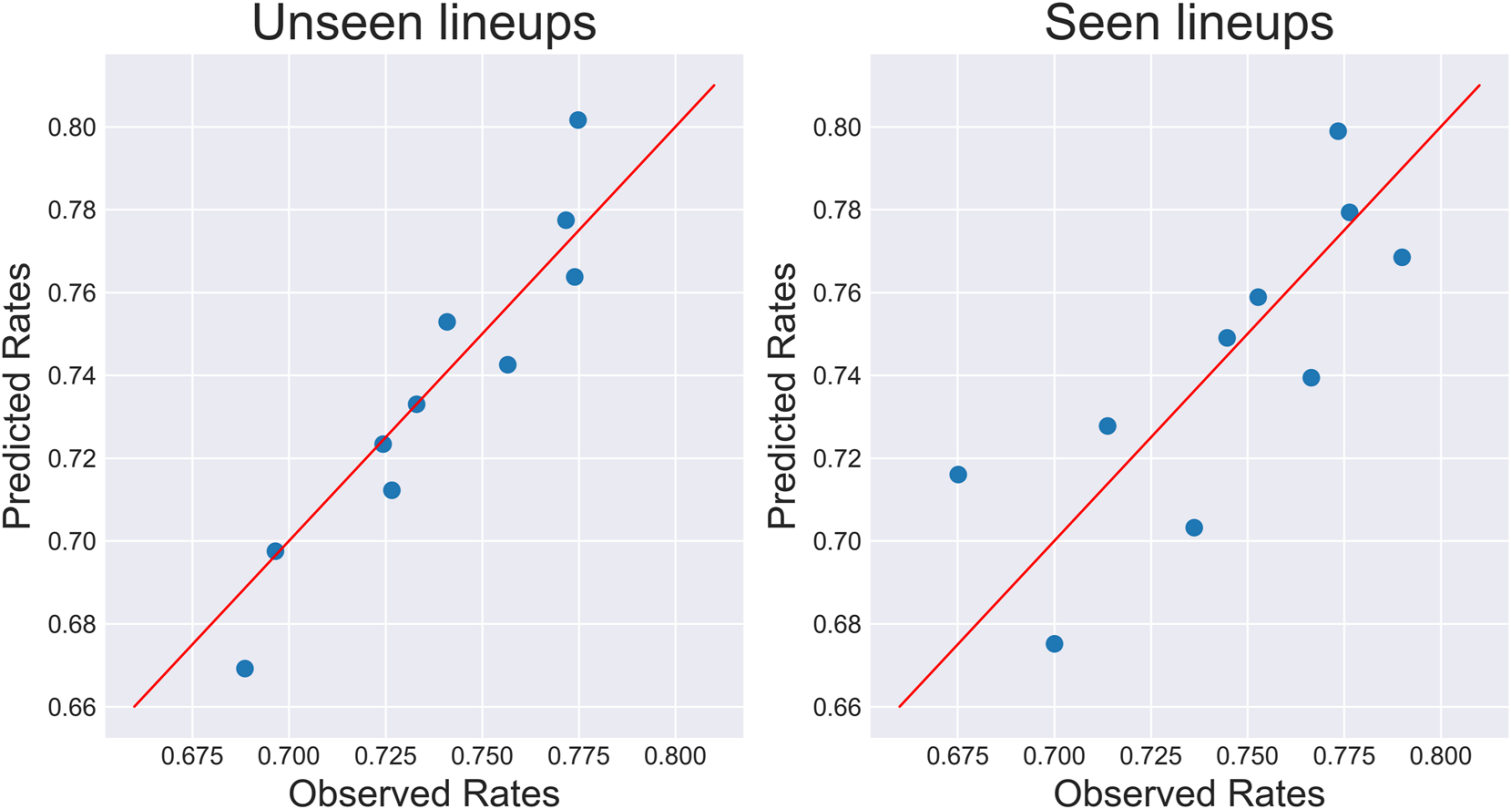
Predicted rates (*y*-axis) against observed rates (*x*-axis) for seen and unseen lineups during the 2021–22 NBA season. Note that the groups for the unseen lineups contain each about 1940 observations, and the seen lineup groups contain about 560 observations.

Figure 5 shows the observed team rebounding rates against the predicted ones, for the 10 groups used to conduct the Hosmer–Lemeshow test (note the differences in group sizes). Visually, it does not seem as though there is any over-fit to the training data, and the formal test seems to support that impression.

When testing the hypothesis that the observed and predicted rates are identical, the *p*-value for the unseen lineups is approximately 0.015, and is 0.0483 for the seen lineups, which seem comparable given the difference in sample size. However, the subjectivity of the binning scheme makes formal assessment of fit difficult: by increasing the number of groups from 10 to 11, the *p*-values change to 0.043 and 0.024, respectively. Furthermore, if the number of groups is increased to 20, for both observation types, the null fails to be rejected at the 5 % level. In short, although the model doesn’t seem to explain all the rebounding variability, it does seem to have at least captured some meaningful information.

**Figure 6:**
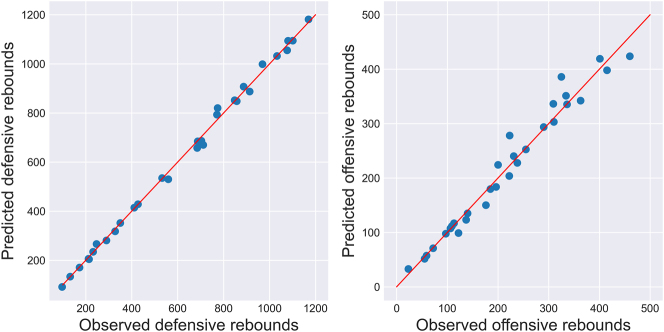
Predicted versus observed rebounding counts for each of the 30 teams during the 2021–22 NBA season (in all predictable instances).

One can also compare predictions aggregated across teams, which are shown in Figure 6, for a more practically interpretable assessment of fit. Also note that the drastic difference in predictions is due to the variable number of replacement players found across all teams: for example, the Houston Rockets decided to rebuild, which means that most of their players were rookies and hence have very few predictable instances, whereas the Los Angeles Lakers made a point of acquiring established veteran players, which effectively means that all of their missed shots were predictable. The global performance of the team-rebounding model appears to be reasonably good.

#### 4.2.2 Two-stage individual rebounding predictions

An attempt was further made to predict individual rebound allocation. For each missed shot, the expected number of rebounds was computed for every player on the court. For each player in the testing dataset, expected rebounds were then summed up across all lineups they appeared in, and compared to the observed counts across those same lineups.

**Figure 7:**
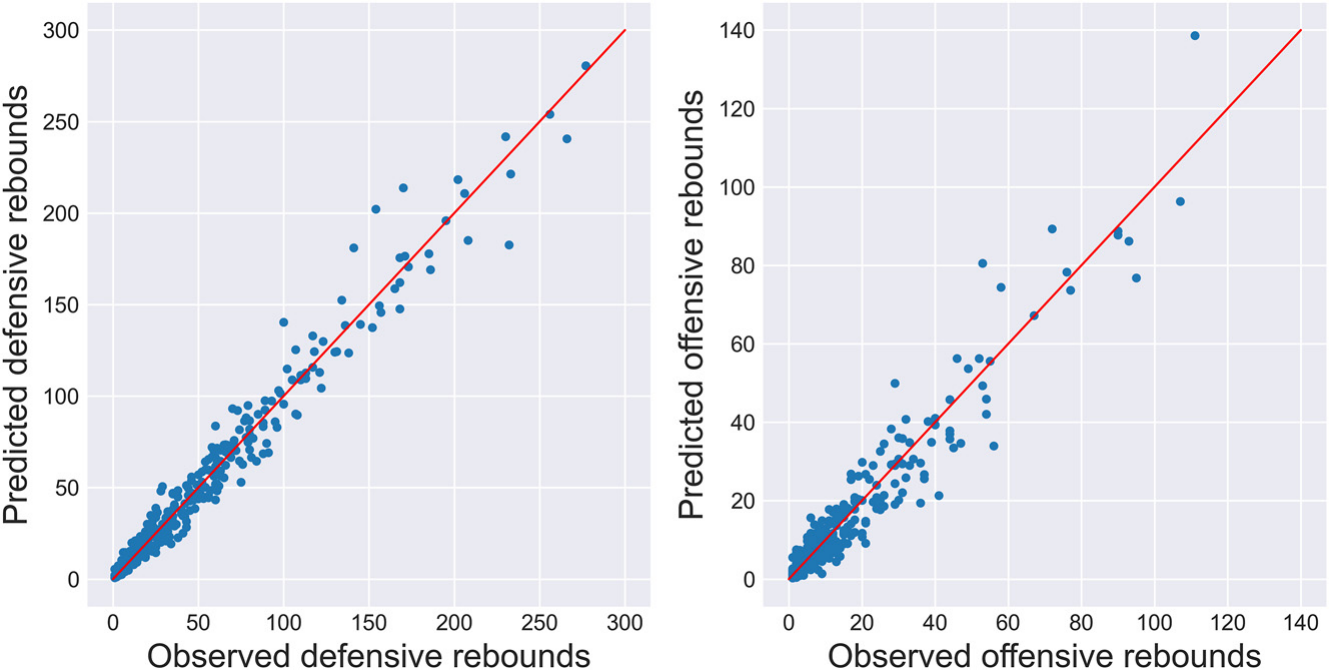
Two-stage predicted versus observed rebounding counts for individual players during the 2021–22 season. Note that about ten players were omitted from the offensive plot because they had far more offensive rebounds than those plotted, so their inclusion in the plot “squished” everyone else together. The fit for those players was comparable to the players retained for plotting.

The resulting scatter plots are shown in Figure 7 for both defensive and offensive rebounds. Given the greater uncertainty in the offensive individual rebounding parameters and the much smaller number of multinomial trials in the offensive case, it is not surprising that the fit is poorer in the latter than in the former. Nevertheless, one can see that the expected and observed rebounding counts are in general agreement, further suggesting that the proposed methodology behaves in an acceptable way.

#### 4.2.3 Player rebounding assessment

**Figure 8:**
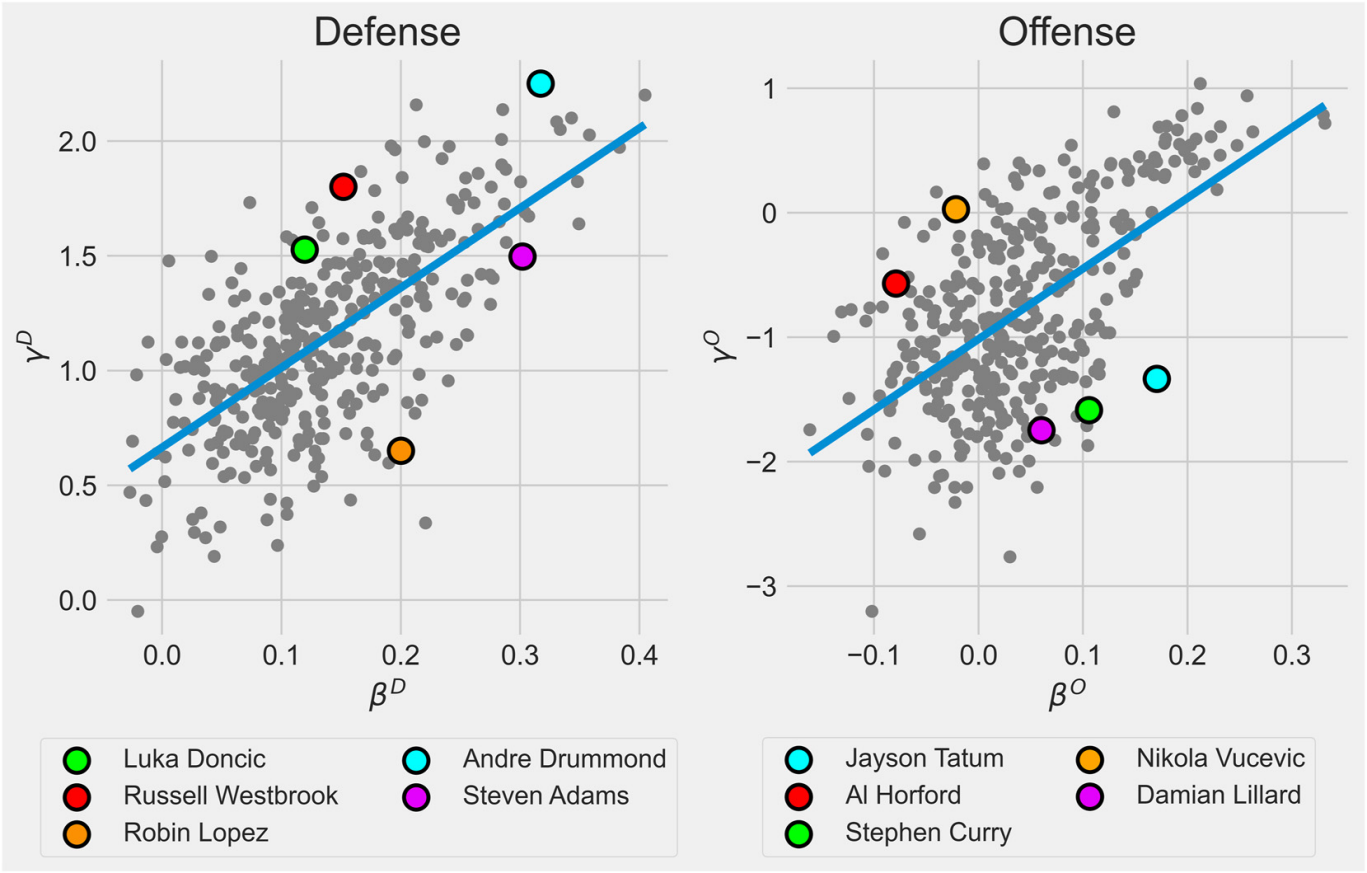
Scatter plot of estimated *β* parameters against their corresponding *γ* parameter. The blue line is the best fitting line. Players in the top left are overvalued when evaluated using individual rebounding rate, whereas players in the bottom right are undervalued.

Given the goal of accurately assessing the players’ “true” ability to steal rebounds from opponents rather than from teammates, a scatter plot of estimated *β*-level parameters against estimated *γ*-level parameters is provided in Figure 8. The idea is that players at the top left are overvalued because they collect a disproportionate amount of rebounds relative to their *β*-level contributions. The opposite is true for players found at the bottom right.

For example, Andre Drummond has a reputation for being an overvalued defensive rebounder due to the large number of uncontested rebounds he collects. The model suggests that this view is at least somewhat correct. Furthermore, Steven Adams has a reputation for being an undervalued defensive rebounder because of his willingness to let his teammates collect rebounds. Again, this view is supported by the model. The posterior means for team rebounding parameters and individual rebounding parameters for all players are given in Part B of the Online Supplementary.

Given that the parameters were on different scales, a more formal measure of discordance was computed by subtracting the *β*-level parameter rank from the *γ*-level parameter rank. One interesting thing to note when looking at the full discordance rankings is that there are clearly some player archetypes which are consistently overvalued or undervalued (their discordance value is given in parentheses). In general, it seems that long-range threats, like Desmond Bane (−200), Davis Bertans (−202) or Damian Lillard (−203), positively impact their team’s offensive rebounding far more than their individual rates would suggest. Perhaps this is due to the fact that their shooting ability forces opposing defenders onto the perimeter and away from the basket, which allows their teammates to collect offensive rebounds for themselves more easily.

Furthermore, there appears to be a subset of centers that could be overvalued on the offensive glass, such as Willie Cauley-Stein (256), Nikola Vucevic (222.5) or Al Horford (208). A plausible explanation is that regardless of rebounding ability, the center will collect a significant amount of offensive rebounds by virtue of occupying prime rebounding real estate, which sounds intuitively reasonable.

On the defensive end, there is a trend of ballhandlers generally being overvalued, such as Russell Westbrook (125.5) or Devin Booker (168), for example. Perhaps teams are “artificially” funnelling more rebounds to their ballhandlers, so that they can more efficiently begin their fast break, or maybe these players are matched against opposing perimeter players, meaning that they have fewer boxing out responsibilities. Furthermore, Table 1 lists leaders at both the *β*- and *γ*-level, and Table 2 reports the players with the largest positive difference and the largest negative difference between both levels.

**Table 1:** *β*-level and *γ*-level leaders.

*β*-level off.		*γ*-level off.		*β*-level def.		*γ*-level def.	
Name	Param. value	Name	Param. value	Name	Param. value	Name	Param. value
J. Valanciunas	0.332	C. Capela	1.040	J. Valanciunas	0.405	A. Drummond	2.252
E. Freedom	0.330	M. Brown	0.942	N. Vucevic	0.383	J. Valanciunas	2.201
D. Howard	0.262	A. Drummond	0.840	J. Nurkic	0.358	D. Howard	2.159
M. Brown	0.257	W. Hernangomez	0.813	I. Zubac	0.349	G. Antetokounmpo	2.137
M. Robinson	0.247	E. Freedom	0.785	K. Love	0.348	C. Capela	2.101

**Table 2:** Most extreme discordances between *β* and *γ* level parameters.

Off. Disc. | largest pos.		Off. Disc. | largest neg.		Def. Disc. | largest pos.		Def. Disc. | largest neg.	
Name	Rank diff.	Name	Rank diff.	Name	Rank diff.	Name	Rank diff.
D. Jones Jr.	268	K. Lewis Jr.	−267	R.J. Hampton	279	I. Okoro	−303
W. Cauley-Stein	256	F. Korkmaz	−250	T. Craig	260	R. Lopez	−243
E. Paschall	246	J. Tatum	−245	C. Boucher	258	R. Neto	−242
M. Carter-Williams	238	S. Curry	−237	T. Herro	214	J. Harris	−223
K. Oubre Jr.	237	E. Bledsoe	−236	N. Noel	212	A. Wiggins	−221

#### 4.2.4 Example: the Timberwolves acquire Rudy Gobert

To illustrate the relevance of the model, consider the following hypothetical situation: suppose the Minnesota Timberwolves feel like their defensive rebounding needs to be improved after the 2020–21 season. They consider replacing Jarred Vanderbilt, whose defensive rebounding rate is 21.1 %, with Rudy Gobert, whose defensive rebounding rate is 28.8 %. Below is an exploration of the impact of this change on their most frequent lineup during the 2021–22 season against average offensive rebounding competition. The relevant estimated parameter values are given in Table 3.

**Table 3:** Estimates of parameters ***β***_***D***_ and ***γ***_***D***_ for Rudy Gobert and the players on the most common Timberwolves lineup from the 2020–21 season.

Player	*β*_*D*_	*γ*_*D*_
Patrick Beverley	0.1257	1.0883
Anthony Edwards	0.1092	1.0665
Rudy Gobert	0.2843	2.0078
D’Angelo Russell	0.0422	0.5959
Karl-Anthony Towns	0.2540	1.7681
Jarred Vanderbilt	0.1254	1.7108

Using the fact that the average estimated offensive team parameter across the league is 0.0406 and the estimated intercept term is 0.535 (see Section 4.1), one finds that before swapping Vanderbilt out for Gobert, the predicted lineup defensive rebounding rate is 72.8 %, and increases to 75.9 % after the acquisition, far less than the direct difference between their individual defensive rebounding rates.

An especially noteworthy fact is that, in spite of such a move making sense from a team rebounding perspective, looking solely at predicted individual defensive rebounding rates suggests that the acquisition does not make sense: the predicted individual rebounding rate of Towns goes from 21.3 % to 20.2 %, and Gobert’s predicted rate after the acquisition is 25.7 %. When this trade ended up actually being made after the 2021–22 season, the observed individual defensive rebounding rates for both Towns and Gobert showed a comparable decline. This example further highlights how rebounding rates should not be used without care in assessing a player’s rebounding abilities and contributions.

## 5 Discussion

### 5.1 The subtle misleadingness of rebounding numbers

Although rebounding is obviously a coveted skill amongst NBA players, simply measuring team-level rebounding ability may obfuscate “practical” rebounding ability. For example, consider two players, A and B, who are identical when it comes to corralling down a missed shot. However, assume that player A is an excellent shot blocker, whereas B is a terrible one.

In a practical sense, it is natural to expect any measure of rebounding ability to value these two players equally as they are identical at collecting missed shots. But given that most blocked shots are sent out of bounds (recall that a missed shot sent out of bounds results in a team rebound for the inbounding team), in a technical sense, A is a worse defensive rebounder than B because A is generating offensive rebounds for the opposing team. We note that some well-known shot blockers, such as Rudy Gobert, Richaun Holmes, and Hassan Whiteside, have surprisingly low *β*-ability estimates, perhaps due to this quirk.

One possible remedy to this problem is to simply remove blocked shots from rebounding opportunities, but one ends up with a similar issue: if a shot blocker is able to keep the ball inbound, and tip it to a teammate, should they not be considered a superior rebounder? This appears to be the case for some great shot blockers, like Clint Capela and Jakob Poeltl, who are flagged as great *β*-level rebounders by the model.

On the offensive end, there is potential for the opposite problem to occur: perhaps there are players who are “extremely good at getting blocked out of bounds,” and who are therefore technically superior *β*-level offensive rebounders. This is perhaps the case for players like Eric Bledsoe, Kira Lewis Jr. or Ja Morant: the model views these players as surprisingly good *β*-level offensive rebounders, but they all love to attack the basket and are not afraid to challenge players at the rim and force defenses out of their ideal rebounding positions.

As this was a data collection issue rather than a modeling one, it was ignored for estimation purposes, but by preparing the data with this in mind, the methodology outlined above would be directly usable. Future work could perhaps explore how to isolate rebounding ability even further. For the time being, caution should be exerted regarding player valuations in the case of exceptional shot blockers and “blockees.”

### 5.2 Validity of the constant rebounding ability assumption

The idea that team rebounding can be explained solely as an interactionless combination of players on the court is almost certainly false. For example, one popular strategy to mitigate the effectiveness of Rudy Gobert has been to force him to guard capable three-point shooters, hence forcing him away from the basket and impacting his defensive rebounding. This suggests that there is an important interaction between offense and defense that is being ignored. Obviously, modeling all such interactions is not tractable given the very limited amount of data, and is omitted in most APM-based approaches. One potentially feasible way to incorporate these interactions would be to include an interaction term based on the positions of the players in question, and assuming that the interaction is identical across all players of the given positions.

Furthermore, although the idea that all players have some constant intrinsic value of rebounding ability is probably approximately true, players may adapt their play-style based on lineup composition and thus, may have a different value for their rebounding ability depending on the specific lineup. For example, despite the model predicting an increase in individual rebounding rate for Draymond Green when the Golden State Warriors play him in place of Kevon Looney, it is probably the case that Green’s contributions to team rebounding are underestimated in that specific lineup, as he is likely more aggressively pursuing rebounds than he would if Looney were still on the court. Accounting for such a difference is obviously impossible given the available data. Therefore it is suggested that model predictions perhaps be viewed as a “lower bound” on team rebounding ability.

### 5.3 Conclusions

This paper introduces a Bayesian framework to identify players who help their team win the rebounding battle, regardless of their individual rebounding totals. By carefully choosing the prior structure, a tractable model was obtained that can simultaneously estimate offensive and defensive rebounding ability. It is hoped that beyond filling a void in individual rebounding assessment, the proposed methodology can improve player evaluation, especially given that play-by-play data are available in most leagues. Should the approach be implemented in a practical setting, rebounds following blocked shots should be handled carefully.

## Supplementary Material

Supplementary Material Details

## Figures and Tables

**Figure A1:**
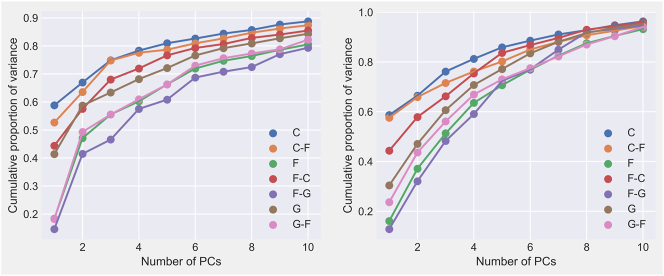
Left: scree plot for average variance proportion retained for a given position in offensive dataset. Right: scree plot for average variance proportion retained for a given position in defensive dataset. The labels are those used by the NBA.

**Figure A2:**
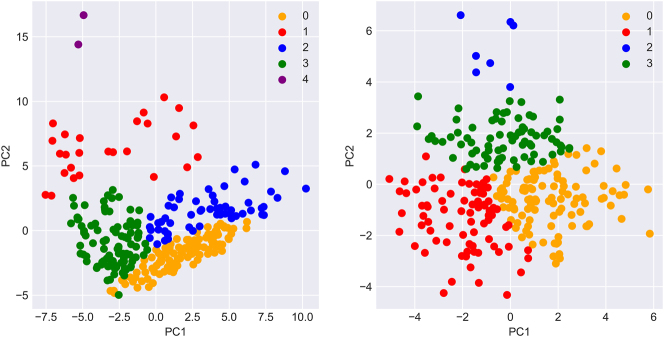
Left: scatter plot of players showing position clusters on offense. Right: scatter plot of players showing position clusters on defense. Players were assigned to their most probable cluster.

**Figure A3:**
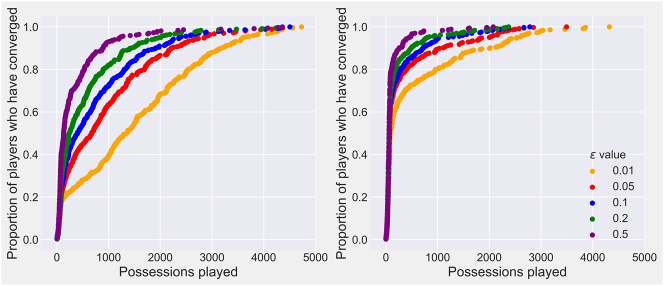
Left: league-wide convergence rates for offensive non-centers. Right: league-wide convergence rates for defensive non-centers.

## Appendix: Player clustering

In this Appendix, an approach to reducing the dimension of the parameter space is described. It is inspired by the “Replacement Player” approach introduced by Woolner (2002).

Recall that NBA positions are subjective and especially not reliable for players seeing limited game time. Henceforth the following two terms are distinguished: the “position label” is the position that a player is assigned in the NBA dataset; in contrast, the “underlying position” is the latent position of a player that one is interested in learning. Moreover, the following assumptions are made:

A1) The first grouping heuristic is that there exist separate offensive and defensive positions.
A2) The second grouping heuristic is that the position labels do convey meaningful position information in the aggregate: although there may be some “mislabelled” players (especially among players who have played very little), players who share the same underlying position are more likely to end up with the same position label. Moreover, it is assumed that the ordering in compound position labels also conveys information about the underlying position, and hence permutations are treated as a unique label. Therefore, the seven position labels relied on are G, G-F, F-G, F, F-C, C-F, and C.
A3) The third grouping heuristic is that the set of possible underlying positions is the same for all players, regardless of how much playing time they get: this makes it possible to learn the underlying positions from players who have a large sample of games played, and then assign these labels to unusable players.
  This third heuristic is important because the small sample sizes of unusable players mean that their features are often extreme, and make them unsuitable for clustering. Because of this, only players having played in at least 1,000 possessions are retained for “positional learning.” This cutoff divided the dataset into 366 usable players and 174 unusable players.
A4) The final grouping heuristic is that there exists a traditional center underlying position on both offense and defense, and that in general, this underlying position is very easy to identify compared to other positions, meaning that the C position label is more reliable.
  This heuristic is based on the fact that the traits of the traditional center are well captured by the dataset, given that measurements like average shot distance, shots blocked, or lack of three-point shots are recorded. Henceforth, when distinguishing between the traditional center position and the other positions, they will be referred to as “centers” and “non-centers,” respectively.

## A1 Reformatting the dataset

Given the objective of clustering by play style over the course of the season, all performances belonging to a given player were grouped together as follows:

a) Count variables were summed together and were scaled to be per 100 possessions.
b) Game summary variables (like average game speed, for example) were combined into a weighted average, where weights depended on the number of possessions in the corresponding game.

These raw player tendency datasets (offensive and defensive variables are handled separately) cannot be used directly to “learn” the underlying positions of each player: there is significant correlation between the columns of the feature matrices. This can be problematic when dealing with generalized mixed models (GMMs), because the re-assignment of points to a cluster can be unstable and vary wildly between iterations. The most obvious solution to this problem is to reduce the dimension of the feature matrices by performing principal component analysis (PCA), and retaining only some subset of the principal components. This obviously prompts the question of how many principal components should be retained for classification, which will be discussed in Appendix A2.

Performing PCA on the normalized dataset appears to work well if one naively looks at the proportion of variance retained for the whole dataset. However, as illustrated in Figure A1, this is slightly misleading: some position characteristics are over-represented in the dataset because of the ease with which they can be recorded. For example, there are multiple features relating to posting up, but very few related to shooting three pointers. This issue becomes obvious when looking at the proportion of variance retained for each position label. Therefore, if one were to reduce uniformly the dimension for all position labels, one would require a lot of principal components to keep all the data from being “squished” together.

## A2 Clustering approach

Because of this “squishing” phenomenon, an iterative approach was implemented:

1. Perform PCA on the whole dataset.
2. Learn to separate centers and non-centers (based on their underlying position) using some subset of the principal components.
3. Remove underlying centers from the dataset.
4. Perform PCA on the original features of the non-centers.
5. Learn the different positions in the reduced dataset using some subset of the principal components.

This approach requires the choice of two hyper-parameters: the number of mixture components in the GMM, and the number of principal components retained. Also, note that the procedures below were repeated for both the offensive and defensive datasets.

### A2.1 Learning traditional centers

For the first iteration of clustering, one need only determine the number of principal components to retain, as one must obviously have exactly two mixture components: centers and non-centers. The number of principal components to retain was determined fitting a GMM to 1, …, 10 principal components, and then for each model, the *F*-score was computed by matching with the NBA labels, and then simply picking the number of components which led to the greatest score.

The positions labels of C, F-C, and C-F were all considered centers when computing the *F*-score, and all other positions labels were considered non-centers. Also note that because GMMs do not explicitly assign observations a label, to determine which cluster corresponded to the center cluster, the cluster containing the most players with a position label of center was considered to be the center cluster. Therefore, when it came to computing the *F*-score, all players who had an estimated center cluster probability membership larger than 0.5 were predicted as centers, and all other players were predicted to be non-centers.

For the offensive dataset, one principal component was unequivocally found to be most appropriate, with an *F*-score of 0.837. In practical terms, this clustering also seemed appropriate: some noteworthy false positives were Derrick Favours, Richaun Holmes, and Kevon Looney, and some notable false negatives were Lauri Markkanen and Kelly Olynyk. Note that the terms false negatives and false positives are used here to denote players who were clustered incorrectly based on their NBA position label.

As one would expect, the defensive dataset was a lot fuzzier: *F*-scores were nearly identical for one, two, three, and four principal components (all hovered around 0.84), before suffering a major drop-off. For purely practical reasons, one principal component was retained. In this case, there were some notable false positives, namely Blake Griffin, Serge Ibaka, and Kevon Looney, whereas some notable false negatives were Aleksej Pokusevski and Larry Nance Jr.

Tables containing all the false positive and false negative centers can be found in Part C of the Online Supplementary. Tables containing all centers classifications (based on cluster probabilities) can be found in Part D of the Online Supplementary.

### A2.2 Learning the other positions

The current clustering context is slightly different from traditional problems:

a) One must determine both the clustering parameters and the number of features (i.e., principal components) to retain for clustering.
b) There is some useful, but not entirely accurate, label information that one would like to exploit.

In traditional applications, a common method for picking the number of mixture components (which in this case, corresponds to the number of latent true underlying positions) in a GMM is to use the Bayesian information criterion (BIC). Given that the BIC of a model is a function of the likelihood, it can be used to determine the appropriate number of clusters for some fixed amount of principal components, but it is unsuitable for determining the number of principal components to retain, because the likelihood will decrease when additional principal components are added. Directly using BIC would also ignore completely the partial label information.

To determine how many principal components to retain, an assessment of fit is required that is independent of the likelihood. This is why the second modeling heuristic, i.e., that sharing an underlying position increases the likelihood of sharing a label, is key. First, the following shorthand is defined:

$$\mathrm{Pr}\left(\ell_{k}|P_{j}\right)=\mathrm{Pr}\left(\text{Having label }\ell_{k}|\text{Underlying position }P_{j}\right).$$

Assume that one has some arbitrary assignment of players into clusters, and let $\pi_{j}^{\left(i\right)}$ denote player *i*’s probability of being assigned position *P*_*j*_. Based on the heuristic that labels convey meaningful information in the aggregate, one would expect that for an arbitrary underlying position *P*_*j*_, there exists some label *ℓ*_*k*_ that is much more prevalent among players with underlying position *P*_*j*_, or more formally, that there is some label *ℓ*_*k*_ such that Pr(*ℓ*_*k*_∣*P*_*j*_) ≫ Pr(*ℓ*_*h*_∣*P*_*j*_) for all *h* ≠ *k*.

For some proposed clustering (i.e., proposed set of underlying positions) with *n*_*j*_ players in cluster *j*, one can define the score

$$S=\overset{\text{Positions}}{\underset{j=1}{\sum }}n_{j}\;S_{j},$$

where $S_{j}=\mathrm{Pr}\left(\ell_{k_{1}}|P_{j}\right)-\mathrm{Pr}\left(\ell_{k_{2}}|P_{j}\right)$ and

$$\ell_{k_{1}}=\arg\max_{\text{Labels}}\mathrm{Pr}\left(\ell_{h}|P_{j}\right),\;\ell_{k_{2}}=\arg\max_{\text{Labels}\backslash \ell_{k_{1}}}\mathrm{Pr}\left(\ell_{h}|P_{j}\right).$$

This score basically favors assignments of clusters that have one predominant label for each cluster because the gap between the most popular class and the second most popular class is being maximized. In other words, maximizing the score maximizes the homogeneity within clusters.

Although calculating these conditional probabilities exactly would require knowing cluster membership, one can estimate them using the predicted cluster membership probabilities, along with Bayes’ rule, viz.

$$\mathrm{Pr}\left(\ell_{h}|P_{j}\right)=\frac{\mathrm{Pr}\left(P_{j}|\ell_{h}\right)\mathrm{Pr}\left(\ell_{h}\right)}{\mathrm{Pr}\left(P_{j}\right)}=\frac{\overset{\text{Label}\;\ell_{h}}{\underset{i}{\sum }}\pi_{j}^{\left(i\right)}}{\overset{\text{All players}}{\underset{i}{\sum }}\pi_{j}^{\left(i\right)}}.$$

To obtain an overall score for the proposed clustering, one can then simply compute a weighted average of cluster scores, weighted by the number of players within the respective cluster.

Given that the EM algorithm depends on cluster initialization, observe that the clustering can differ from one iteration to the next, meaning that every iteration can potentially have a different score, a different likelihood, and different model parameters, for a fixed amount of principal components and mixture components. Ideally, one would like a model with a large score (as defined above) and a large likelihood relative to iterations with the same number of principal components and mixture components (otherwise these are not directly comparable). To pick the optimal classification, Algorithm A1 was performed.

Algorithm A1. An algorithm for picking a suitable classification.

**Table:**

**for** *i* in 1, …, 8 **do**
**for** *j* in 2, …, 6 **do**
**for** *k* in 1 to 100 **do**
Fit a GMM using *i* principal components and *j* mixture components
and a random set of initial values
Predict the cluster probabilities.
Compute the score.
Compute the likelihood.
**end for**
Rank the likelihood values for the 100 fits $\left(R_{L}^{k}\right)$ .
Rank the scores of the 100 associated cluster assignments $\left(R_{S}^{k}\right)$ .
Retain the clustering with the highest average rank $\left(R_{L}^{k}+R_{S}^{k}\right)/2$ .
**end for**
**end for** Of all the retained models, pick the one with the highest average rank.

Using this procedure, the optimal offensive clustering had an average rank of 99, and the optimal defensive clustering had an average rank of 99.5. Furthermore, the clusterings proposed by the final models seemed heuristically correct.

### A2.3 Clustering results and interpretation

The clustering procedure suggested that there are five underlying offensive positions and four underlying defensive positions (excluding centers). A scatter plot of players assigned to non-center positions is given in Figure A2. A full list of all non-center classifications is given in Part E of the Online Supplementary. A short practical interpretation and assessment of the clustering is briefly given here.

Heuristically speaking, the offensive clustering seems quite appropriate. Specifically, the following interpretation of offensive clusters can be made.

a) Position 0 seemed to contain “shooters with a bit of a handle,” i.e., perimeter players who are capable of handling the ball, but that aren’t generally the main ball handler on their team. Some notable examples were players like Eric Bledsoe, Jordan Poole, and Anfernee Simons.
b) Position 1 seemed to contain “multilevel” players, i.e., players who tend to operate all over the floor. Although the model assigned all these players the same position, it is interesting to note that Position 1 shows what appear to be sub-clusters. The left sub-cluster contained “stretch-bigs,” like Al Horford, Brook Lopez or Christian Wood, whereas the right sub-cluster contained players who operate at all three levels by slashing to the basket, like Jimmy Butler, Kevin Durant, or LeBron James.
c) Position 2 contained “offensive initiators,” i.e., scoring threats who handle the ball a lot, like Luka Doncic, Ja Morant, or Trae Young.
d) Position 3 seemed to contain players who operated exclusively on the perimeter, without handling the ball much, such as Trevor Ariza, Maxi Kleber, and Mike Muscala.
e) Position 4 contained exclusively Giannis Antetokoumnpo and Zion Williamson. This agrees with the sentiment that they are (nearly) one-of-a-kind players.

Players also appeared to be appropriately placed on the cluster boundaries: for example, Steph Curry and Damian Lillard were both classified as Position 2, but both had a pretty sizeable probability of belonging to Position 0, which coincides with their willingness to play off-ball.

Although the defensive clustering was interesting, it was a lot fuzzier than the offensive clustering.

a) Position 0 contained what can only be described as “limited defenders” such as Eric Gordon, Ja Morant, and Trae Young.
b) Position 1 contained defenders who rely on their size, length and wingspan, such as Robert Covington, Pascal Siakam, and Grant Williams.
c) Position 2 was by far the most interpretable cluster: it aggregated all the defensive pests with a knack for stealing the ball, such as Alex Caruso, T.J. McConnell, and Matisse Thybulle.
d) Position 3 contained defenders who rely on foot speed to defend on the perimeter, like Jevon Carter, De’Anthony Melton, and Marcus Smart.

Given that a big part of defense is about limiting the offensive players’ ability, it is obviously quite difficult to describe a defensive performance with count data. One clear avenue for improvement would be to use tracking data, as suggested by Bornn et al. (2016). Alas, such data are no longer publicly available.

### A2.4 Assigning unusable players a position

Recall that the aforementioned procedure is just for learning the positions from players having accumulated enough playing time. It does not actually involve these unusable players. Below, ways to assign unusable players a position are explored.

For unusable players who are close to the 1,000 possession cutoff, assigning a position is straightforward: one can simply classify their feature vector, and pick the cluster with the largest probability. For players who are nowhere near the cutoff, this approach is more problematic because their “per 100 possessions” stats can be far too extreme. For example, some players had fewer than 20 possessions during the season, which means that if they were to record a single block, they would by far be the greatest shot blocker in the dataset. For these extreme cases, one has no choice but to rely on their labels.

To handle both these cases simultaneously, the following high-level idea is proposed: the assigned underlying position should be a weighted average between the player’s direct classification probabilities and the mean classification probabilities of their label. The more a player has played, the more weight should be placed on their direct classification probability, and vice versa.

To determine how long a player must play before their position classification can be considered accurate, it is useful to return to the training set of usable players, and study how long it takes for players to be correctly classified under the assumption that their final classification is correct. For a given player *i*, let *π*_*i*_ denote their end-of-season classification probabilities, and let *Q*_*i*,*t*_ denote their classification probabilities using their running feature average at time *t*. It is said that a player has converged by time *T* if, for some given *ϵ* ∈ (0, ∞),

$$\sup_{t>T}D_{\text{KL}}\left(\pi_{i},Q_{i,t}\right)<\epsilon ,$$

where *D*_KL_ denotes the Kullback–Leibler divergence.

Figure A3 shows time to convergence results for the non-center positions. It is obviously difficult to choose objectively an *ϵ* value to define convergence. Therefore, it was decided heuristically that 1,000 possessions was in fact a sufficient cutoff for both offensive and defensive non-centers: for all reasonable choices of *ϵ*, a large proportion of players have converged, and this choice also allows one to remain consistent with the initial definition of unusable players. The same procedure was performed for the center position. In line with these heuristics, time to convergence was much shorter when dealing with centers: 500 possessions seemed sufficient to determine whether a player was a center both on offense and defense.

Finally, with a rough idea of the required playing time for classification to become reliable, the following approach is proposed for assigning unusable players a position: let *n*^(*i*)^ denote the number of possessions played by player *i*, let *ℓ*_*k*_ denote their position, let $c^{\left(\ell_{k}\right)}$ denote the mean cluster membership probabilities for non-center players with label *ℓ*_*k*_, and let **p**^(*i*)^ denote the predicted probabilities for directly classifying player *i*’s feature vector. In the spirit of shrinkage estimation (Efron and Morris 1973), a “smoothed” probability, $p_{S}^{\left(i\right)}$ , is computed first, viz.

$$p_{S}^{\left(i\right)}=\left(\frac{n^{\left(i\right)}}{\text{1,000}}\right)\times p^{\left(i\right)}+\left(\frac{\text{1,000}-n^{\left(i\right)}}{\text{1,000}}\right)\times c^{\left(\ell_{k}\right)}.$$

The position with the greatest value within the $p_{S}^{\left(i\right)}$ vector is then assigned.

A similar formula was used to smooth center probabilities, but the denominator was set to 500 instead. Unusable players were classified as centers if their smoothed center probability was larger than 0.5. Otherwise, they were classified according to their most probable non-center position (recall that these probabilities are conditional on the fact that the player is not a center).

A full table containing the position assignments of all unusable players can be found in Part F of the Online Supplementary. Note that although six underlying offensive positions and five underlying defensive positions were estimated, no unusable players were assigned to offensive Position 4, and no players were assigned to defensive Position 2, which means in the context of rebounding analysis, there were effectively five offensive replacement players and four defensive replacement players.
