# Supplementary material for: A Bayesian two-stage framework for lineup-independent assessment of individual rebounding ability in the NBA
Source: J Quant Anal Sports. 2024 Dec 25;21(4):303–26. doi: 10.1515/jqas-2023-0097 (PMC12671482; doi:10.1515/jqas-2023-0097)
Supplement: Supplementary file 1 — Supplementary Material Details [file j_jqas-2023-0097_suppl_001.pdf]

N. Kiriazis, C. Genest*, and A. Leblanc

Online Supplement to the paper

“A Bayesian two-stage framework for lineup-independent assessment of individual rebounding ability in the NBA

Part A

Variables that were indicative of play style were favored over those that were indicative of effectiveness; for example, 3PT shots attempted were used rather than 3PT shots made.

A glossary giving the definition of most statistics can be found at

https://www.nba.com/stats/help/glossary.

Note that some of the tracking data are not actually included in the glossary, but they generally have quite descriptive names. Some examples of such data can be found at

https://www.nba.com/stats/players/drives.

Defense variables

AVG DREB Distance	DEF Loose Balls Recovered	STL
Avg Speed Def	DFGA	matchup3FGA
Contested 2PT Shots	Dist. Miles Def	matchupTurnovers
Contested 3PT Shots	DREB Chances	matchup2FGA
Deflections	Uncontested DREB	
DEF Boxouts	PFD	

Offense variables

AVG DRIB PER TOUCH	CATCH SHOOT FG2A	DRIVE AST
AVG OREB Distance	CATCH SHOOT FG3A	DRIVE FGA
AVG SEC PER TOUCH	CFGA	DRIVE FTA
Avg Speed Off	DIST MILES OFF	DRIVE PASS NO AST

N. Kiriazis, Department of Mathematics and Statistics, McGill University, Montréal (Québec) Canada H3A 0B9 e-mail: nicholas.kiriazis@mail.mcgill.ca
***Corresponding author: C. Genest,** Department of Mathematics and Statistics, McGill University, Montréal (Québec) Canada H3A 0B9 e-mail: christian.genest@mcgill.ca
A. Leblanc, Department of Statistics, University of Manitoba, Winnipeg, Manitoba, Canada R3T 2N2 e-mail: Alex.Leblanc@umanitoba.ca

DRIVE TOV
ELBOW TOUCH AST
ELBOW TOUCH FGA
ELBOW TOUCH FTA
ELBOW TOUCH PASS NO AST
ELBOW TOUCH TOV
FT AST
OFF BOXOUTS
OFF LOOSE BALLS RECOVERED
OREB CHANCES
PAINT TOUCH AST
PAINT TOUCH FGA
PAINT TOUCH FTA
PAINT TOUCH PASS NO AST
PAINT TOUCH TOV
PASSES RECEIVED
PERIMETER AST
PERIMETER FTA
PERIMETER TOUCHES
POST TOUCH AST
POST TOUCH FGA
POST TOUCH FTA
POST TOUCH PASS NO AST
POST TOUCH TOV
POTENTIAL AST
PULL UP FG2A
PULL UP FG3A
SCREEN ASSISTS
SECONDARY AST
UFGA

Part B

See pages 3–13.

	Off. β			Def. β			Off. γ		Def. γ		Discrepancy	
Name	Pr. Mean	Post. Mean	Post. SD	Pr. Mean	Post. Mean	Post. SD	Post. Mean	Post. SD	Post. Mean	Post. SD	Off.	Def.
Achiuwa, Precious	0.10	0.092	0.07	0.17	0.139	0.07	0.327	0.16	1.463	0.11	42.0	84.0
Adams, Steven	0.13	0.231	0.07	0.19	0.302	0.07	0.694	0.10	1.498	0.10	−3.0	−61.0
Adebayo, Bam	0.07	0.027	0.07	0.20	0.218	0.06	0.036	0.11	1.647	0.08	130.0	18.0
Aldridge, LaMarcus	0.03	−0.108	0.07	0.15	0.256	0.07	−0.868	0.26	1.154	0.12	179.0	−142.0
Alexander-Walker, Nickeil	0.01	0.073	0.06	0.13	0.069	0.06	−1.828	0.29	1.206	0.11	−230.0	134.0
Allen, Grayson	0.01	−0.039	0.06	0.11	0.113	0.06	−1.525	0.25	1.161	0.11	5.0	47.0
Allen, Jarrett	0.11	0.204	0.06	0.23	0.205	0.06	0.435	0.10	1.613	0.07	−11.0	29.0
Anderson, Kyle	0.03	−0.064	0.06	0.17	0.181	0.06	−0.902	0.15	1.669	0.09	148.0	66.0
Antetokounmpo, Giannis	0.05	0.007	0.06	0.26	0.285	0.06	−0.212	0.12	2.137	0.08	147.0	13.0
Antetokounmpo, Thanasis	0.09	0.163	0.07	0.11	0.052	0.07	0.383	0.17	1.124	0.14	−2.0	127.0
Anthony, Carmelo	0.02	0.011	0.06	0.11	0.035	0.06	−1.472	0.19	1.001	0.10	−64.0	99.5
Anthony, Cole	0.03	0.095	0.06	0.14	0.104	0.06	−0.902	0.18	1.283	0.09	−107.0	96.5
Anunoby, O.G.	0.04	−0.083	0.06	0.12	0.085	0.06	−0.512	0.16	0.993	0.09	222.0	37.0
Ariza, Trevor	0.03	−0.031	0.07	0.15	0.178	0.07	−0.586	0.22	1.317	0.12	158.0	−18.0
Augustin, D.J.	0.02	0.009	0.06	0.06	0.069	0.06	−1.345	0.23	0.534	0.13	−39.0	−51.0
Avdija, Deni	0.02	−0.063	0.06	0.18	0.202	0.06	−1.239	0.23	1.433	0.09	83.0	−10.0
Ayton, Deandre	0.12	0.195	0.07	0.24	0.234	0.07	0.781	0.11	1.925	0.08	13.0	30.0
Bacon, Dwayne	0.01	0.003	0.06	0.10	0.156	0.06	−1.413	0.19	0.892	0.09	−40.0	−126.0
Bagley, Marvin	0.09	0.170	0.07	0.19	0.169	0.06	0.412	0.13	1.576	0.10	1.0	66.5
Ball, LaMelo	0.04	0.086	0.06	0.16	0.119	0.06	−0.553	0.15	1.306	0.10	−38.0	79.0
Ball, Lonzo	0.02	0.090	0.06	0.13	0.148	0.06	−1.320	0.19	1.175	0.10	−179.0	−12.0
Bamba, Mo	0.09	0.127	0.07	0.24	0.201	0.07	0.399	0.15	1.842	0.10	20.0	62.0
Bane, Desmond	0.02	0.103	0.06	0.12	0.134	0.06	−1.357	0.19	1.248	0.10	−200.0	26.0
Barnes, Harrison	0.03	−0.009	0.07	0.15	0.153	0.06	−0.708	0.14	1.332	0.08	106.0	26.0
Barrett, RJ	0.03	0.107	0.07	0.13	0.063	0.07	−0.936	0.14	1.174	0.09	−125.0	135.0
Barton, Will	0.02	0.060	0.06	0.11	0.070	0.06	−1.189	0.18	1.040	0.10	−118.0	75.0
Batum, Nicolas	0.03	0.008	0.06	0.14	0.059	0.06	−0.908	0.16	1.383	0.09	47.0	207.0
Baynes, Aron	0.08	0.123	0.07	0.19	0.208	0.07	0.242	0.13	1.438	0.10	6.0	−17.0
Bazemore, Kent	0.02	−0.015	0.06	0.14	0.123	0.06	−1.229	0.21	1.112	0.10	24.0	6.0
Bazley, Darius	0.03	−0.043	0.06	0.19	0.241	0.06	−0.827	0.15	1.592	0.08	144.0	−9.0
Beal, Bradley	0.03	0.142	0.06	0.09	0.054	0.06	−0.628	0.14	0.731	0.09	−104.0	5.0
Beasley, Malik	0.02	0.032	0.06	0.11	0.076	0.06	−1.293	0.21	0.961	0.12	−77.0	37.0
Bembry, DeAndre'	0.03	0.050	0.06	0.11	0.059	0.07	−0.727	0.19	0.918	0.11	−12.0	49.0
Bertans, Davis	0.01	0.058	0.06	0.10	0.089	0.06	−1.775	0.25	0.759	0.10	−202.0	−36.0
Beverley, Patrick	0.04	0.017	0.07	0.11	0.126	0.07	−0.558	0.21	1.088	0.13	85.0	−4.0
Bey, Saddiq	0.02	−0.050	0.06	0.14	0.122	0.06	−1.114	0.17	1.242	0.08	96.0	51.0

	Off. β			Def. β			Off. γ		Def. γ		Discrepancy	
Name	Pr. Mean	Post. Mean	Post. SD	Pr. Mean	Post. Mean	Post. SD	Post. Mean	Post. SD	Post. Mean	Post. SD	Off.	Def.
Birch, Khem	0.10	0.178	0.06	0.14	0.172	0.06	0.560	0.10	1.120	0.08	10.0	−70.0
Bitadze, Goga	0.10	0.228	0.07	0.15	0.169	0.07	0.188	0.16	1.113	0.13	−45.0	−68.5
Biyombo, Bismack	0.09	0.158	0.06	0.15	0.221	0.06	0.335	0.12	1.125	0.10	−5.0	−128.0
Bjelica, Nemanja	0.05	−0.001	0.07	0.17	0.187	0.07	−0.183	0.22	1.450	0.13	167.5	12.0
Bledsoe, Eric	0.01	0.094	0.06	0.10	0.106	0.06	−1.633	0.20	0.915	0.10	−236.0	−30.0
Bogdanovic, Bogdan	0.02	−0.067	0.06	0.10	0.184	0.06	−1.369	0.24	0.917	0.11	59.0	−158.0
Bogdanovic, Bojan	0.02	0.001	0.07	0.10	0.078	0.07	−1.325	0.17	0.911	0.09	−22.0	20.0
Booker, Devin	0.02	0.085	0.07	0.11	0.016	0.07	−1.310	0.19	1.125	0.09	−173.0	168.0
Boucher, Chris	0.08	0.110	0.06	0.19	0.041	0.06	0.155	0.12	1.499	0.08	6.5	258.0
Bradley, Avery	0.01	−0.085	0.07	0.08	0.022	0.07	−1.590	0.38	0.654	0.16	39.0	21.0
Bradley, Tony	0.12	0.180	0.07	0.22	0.248	0.07	0.697	0.15	1.707	0.11	15.0	4.0
Bridges, Mikal	0.04	0.034	0.07	0.10	0.062	0.07	−0.468	0.14	1.021	0.09	61.0	81.5
Bridges, Miles	0.04	0.076	0.06	0.16	0.109	0.06	−0.629	0.14	1.229	0.09	−42.0	71.0
Brissett, Oshae	0.05	0.043	0.07	0.15	0.136	0.07	−0.087	0.22	1.248	0.14	86.0	19.0
Brogdon, Malcolm	0.03	0.112	0.06	0.12	0.134	0.06	−0.814	0.15	1.062	0.09	−112.0	−31.0
Brooks, Armoni	0.02	−0.042	0.07	0.11	0.071	0.07	−1.321	0.34	0.980	0.15	43.5	47.0
Brooks, Dillon	0.03	−0.011	0.06	0.07	0.066	0.06	−0.904	0.15	0.716	0.11	78.0	−12.5
Brown, Bruce	0.08	0.143	0.06	0.15	0.077	0.06	0.261	0.12	1.230	0.08	−6.0	132.0
Brown, Jaylen	0.04	0.050	0.06	0.14	0.128	0.06	−0.728	0.13	1.253	0.08	−15.0	41.0
Brown, Moses	0.14	0.257	0.07	0.24	0.265	0.07	0.942	0.12	1.860	0.10	2.0	7.0
Brown, Sterling	0.03	−0.022	0.06	0.15	0.106	0.06	−0.816	0.18	1.256	0.09	111.5	85.0
Brown, Troy	0.04	0.068	0.07	0.16	0.098	0.07	−0.611	0.26	1.343	0.13	−28.5	132.0
Brunson, Jalen	0.02	0.013	0.06	0.12	0.205	0.06	−1.536	0.21	0.861	0.09	−75.0	−202.0
Bullock, Reggie	0.01	−0.105	0.06	0.10	0.168	0.06	−2.036	0.26	0.923	0.10	6.0	−127.0
Burke, Trey	0.01	0.048	0.07	0.05	−0.020	0.06	−1.994	0.34	−0.049	0.16	−198.0	−3.0
Burks, Alec	0.02	0.013	0.07	0.16	0.142	0.06	−1.510	0.24	1.345	0.10	−72.0	46.5
Butler, Jimmy	0.06	0.049	0.06	0.15	0.217	0.06	−0.132	0.13	1.362	0.09	68.0	−50.5
Caldwell-Pope, Kentavious	0.01	−0.048	0.06	0.08	0.068	0.06	−1.573	0.20	0.677	0.11	13.0	−27.0
Campazzo, Facundo	0.01	−0.001	0.06	0.08	0.172	0.06	−1.556	0.22	0.676	0.11	−53.0	−204.0
Capela, Clint	0.15	0.212	0.06	0.30	0.343	0.06	1.040	0.10	2.101	0.08	10.0	1.0
Carter, Jevon	0.02	−0.055	0.07	0.11	0.141	0.07	−1.036	0.27	1.000	0.14	116.0	−74.0
Carter, Wendell	0.09	0.144	0.06	0.22	0.202	0.06	0.341	0.12	1.642	0.08	3.0	39.0
Carter-Williams, Michael	0.04	−0.042	0.07	0.12	0.180	0.07	−0.091	0.18	1.056	0.12	238.5	−104.0
Caruso, Alex	0.03	0.100	0.06	0.11	0.132	0.06	−1.105	0.20	0.987	0.11	−146.0	−59.0
Cauley-Stein, Willie	0.07	−0.041	0.07	0.17	0.216	0.07	0.169	0.16	1.321	0.10	256.0	−62.0
Clark, Gary	0.04	0.090	0.07	0.12	0.194	0.07	−0.395	0.19	1.051	0.13	−26.0	−119.0

	Off. β			Def. β			Off. γ		Def. γ		Discrepancy	
Name	Pr. Mean	Post. Mean	Post. SD	Pr. Mean	Post. Mean	Post. SD	Post. Mean	Post. SD	Post. Mean	Post. SD	Off.	Def.
Clarke, Brandon	0.06	0.053	0.06	0.16	0.151	0.06	−0.007	0.12	1.576	0.10	76.0	93.0
Clarkson, Jordan	0.03	0.050	0.06	0.12	0.120	0.06	−1.034	0.16	1.060	0.10	−65.0	−3.0
Claxton, Nic	0.07	0.108	0.07	0.19	0.219	0.07	0.003	0.19	1.324	0.11	5.0	−66.0
Collins, John	0.06	0.166	0.06	0.18	0.182	0.06	0.005	0.12	1.558	0.09	−32.0	38.0
Conley, Mike	0.02	0.039	0.06	0.08	0.123	0.06	−1.105	0.18	0.728	0.11	−60.0	−108.0
Connaughton, Pat	0.04	0.076	0.06	0.15	0.166	0.06	−0.507	0.14	1.506	0.09	−18.0	59.0
Cousins, DeMarcus	0.07	0.090	0.07	0.29	0.331	0.07	0.049	0.17	2.085	0.10	28.0	2.0
Covington, Robert	0.03	0.061	0.06	0.18	0.229	0.06	−1.013	0.14	1.591	0.08	−86.0	−4.0
Craig, Torrey	0.07	0.068	0.07	0.18	0.073	0.07	0.157	0.16	1.732	0.11	60.0	260.0
Crowder, Jae	0.02	−0.139	0.06	0.15	0.172	0.06	−0.992	0.20	1.383	0.09	159.0	15.0
Culver, Jarrett	0.06	0.049	0.07	0.13	0.063	0.07	−0.175	0.20	1.151	0.15	63.5	128.5
Curry, Seth	0.01	−0.023	0.07	0.07	0.095	0.07	−2.326	0.33	0.729	0.12	−75.0	−52.0
Curry, Stephen	0.01	0.106	0.07	0.14	0.072	0.07	−1.584	0.20	1.134	0.09	−237.5	112.0
Davis, Anthony	0.05	0.139	0.06	0.20	0.265	0.07	−0.326	0.15	1.616	0.10	−61.0	−19.0
Davis, Terence	0.02	0.015	0.06	0.12	0.110	0.06	−1.395	0.23	1.077	0.10	−60.5	22.0
DeRozan, DeMar	0.02	0.022	0.06	0.11	0.129	0.06	−1.200	0.17	0.920	0.09	−43.0	−71.0
DiVincenzo, Donte	0.04	0.033	0.06	0.15	0.104	0.06	−0.323	0.13	1.584	0.09	76.0	179.5
Diallo, Hamidou	0.05	0.067	0.06	0.16	0.109	0.06	−0.387	0.15	1.381	0.09	6.0	122.0
Dieng, Gorgui	0.07	0.110	0.07	0.18	0.131	0.07	−0.124	0.19	1.645	0.13	−18.5	141.0
Doncic, Luka	0.02	0.055	0.06	0.20	0.119	0.06	−1.079	0.15	1.528	0.08	−84.0	138.0
Dort, Luguentz	0.02	−0.008	0.06	0.09	0.082	0.07	−1.018	0.18	0.813	0.11	52.0	−13.0
Dotson, Damyean	0.01	0.030	0.06	0.10	0.060	0.07	−2.763	0.47	0.836	0.12	−177.0	23.0
Doumbouya, Sekou	0.04	0.019	0.07	0.12	0.015	0.07	−0.346	0.19	1.015	0.11	101.5	125.0
Dozier, PJ	0.03	0.126	0.06	0.13	0.206	0.06	−0.963	0.18	1.167	0.10	−150.0	−95.0
Dragic, Goran	0.02	−0.024	0.06	0.11	0.135	0.06	−1.301	0.22	0.976	0.10	27.0	−74.0
Drummond, Andre	0.13	0.209	0.06	0.34	0.317	0.07	0.841	0.11	2.252	0.08	9.0	8.0
Durant, Kevin	0.01	−0.009	0.07	0.18	0.198	0.06	−1.775	0.30	1.368	0.09	−70.0	−23.0
Edwards, Anthony	0.02	−0.025	0.06	0.12	0.109	0.06	−1.156	0.15	1.067	0.10	55.0	21.0
Ellington, Wayne	0.01	−0.021	0.07	0.07	0.052	0.07	−1.764	0.30	0.538	0.13	−49.0	−33.0
Embiid, Joel	0.08	0.072	0.07	0.25	0.240	0.07	0.168	0.12	1.977	0.09	57.0	31.0
Ennis, James	0.04	−0.001	0.06	0.12	0.111	0.06	−0.248	0.18	1.117	0.10	158.5	29.0
Eubanks, Drew	0.09	0.110	0.07	0.21	0.206	0.07	0.298	0.15	1.671	0.11	20.0	37.0
Favors, Derrick	0.13	0.190	0.07	0.23	0.307	0.08	0.667	0.12	1.673	0.10	10.0	−26.0
Finney-Smith, Dorian	0.05	0.068	0.06	0.12	0.177	0.06	−0.292	0.12	0.888	0.09	15.5	−156.0
Flynn, Malachi	0.01	−0.014	0.07	0.11	0.217	0.07	−1.899	0.34	0.872	0.11	−74.0	−213.0
Forbes, Bryn	0.01	−0.037	0.06	0.07	0.062	0.06	−2.117	0.31	0.680	0.12	−51.0	−17.5

	Off. β			Def. β			Off. γ		Def. γ		Discrepancy	
Name	Pr. Mean	Post. Mean	Post. SD	Pr. Mean	Post. Mean	Post. SD	Post. Mean	Post. SD	Post. Mean	Post. SD	Off.	Def.
Fournier, Evan	0.01	−0.042	0.06	0.09	0.099	0.06	−2.207	0.34	0.824	0.10	−46.5	−39.0
Fox, De'Aaron	0.02	−0.055	0.06	0.09	0.078	0.06	−1.323	0.18	0.730	0.10	57.0	−24.0
Freedom, Enes	0.15	0.330	0.06	0.28	0.334	0.06	0.785	0.10	2.051	0.08	−3.0	0.0
Gafford, Daniel	0.12	0.223	0.07	0.16	0.069	0.06	0.613	0.13	1.327	0.10	−4.0	172.0
Gallinari, Danilo	0.01	−0.124	0.06	0.15	0.179	0.06	−1.490	0.26	1.312	0.10	64.0	−22.0
Garland, Darius	0.01	0.027	0.06	0.06	0.036	0.06	−1.665	0.22	0.272	0.11	−129.0	−28.0
Gasol, Marc	0.04	−0.047	0.07	0.17	0.227	0.07	−0.632	0.18	1.399	0.11	172.0	−49.0
Gay, Rudy	0.03	0.005	0.07	0.18	0.149	0.06	−0.854	0.17	1.468	0.09	65.0	74.0
George, Paul	0.03	0.022	0.06	0.17	0.221	0.06	−0.980	0.17	1.543	0.09	−5.5	−14.0
Gibson, Taj	0.10	0.140	0.07	0.15	0.205	0.07	0.390	0.14	1.217	0.11	10.0	−79.0
Gilgeous-Alexander, Shai	0.01	−0.039	0.07	0.12	0.095	0.07	−1.451	0.24	1.075	0.11	15.0	53.5
Gobert, Rudy	0.11	0.154	0.07	0.29	0.284	0.07	0.452	0.10	2.008	0.08	13.0	10.0
Goodwin, Brandon	0.01	0.047	0.07	0.10	0.134	0.07	−1.797	0.39	0.823	0.15	−182.0	−110.0
Gordon, Aaron	0.06	0.137	0.06	0.15	0.146	0.06	−0.126	0.13	1.362	0.09	−35.0	45.5
Gordon, Eric	0.01	0.039	0.07	0.06	0.025	0.07	−2.077	0.37	0.352	0.16	−188.0	−11.0
Graham, Devonte'	0.01	0.061	0.06	0.07	0.091	0.06	−1.761	0.23	0.440	0.12	−211.0	−89.5
Grant, Jerami	0.02	0.081	0.06	0.12	0.152	0.06	−1.335	0.18	1.051	0.09	−174.0	−65.0
Green, Danny	0.03	0.052	0.07	0.10	0.029	0.07	−0.776	0.15	1.040	0.10	−23.0	126.0
Green, Draymond	0.03	−0.042	0.07	0.18	0.211	0.07	−0.786	0.15	1.432	0.09	143.0	−20.0
Green, JaMychal	0.07	0.131	0.06	0.18	0.190	0.07	−0.075	0.13	1.467	0.10	−24.0	15.0
Green, Jeff	0.02	−0.080	0.06	0.12	0.107	0.06	−1.274	0.18	0.868	0.09	88.0	−45.0
Griffin, Blake	0.03	0.045	0.06	0.16	0.196	0.06	−0.918	0.19	1.265	0.09	−36.0	−55.0
Hachimura, Rui	0.03	−0.104	0.06	0.14	0.230	0.06	−0.764	0.17	1.147	0.08	192.0	−127.0
Haliburton, Tyrese	0.02	0.070	0.06	0.08	0.003	0.06	−1.111	0.18	0.624	0.11	−114.0	21.0
Hampton, R.J.	0.03	−0.050	0.07	0.16	0.006	0.07	−0.741	0.20	1.479	0.10	161.0	279.0
Hardaway, Tim	0.01	−0.042	0.06	0.10	0.018	0.06	−1.958	0.24	0.771	0.09	−35.5	50.0
Harden, James	0.02	0.032	0.06	0.19	0.159	0.06	−1.075	0.18	1.468	0.08	−39.5	57.0
Harkless, Maurice	0.02	−0.079	0.07	0.10	0.097	0.07	−1.239	0.29	0.855	0.13	93.0	−31.0
Harrell, Montrezl	0.10	0.126	0.07	0.17	0.125	0.07	0.384	0.11	1.356	0.09	17.0	84.0
Harris, Gary	0.02	0.015	0.06	0.05	0.097	0.06	−1.343	0.24	0.238	0.15	−51.0	−111.0
Harris, Joe	0.02	−0.014	0.06	0.09	0.178	0.06	−1.170	0.16	0.633	0.09	30.0	−223.0
Harris, Tobias	0.03	−0.026	0.06	0.17	0.234	0.07	−0.706	0.14	1.565	0.09	138.0	−15.0
Hart, Josh	0.04	0.046	0.06	0.23	0.254	0.07	−0.594	0.16	1.840	0.09	13.0	11.0
Hartenstein, Isaiah	0.11	0.188	0.07	0.21	0.173	0.07	0.416	0.16	1.578	0.12	−6.0	59.5
Hayes, Jaxson	0.09	0.108	0.07	0.16	0.193	0.07	0.129	0.13	1.376	0.11	11.0	−13.0
Hayes, Killian	0.01	−0.034	0.07	0.09	0.172	0.07	−2.107	0.44	0.728	0.15	−51.0	−191.0

Name	Off. β			Def. β			Off. γ		Def. γ		Discrepancy	
	Pr. Mean	Post. Mean	Post. SD	Pr. Mean	Post. Mean	Post. SD	Post. Mean	Post. SD	Post. Mean	Post. SD	Off.	Def.
Hayward, Gordon	0.02	−0.048	0.06	0.14	0.145	0.06	−1.058	0.18	1.125	0.10	107.0	−23.0
Hernangomez, Juancho	0.05	0.086	0.06	0.18	0.186	0.07	−0.494	0.17	1.459	0.11	−29.0	16.0
Hernangomez, Willy	0.13	0.130	0.07	0.25	0.195	0.07	0.813	0.13	1.963	0.10	46.0	75.0
Herro, Tyler	0.01	0.060	0.06	0.14	0.039	0.06	−1.580	0.22	1.333	0.09	−181.0	214.0
Hield, Buddy	0.01	0.042	0.06	0.13	0.118	0.06	−1.656	0.20	1.151	0.08	−159.0	37.5
Hill, George	0.02	0.015	0.07	0.06	−0.014	0.07	−1.128	0.27	0.434	0.17	−10.0	10.0
Hill, Solomon	0.03	0.011	0.06	0.11	0.100	0.06	−0.841	0.18	0.965	0.10	58.0	−1.0
Holiday, Aaron	0.01	0.017	0.06	0.06	−0.000	0.06	−1.795	0.27	0.276	0.13	−120.0	−3.0
Holiday, Jrue	0.04	0.073	0.06	0.09	0.176	0.06	−0.499	0.14	1.001	0.10	−14.0	−121.5
Holiday, Justin	0.01	−0.095	0.06	0.10	0.090	0.06	−1.469	0.19	0.840	0.09	61.0	−19.0
Holmes, Richaun	0.08	0.058	0.06	0.20	0.178	0.06	0.338	0.11	1.592	0.08	95.0	58.5
Hood, Rodney	0.02	−0.043	0.06	0.08	0.046	0.06	−1.155	0.23	0.687	0.13	81.0	3.0
Horford, Al	0.03	−0.079	0.07	0.20	0.288	0.07	−0.567	0.22	1.559	0.11	208.0	−48.0
Horton-Tucker, Talen	0.02	0.001	0.06	0.10	0.046	0.06	−1.281	0.22	0.890	0.10	−9.0	52.0
House, Danuel	0.02	−0.082	0.07	0.12	0.101	0.06	−1.285	0.25	1.049	0.11	88.0	28.0
Howard, Dwight	0.15	0.263	0.07	0.31	0.213	0.07	0.651	0.11	2.159	0.10	−9.0	63.0
Huerter, Kevin	0.02	0.005	0.06	0.09	0.120	0.06	−1.268	0.17	0.798	0.10	−9.0	−87.0
Hunter, De'Andre	0.02	0.023	0.07	0.12	0.152	0.07	−1.006	0.27	1.144	0.13	−12.0	−27.0
Ibaka, Serge	0.08	0.037	0.07	0.22	0.276	0.07	0.229	0.16	1.800	0.11	120.0	−3.0
Iguodala, Andre	0.03	0.092	0.06	0.13	0.089	0.06	−0.964	0.18	1.211	0.10	−119.0	108.0
Ingles, Joe	0.01	0.021	0.06	0.11	0.142	0.06	−1.607	0.21	0.991	0.10	−113.0	−77.5
Ingram, Brandon	0.02	−0.051	0.06	0.13	0.155	0.07	−1.393	0.18	1.141	0.09	44.0	−35.0
Irving, Kyrie	0.03	0.032	0.06	0.10	0.009	0.06	−0.869	0.16	0.775	0.09	0.5	55.0
Iwundu, Wes	0.03	0.022	0.07	0.13	0.090	0.07	−1.073	0.28	1.125	0.14	−25.0	80.5
Jackson, Frank	0.02	−0.016	0.07	0.09	0.135	0.07	−1.137	0.28	0.704	0.14	40.0	−142.0
Jackson, Josh	0.04	0.123	0.06	0.12	0.095	0.06	−0.694	0.15	1.065	0.09	−98.0	48.5
Jackson, Justin	0.03	0.019	0.07	0.10	0.152	0.07	−0.959	0.27	0.862	0.15	8.0	−126.5
Jackson, Reggie	0.02	−0.051	0.06	0.11	0.215	0.06	−1.421	0.22	0.984	0.11	35.0	−178.5
James, LeBron	0.02	0.014	0.06	0.20	0.195	0.06	−1.322	0.20	1.616	0.09	−45.0	45.0
Jeffries, DaQuan	0.04	0.036	0.07	0.10	0.163	0.07	−0.541	0.25	0.855	0.15	46.0	−146.5
Jerome, Ty	0.01	0.047	0.07	0.10	0.097	0.07	−1.730	0.34	0.839	0.13	−173.0	−31.0
Johnson, Cameron	0.02	0.054	0.06	0.12	0.151	0.06	−1.049	0.20	1.127	0.10	−74.0	−27.0
Johnson, James	0.03	−0.067	0.06	0.13	0.074	0.06	−0.813	0.20	1.122	0.10	167.0	101.0
Johnson, Keldon	0.04	0.021	0.06	0.16	0.116	0.06	−0.262	0.13	1.381	0.09	105.0	108.0
Johnson, Stanley	0.03	−0.022	0.06	0.13	0.145	0.06	−0.906	0.20	1.014	0.11	95.0	−68.0
Johnson, Tyler	0.01	−0.011	0.07	0.10	0.088	0.07	−2.205	0.43	0.643	0.13	−92.0	−61.0

	Off. β			Def. β			Off. γ		Def. γ		Discrepancy	
Name	Pr. Mean	Post. Mean	Post. SD	Pr. Mean	Post. Mean	Post. SD	Post. Mean	Post. SD	Post. Mean	Post. SD	Off.	Def.
Jokic, Nikola	0.09	0.096	0.06	0.24	0.300	0.06	0.202	0.10	1.823	0.07	29.0	−10.0
Jones, Damian	0.06	0.022	0.07	0.16	0.159	0.07	0.029	0.20	1.269	0.13	135.5	−4.0
Jones, Derrick	0.06	−0.071	0.06	0.09	0.044	0.06	−0.075	0.14	0.918	0.11	268.0	68.0
Jones, Tyus	0.02	−0.031	0.06	0.09	0.003	0.07	−1.419	0.22	1.048	0.12	11.0	141.0
Jordan, DeAndre	0.10	0.089	0.07	0.23	0.283	0.06	0.543	0.13	1.649	0.08	72.0	−19.0
Joseph, Cory	0.02	0.021	0.06	0.09	0.121	0.06	−1.020	0.18	0.691	0.10	−6.0	−115.0
Kaminsky, Frank	0.05	0.060	0.07	0.21	0.261	0.07	−0.237	0.20	1.732	0.11	34.0	−2.0
Kennard, Luke	0.01	0.007	0.06	0.12	0.046	0.06	−1.749	0.27	1.122	0.11	−89.0	134.0
Kleber, Maxi	0.04	0.017	0.06	0.15	0.255	0.06	−0.701	0.17	1.157	0.10	61.0	−139.0
Konchar, John	0.06	0.109	0.07	0.16	0.135	0.07	−0.226	0.20	1.589	0.13	−29.0	119.0
Korkmaz, Furkan	0.01	0.103	0.07	0.09	0.157	0.07	−1.710	0.26	0.814	0.13	−250.0	−150.0
Kuzma, Kyle	0.05	0.034	0.06	0.16	0.254	0.06	−0.207	0.12	1.316	0.09	87.0	−92.5
LaVine, Zach	0.02	−0.033	0.06	0.12	0.097	0.06	−1.269	0.18	1.013	0.08	48.0	22.0
Lamb, Jeremy	0.03	0.056	0.07	0.14	0.048	0.07	−0.844	0.22	1.192	0.12	−39.0	155.0
Layman, Jake	0.02	−0.031	0.07	0.08	0.111	0.07	−1.379	0.30	0.631	0.16	17.0	−114.0
LeVert, Caris	0.02	−0.043	0.06	0.11	−0.021	0.06	−1.031	0.19	0.982	0.09	105.0	121.0
Lee, Damion	0.02	−0.069	0.07	0.14	0.139	0.06	−1.148	0.23	1.041	0.11	105.0	−50.0
Lee, Saben	0.03	−0.122	0.07	0.10	0.090	0.07	−0.778	0.23	0.810	0.13	194.0	−27.5
Len, Alex	0.08	0.044	0.07	0.17	0.256	0.07	0.347	0.14	1.395	0.10	123.0	−70.0
Leonard, Kawhi	0.03	0.039	0.06	0.16	0.211	0.06	−0.710	0.15	1.483	0.09	7.0	−5.0
Lewis, Kira	0.01	0.104	0.07	0.06	0.158	0.07	−1.869	0.31	0.436	0.15	−267.0	−213.0
Lillard, Damian	0.01	0.060	0.06	0.10	0.102	0.06	−1.746	0.19	1.029	0.09	−203.0	19.0
Little, Nassir	0.05	0.056	0.07	0.14	0.116	0.07	−0.441	0.20	1.325	0.13	20.0	89.0
Looney, Kevon	0.10	0.202	0.07	0.17	0.127	0.06	0.436	0.13	1.293	0.10	−8.0	56.0
Lopez, Brook	0.05	0.104	0.06	0.12	0.207	0.06	−0.118	0.12	1.200	0.09	−5.0	−89.0
Lopez, Robin	0.10	0.169	0.06	0.09	0.200	0.06	0.446	0.12	0.651	0.10	7.0	−243.0
Love, Kevin	0.03	−0.009	0.07	0.27	0.348	0.07	−0.945	0.26	1.824	0.10	65.0	−17.0
Lowry, Kyle	0.02	−0.062	0.06	0.13	0.090	0.06	−1.129	0.18	1.074	0.09	102.0	64.0
Luwawu-Cabarrot, Timothe	0.03	0.097	0.06	0.09	0.131	0.06	−1.058	0.21	0.618	0.11	−137.0	−153.0
Maledon, Theo	0.01	−0.041	0.06	0.10	0.100	0.06	−1.591	0.21	0.939	0.09	−3.0	−8.0
Mann, Terance	0.04	0.149	0.06	0.14	0.087	0.06	−0.552	0.15	1.313	0.10	−91.0	144.0
Markkanen, Lauri	0.03	−0.034	0.06	0.17	0.185	0.06	−0.942	0.19	1.382	0.09	105.0	−3.0
Marshall, Naji	0.02	0.072	0.07	0.18	0.223	0.07	−1.307	0.28	1.540	0.12	−155.0	−18.0
Martin, Caleb	0.04	0.035	0.07	0.12	0.019	0.06	−0.714	0.21	1.018	0.13	18.0	123.0
Martin, Cody	0.06	0.060	0.06	0.13	0.077	0.07	−0.115	0.17	1.003	0.13	48.0	50.0
Martin, Kenyon	0.06	0.091	0.06	0.16	0.168	0.07	−0.027	0.14	1.303	0.10	17.0	−5.0

Name	Off. β Pr. Mean	Off. β Post. Mean	Off. β Post. SD	Def. β Pr. Mean	Def. β Post. Mean	Def. β Post. SD	Off. γ Post. Mean	Off. γ Post. SD	Def. γ Post. Mean	Def. γ Post. SD	Discrepancy Off.	Discrepancy Def.
Mathews, Garrison	0.01	−0.084	0.06	0.07	0.033	0.06	−1.360	0.27	0.380	0.13	72.0	−17.0
Matthews, Wesley	0.02	0.024	0.06	0.06	0.088	0.06	−1.417	0.24	0.350	0.14	−94.0	−87.0
Maxey, Tyrese	0.01	0.026	0.06	0.10	0.068	0.07	−1.973	0.33	0.898	0.13	−156.0	33.0
McCollum, CJ	0.02	0.044	0.06	0.10	0.134	0.06	−1.405	0.19	0.960	0.10	−127.0	−71.5
McConnell, T.J.	0.03	0.075	0.06	0.11	0.045	0.06	−0.863	0.15	0.867	0.09	−73.0	49.0
McDaniels, Jaden	0.03	0.038	0.06	0.12	0.086	0.06	−0.901	0.15	1.012	0.11	−18.0	45.0
McDaniels, Jalen	0.05	0.001	0.06	0.14	0.094	0.07	−0.316	0.17	1.098	0.12	147.0	59.0
McDermott, Doug	0.04	−0.018	0.06	0.10	0.120	0.06	−0.558	0.15	0.767	0.10	145.0	−96.0
McGee, JaVale	0.10	0.207	0.07	0.23	0.178	0.07	0.331	0.14	1.785	0.10	−27.0	85.0
McLaughlin, Jordan	0.02	0.069	0.07	0.09	0.115	0.07	−1.398	0.23	0.700	0.13	−170.0	−102.0
McLemore, Ben	0.01	−0.010	0.06	0.09	0.051	0.06	−1.542	0.28	0.737	0.12	−39.5	9.0
Melli, Nicolo	0.05	0.140	0.07	0.17	0.188	0.07	−0.585	0.20	1.346	0.12	−96.0	−17.0
Melton, De'Anthony	0.03	0.077	0.06	0.12	0.114	0.06	−0.998	0.19	1.267	0.12	−102.0	74.0
Metu, Chimezie	0.06	0.078	0.07	0.17	0.163	0.07	−0.041	0.21	1.425	0.14	33.0	41.5
Middleton, Khris	0.02	−0.033	0.06	0.14	0.202	0.06	−0.938	0.16	1.464	0.09	104.0	−1.0
Mills, Patty	0.01	0.015	0.06	0.05	−0.004	0.06	−1.947	0.25	0.232	0.12	−123.5	−5.0
Millsap, Paul	0.07	0.109	0.06	0.17	0.161	0.07	−0.089	0.14	1.459	0.10	−8.5	50.0
Milton, Shake	0.02	0.061	0.06	0.08	0.041	0.07	−1.300	0.19	0.677	0.12	−139.0	9.0
Mitchell, Donovan	0.03	0.050	0.07	0.10	0.031	0.07	−0.897	0.16	0.878	0.10	−36.0	69.0
Monk, Malik	0.01	0.022	0.07	0.10	0.115	0.07	−1.622	0.30	0.675	0.14	−117.5	−112.0
Morant, Ja	0.03	0.115	0.06	0.09	0.032	0.07	−0.905	0.15	1.012	0.10	−131.0	108.0
Morris, Marcus	0.03	0.082	0.06	0.13	0.127	0.06	−1.133	0.19	1.251	0.10	−137.0	44.5
Morris, Markieff	0.04	0.082	0.06	0.18	0.171	0.06	−0.575	0.17	1.458	0.10	−40.0	36.0
Morris, Monte	0.01	0.056	0.06	0.07	0.057	0.06	−2.205	0.31	0.553	0.13	−223.0	−34.0
Mulder, Mychal	0.01	−0.080	0.07	0.06	0.044	0.07	−1.850	0.38	0.191	0.16	6.0	−37.0
Murray, Dejounte	0.03	0.011	0.06	0.19	0.215	0.06	−0.857	0.15	1.555	0.08	55.0	0.0
Murray, Jamal	0.02	0.115	0.06	0.10	0.049	0.06	−1.218	0.18	0.901	0.10	−188.0	54.5
Muscala, Mike	0.03	−0.043	0.07	0.16	0.243	0.07	−0.882	0.25	1.335	0.12	136.0	−76.0
Mykhailiuk, Svi	0.02	0.053	0.06	0.10	0.091	0.06	−1.405	0.22	0.923	0.10	−145.0	4.5
Nance, Larry	0.05	0.059	0.06	0.17	0.155	0.07	−0.246	0.16	1.405	0.10	34.0	46.0
Nesmith, Aaron	0.04	0.081	0.07	0.15	0.141	0.07	−0.583	0.22	1.281	0.12	−39.0	25.0
Neto, Raul	0.02	0.009	0.06	0.09	0.190	0.06	−1.179	0.20	0.598	0.11	−4.0	−242.0
Niang, Georges	0.02	0.030	0.07	0.12	0.164	0.07	−1.112	0.20	1.052	0.11	−42.0	−80.0
Noel, Nerlens	0.09	0.039	0.06	0.16	0.066	0.06	0.402	0.12	1.445	0.09	138.0	212.5
Nowell, Jaylen	0.02	−0.020	0.07	0.10	0.067	0.07	−1.256	0.25	0.797	0.15	29.0	4.0
Nunn, Kendrick	0.01	0.022	0.06	0.10	0.091	0.06	−1.690	0.24	0.860	0.10	−125.5	−17.5

	Off. β			Def. β			Off. γ		Def. γ		Discrepancy	
Name	Pr. Mean	Post. Mean	Post. SD	Pr. Mean	Post. Mean	Post. SD	Post. Mean	Post. SD	Post. Mean	Post. SD	Off.	Def.
Nurkic, Jusuf	0.09	0.005	0.07	0.28	0.358	0.08	0.394	0.15	2.027	0.10	214.0	−5.0
Nwaba, David	0.06	0.018	0.07	0.11	0.035	0.07	−0.074	0.19	0.930	0.13	143.0	83.5
O'Neale, Royce	0.04	0.082	0.07	0.16	0.277	0.07	−0.554	0.13	1.403	0.09	−37.0	−75.5
Ojeleye, Semi	0.03	0.019	0.06	0.12	0.178	0.07	−0.848	0.20	1.108	0.11	32.5	−87.5
Okeke, Chuma	0.03	−0.021	0.06	0.12	0.089	0.06	−0.511	0.17	1.025	0.10	157.0	47.0
Okogie, Josh	0.05	0.015	0.06	0.08	0.091	0.06	−0.363	0.14	0.568	0.13	112.0	−83.5
Okongwu, Onyeka	0.10	0.168	0.07	0.17	0.153	0.07	0.306	0.16	1.331	0.13	−12.0	26.0
Okoro, Isaac	0.03	−0.056	0.06	0.07	0.221	0.06	−0.714	0.14	0.336	0.10	171.0	−303.5
Oladipo, Victor	0.01	−0.162	0.07	0.14	0.121	0.06	−1.742	0.30	1.215	0.10	37.0	44.0
Olynyk, Kelly	0.04	0.061	0.06	0.20	0.216	0.06	−0.442	0.12	1.605	0.07	5.0	13.0
Oni, Miye	0.04	0.052	0.08	0.11	0.134	0.07	−0.503	0.23	0.894	0.15	21.0	−86.0
Osman, Cedi	0.02	0.051	0.06	0.11	0.027	0.06	−1.060	0.18	1.007	0.10	−73.0	111.0
Oubre, Kelly	0.05	−0.053	0.06	0.14	0.045	0.06	−0.187	0.13	1.109	0.09	237.0	129.0
Paschall, Eric	0.04	−0.092	0.08	0.14	0.127	0.07	−0.327	0.22	0.962	0.13	246.5	−55.5
Patterson, Patrick	0.03	0.020	0.07	0.09	0.102	0.07	−0.891	0.25	0.822	0.15	22.0	−47.0
Paul, Chris	0.01	−0.083	0.07	0.13	0.205	0.07	−1.520	0.21	1.302	0.09	49.0	−56.0
Payne, Cameron	0.02	0.012	0.07	0.12	0.128	0.07	−1.480	0.27	1.141	0.11	−66.0	11.5
Payton, Elfrid	0.04	0.151	0.07	0.10	0.101	0.07	−0.494	0.15	0.883	0.11	−85.0	−27.0
Plumlee, Mason	0.10	0.082	0.07	0.26	0.284	0.07	0.426	0.11	1.897	0.08	70.0	2.0
Poeltl, Jakob	0.11	0.173	0.07	0.17	0.275	0.06	0.690	0.10	1.417	0.09	18.0	−70.0
Pokusevski, Aleksej	0.03	−0.006	0.06	0.15	0.176	0.06	−0.928	0.20	1.403	0.10	64.5	17.0
Poole, Jordan	0.01	−0.010	0.07	0.07	0.105	0.06	−1.432	0.27	0.373	0.13	−26.5	−123.0
Porter, Kevin	0.02	0.064	0.07	0.10	0.084	0.07	−1.140	0.24	0.864	0.13	−115.0	1.0
Porter, Michael	0.05	0.014	0.06	0.19	0.200	0.06	−0.271	0.13	1.627	0.08	127.0	39.0
Porter, Otto	0.04	0.109	0.07	0.20	0.169	0.07	−0.454	0.21	1.589	0.12	−51.5	72.0
Portis, Bobby	0.09	0.179	0.06	0.23	0.192	0.06	0.308	0.12	1.980	0.09	−18.0	83.0
Porzingis, Kristaps	0.06	0.034	0.06	0.22	0.223	0.06	−0.155	0.14	1.576	0.09	94.0	−8.0
Powell, Dwight	0.09	0.034	0.07	0.16	0.119	0.07	0.283	0.15	1.225	0.11	131.0	55.0
Powell, Norman	0.02	0.005	0.05	0.08	0.087	0.05	−1.373	0.17	0.607	0.09	−35.0	−65.0
Prince, Taurean	0.02	−0.019	0.06	0.13	0.104	0.06	−1.160	0.24	1.111	0.11	40.0	42.0
Pritchard, Payton	0.03	0.090	0.06	0.10	0.112	0.06	−1.159	0.19	0.900	0.11	−152.0	−46.0
Quickley, Immanuel	0.02	0.080	0.07	0.09	0.076	0.06	−1.288	0.22	0.705	0.12	−158.0	−27.0
Randle, Chasson	0.01	−0.090	0.07	0.08	0.133	0.07	−2.075	0.39	0.704	0.13	−1.0	−135.0
Randle, Julius	0.03	−0.017	0.07	0.23	0.287	0.07	−0.750	0.13	1.726	0.08	111.0	−15.0
Reddish, Cam	0.03	0.028	0.07	0.11	0.147	0.07	−0.813	0.24	1.009	0.14	18.0	−77.0
Redick, JJ	0.01	−0.057	0.07	0.08	0.050	0.07	−2.579	0.54	0.717	0.15	−32.0	4.0

	Off. β			Def. β			Off. γ		Def. γ		Discrepancy	
Name	Pr. Mean	Post. Mean	Post. SD	Pr. Mean	Post. Mean	Post. SD	Post. Mean	Post. SD	Post. Mean	Post. SD	Off.	Def.
Reid, Naz	0.05	0.038	0.06	0.17	0.194	0.06	−0.304	0.13	1.370	0.10	66.0	−16.0
Richardson, Josh	0.03	0.069	0.06	0.08	0.127	0.06	−0.926	0.16	0.497	0.11	−81.0	−155.0
Rivers, Austin	0.01	0.020	0.07	0.09	0.044	0.07	−2.093	0.38	0.784	0.13	−148.0	30.0
Robinson, Duncan	0.00	−0.102	0.06	0.11	0.124	0.06	−3.201	0.43	1.020	0.09	−9.0	−26.0
Robinson, Mitchell	0.12	0.247	0.07	0.15	0.275	0.07	0.542	0.13	1.289	0.11	−15.0	−112.0
Roby, Isaiah	0.06	0.109	0.06	0.17	0.124	0.06	−0.108	0.13	1.500	0.09	−10.0	127.0
Rondo, Rajon	0.02	0.111	0.06	0.11	−0.012	0.07	−1.272	0.25	1.124	0.12	−195.0	177.0
Rose, Derrick	0.01	0.018	0.06	0.08	0.051	0.06	−1.550	0.24	0.643	0.11	−94.0	−17.0
Ross, Terrence	0.01	0.037	0.06	0.10	0.082	0.06	−1.871	0.27	0.921	0.10	−171.5	23.0
Rozier, Terry	0.02	0.029	0.06	0.10	0.092	0.06	−1.271	0.17	0.806	0.10	−67.0	−35.0
Rubio, Ricky	0.01	0.057	0.06	0.11	0.109	0.06	−1.684	0.20	0.984	0.11	−188.0	−15.5
Russell, D'Angelo	0.01	−0.002	0.06	0.08	0.042	0.06	−1.799	0.26	0.596	0.13	−81.0	−10.0
Sabonis, Domantas	0.07	0.013	0.06	0.26	0.288	0.06	0.092	0.11	1.877	0.07	169.0	−2.0
Saric, Dario	0.06	0.037	0.07	0.17	0.267	0.07	−0.210	0.18	1.420	0.11	81.0	−68.0
Satoransky, Tomas	0.02	0.018	0.06	0.09	0.128	0.06	−1.072	0.20	0.649	0.11	−9.0	−140.5
Schroder, Dennis	0.02	0.036	0.06	0.09	0.100	0.06	−1.525	0.20	0.788	0.10	−126.0	−52.0
Scott, Mike	0.01	−0.106	0.07	0.13	0.246	0.07	−1.780	0.33	1.114	0.13	24.0	−152.0
Sexton, Collin	0.03	0.121	0.06	0.06	0.049	0.06	−0.963	0.14	0.319	0.10	−145.0	−42.5
Shamet, Landry	0.01	−0.023	0.06	0.07	0.027	0.06	−2.205	0.31	0.294	0.11	−70.0	−17.0
Siakam, Pascal	0.04	0.082	0.06	0.16	0.158	0.06	−0.376	0.12	1.222	0.08	−13.0	−19.0
Simmons, Ben	0.05	−0.018	0.06	0.17	0.215	0.07	−0.187	0.12	1.554	0.09	189.0	−2.5
Simons, Anfernee	0.01	−0.018	0.06	0.11	0.030	0.06	−1.869	0.27	1.038	0.11	−66.0	123.0
Smart, Marcus	0.02	−0.005	0.06	0.08	0.154	0.06	−1.221	0.18	0.726	0.11	11.0	−166.0
Smith, Ish	0.02	0.010	0.07	0.13	0.105	0.07	−1.060	0.23	1.060	0.11	19.0	23.0
Snell, Tony	0.02	0.055	0.07	0.09	0.081	0.07	−1.311	0.26	0.830	0.13	−129.0	−5.0
Stevens, Lamar	0.04	0.081	0.07	0.14	0.061	0.07	−0.563	0.23	1.304	0.14	−33.0	175.0
Stewart, Isaiah	0.10	0.216	0.06	0.19	0.182	0.06	0.395	0.11	1.474	0.08	−22.0	27.0
Strus, Max	0.01	−0.017	0.08	0.07	0.134	0.08	−1.952	0.49	0.538	0.19	−72.0	−167.5
Sumner, Edmond	0.02	−0.026	0.07	0.08	−0.004	0.06	−1.176	0.26	0.639	0.13	53.0	28.0
Tate, Jae'Sean	0.06	0.144	0.05	0.12	0.163	0.06	−0.092	0.11	1.010	0.08	−38.0	−100.0
Tatum, Jayson	0.02	0.171	0.06	0.19	0.197	0.06	−1.333	0.16	1.597	0.08	−245.0	37.0
Teague, Jeff	0.02	0.002	0.06	0.07	−0.025	0.06	−1.529	0.26	0.693	0.13	−55.0	48.0
Temple, Garrett	0.02	−0.029	0.06	0.08	0.128	0.06	−1.235	0.19	0.582	0.10	48.0	−152.0
Theis, Daniel	0.06	0.061	0.06	0.16	0.136	0.06	−0.275	0.12	1.420	0.08	23.0	78.0
Thompson, Tristan	0.12	0.179	0.06	0.21	0.304	0.07	0.597	0.11	1.690	0.09	10.0	−24.0
Thybulle, Matisse	0.02	0.116	0.06	0.07	0.079	0.06	−1.296	0.20	0.583	0.13	−205.0	−60.0

	Off. β			Def. β			Off. γ		Def. γ		Discrepancy	
Name	Pr. Mean	Post. Mean	Post. SD	Pr. Mean	Post. Mean	Post. SD	Post. Mean	Post. SD	Post. Mean	Post. SD	Off.	Def.
Tillman, Xavier	0.07	0.059	0.06	0.16	0.109	0.07	−0.034	0.14	1.570	0.11	62.0	165.5
Toppin, Obi	0.04	−0.065	0.08	0.15	0.142	0.08	−0.633	0.23	1.266	0.12	187.0	18.0
Toscano-Anderson, Juan	0.03	−0.131	0.06	0.17	0.221	0.06	−0.796	0.21	1.283	0.10	194.0	−82.5
Towns, Karl-Anthony	0.07	0.138	0.06	0.24	0.254	0.06	−0.018	0.11	1.768	0.10	−21.0	6.5
Trent, Gary	0.01	−0.000	0.05	0.07	0.002	0.06	−1.631	0.20	0.516	0.11	−64.5	10.0
Tucker, P.J.	0.04	−0.067	0.06	0.11	0.086	0.06	−0.516	0.16	1.029	0.10	212.0	54.0
Turner, Myles	0.04	0.020	0.06	0.16	0.154	0.06	−0.367	0.15	1.349	0.09	95.0	30.0
Valanciunas, Jonas	0.13	0.332	0.06	0.29	0.405	0.07	0.720	0.10	2.201	0.09	−6.0	−1.0
Valentine, Denzel	0.02	0.016	0.06	0.17	0.252	0.06	−1.115	0.22	1.303	0.10	−10.0	−95.0
VanVleet, Fred	0.02	0.049	0.06	0.10	0.136	0.06	−1.488	0.19	0.770	0.09	−146.5	−131.0
Vanderbilt, Jarred	0.10	0.178	0.06	0.21	0.125	0.06	0.285	0.12	1.711	0.11	−19.0	162.0
Vassell, Devin	0.02	−0.072	0.07	0.14	0.130	0.06	−1.061	0.22	1.226	0.11	123.0	31.0
Vincent, Gabe	0.02	0.037	0.07	0.07	−0.027	0.07	−1.602	0.35	0.470	0.17	−142.5	17.0
Vucevic, Nikola	0.06	−0.022	0.05	0.29	0.383	0.06	0.030	0.10	1.974	0.06	222.5	−11.0
Wade, Dean	0.03	−0.092	0.06	0.15	0.137	0.06	−0.756	0.18	1.196	0.10	189.5	5.0
Wagner, Moritz	0.04	−0.024	0.07	0.15	0.112	0.07	−0.590	0.21	1.288	0.11	148.0	82.0
Walker, Kemba	0.01	0.011	0.06	0.11	0.037	0.06	−1.877	0.26	1.066	0.10	−112.0	129.0
Walker, Lonnie	0.01	−0.061	0.06	0.09	0.044	0.06	−1.986	0.29	0.794	0.11	−18.0	31.0
Wall, John	0.01	−0.023	0.06	0.08	0.046	0.06	−1.699	0.26	0.686	0.11	−36.0	1.0
Wanamaker, Brad	0.02	0.018	0.06	0.08	0.104	0.07	−1.425	0.26	0.423	0.12	−78.0	−120.0
Washington, P.J.	0.04	−0.013	0.06	0.16	0.169	0.06	−0.396	0.12	1.252	0.09	155.0	−24.0
Watanabe, Yuta	0.05	0.125	0.07	0.17	0.173	0.07	−0.445	0.18	1.363	0.11	−64.0	6.5
Westbrook, Russell	0.04	−0.020	0.06	0.25	0.152	0.06	−0.296	0.12	1.801	0.07	178.0	125.5
White, Coby	0.01	0.010	0.06	0.12	0.240	0.06	−1.685	0.20	0.956	0.08	−88.0	−206.0
White, Derrick	0.01	−0.034	0.07	0.09	0.025	0.07	−1.634	0.30	0.744	0.13	−15.0	43.0
Whiteside, Hassan	0.11	0.197	0.07	0.27	0.220	0.07	0.500	0.17	1.998	0.11	−3.0	46.0
Wiggins, Andrew	0.03	0.030	0.06	0.11	0.212	0.06	−0.584	0.13	0.815	0.09	47.0	−221.0
Williams, Grant	0.04	−0.000	0.06	0.11	0.098	0.06	−0.607	0.16	1.020	0.11	108.5	23.0
Williams, Kenrich	0.06	0.150	0.06	0.13	0.104	0.06	−0.174	0.13	1.183	0.09	−49.0	72.0
Williams, Lou	0.01	−0.029	0.06	0.08	0.075	0.06	−1.584	0.23	0.648	0.11	−14.5	−42.0
Williams, Patrick	0.03	0.041	0.06	0.13	0.196	0.06	−0.669	0.14	1.067	0.08	12.0	−115.0
Williams, Robert	0.14	0.208	0.07	0.23	0.167	0.07	0.594	0.13	1.868	0.09	−2.0	110.0
Williamson, Zion	0.08	0.106	0.06	0.13	0.110	0.06	0.240	0.10	1.184	0.09	23.0	58.0
Windler, Dylan	0.04	0.017	0.07	0.17	0.211	0.08	−0.653	0.25	1.399	0.14	67.0	−31.0
Winslow, Justise	0.03	−0.028	0.07	0.19	0.243	0.08	−0.687	0.26	1.744	0.14	143.0	12.0
Wiseman, James	0.06	0.006	0.07	0.19	0.145	0.07	0.148	0.17	1.470	0.11	185.0	80.0

	Off. β			Def. β			Off. γ		Def. γ		Discrepancy	
Name	Pr. Mean	Post. Mean	Post. SD	Pr. Mean	Post. Mean	Post. SD	Post. Mean	Post. SD	Post. Mean	Post. SD	Off.	Def.
Wood, Christian	0.06	0.028	0.06	0.23	0.248	0.06	−0.129	0.14	1.722	0.08	107.0	5.0
Wright, Delon	0.04	−0.006	0.06	0.12	0.030	0.06	−0.565	0.14	1.122	0.09	124.5	152.0
Young, Thaddeus	0.10	0.231	0.06	0.15	0.103	0.06	0.463	0.11	1.223	0.08	−15.0	83.0
Young, Trae	0.02	0.041	0.06	0.09	0.011	0.06	−1.208	0.18	0.875	0.10	−83.0	81.0
Zeller, Cody	0.12	0.203	0.07	0.21	0.253	0.07	0.545	0.13	1.558	0.11	−2.0	−28.0
Zubac, Ivica	0.12	0.192	0.06	0.20	0.349	0.06	0.552	0.11	1.640	0.09	3.0	−39.0

Part C

Tab. 1: False positive centers on offense.

Name	Label	Center Prob.
Bobby Portis	F	0.7774
Brandon Clarke	F	0.5192
Bruce Brown	G-F	0.5765
Derrick Favors	F	0.9979
Jarred Vanderbilt	F	0.9725
Kevon Looney	F	0.855
Marvin Bagley III	F	0.7416
Precious Achiuwa	F	0.9978
Richaun Holmes	F	0.9981
Serge Ibaka	F	0.9771
Taj Gibson	F	0.9918
Thaddeus Young	F	0.9966
Xavier Tillman Sr.	F	0.9165

Tab. 2: False negative centers on offense.

Name	Label	Center Prob.
Al Horford	C-F	0.2412
Alekesej Pokusevski	C	0.0
Brook Lopez	C	0.4571
Dean Wade	F-C	0.0053
Jalen McDaniels	F-C	0.0009
Julius Randle	F-C	0.0003
Kelly Olynyk	F-C	0.0385
Kevin Love	F-C	0.068
Lauri Markkanen	F-C	0.0136
Mike Muscala	F-C	0.0153
Myles Turner	C-F	0.3615

Tab. 3: False positive centers on defense.

Name	Label	Center Prob.
Blake Griffin	F	0.6853
Bobby Portis	F	0.9995
Christian Wood	F	0.9999
Darius Bazley	F-G	0.63
Derrick Favors	F	1.0
Draymond Green	F	0.9324
Eric Paschall	F	0.9257
Giannis Antetokounmpo	F	0.7901
Isaiah Roby	F	0.8878
Jarred Vanderbilt	F	0.9351
Juan Toscano-Anderson	F	0.5787
Kevon Looney	F	1.0
Marvin Bagley III	F	0.8526
Maxi Kleber	F	0.8928
Nicolo Melli	F	0.7364
Oshae Brissett	F-G	0.967
P.J. Washington	F	0.9618
Precious Achiuwa	F	1.0
Richaun Holmes	F	1.0
Serge Ibaka	F	1.0
Taj Gibson	F	0.9982
Xavier Tillman Sr.	F	0.9997
Yuta Watanabe	G-F	0.5727

Tab. 4: False negative centers on defense.

Name	Label	Center Prob.
Alekesej Pokusevski	C	0.102
Dean Wade	F-C	0.2493
JaMychal Green	F-C	0.0448
Jalen McDaniels	F-C	0.0861
Larry Nance Jr.	F-C	0.0195

Part D

See pages 15–16.

Tab. 5: Centers on offense.

Name	Label	Center Prob.
Alex Len	C	0.995
Andre Drummond	C	0.9998
Anthony Davis	F-C	0.9597
Aron Baynes	C-F	0.9326
Bam Adebayo	C-F	0.9858
Bismack Biyombo	C	0.9979
Bobby Portis	F	0.7774
Brandon Clarke	F	0.5192
Bruce Brown	G-F	0.5765
Chimezie Metu	F-C	0.7262
Chris Boucher	F-C	0.5028
Clint Capela	C	0.9988
Cody Zeller	F-C	0.9898
Damian Jones	C	0.9945
Daniel Gafford	F-C	0.9945
Daniel Theis	F-C	0.7629
Dario Saric	F-C	0.9687
DeAndre Jordan	C	0.9963
DeMarcus Cousins	C	0.5279
Deandre Ayton	C	0.9996
Derrick Favors	F	0.9979
Domantas Sabonis	F-C	0.9919
Drew Eubanks	F-C	0.9924
Dwight Howard	C-F	0.9986
Dwight Powell	F-C	0.9823
Enes Freedom	C	0.9984
Frank Kaminsky	F-C	0.5767

Name	Label	Center Prob.
Goga Bitadze	C-F	0.981
Gorgui Dieng	C	0.9033
Hassan Whiteside	C	0.9997
Isaiah Hartenstein	C-F	0.9997
Isaiah Stewart	F-C	0.9938
Ivica Zubac	C	0.9998
JaMychal Green	F-C	0.5038
JaVale McGee	C-F	0.9983
Jakob Poeltl	C	0.9971
James Wiseman	C	0.9815
Jarred Vanderbilt	F	0.9725
Jarrett Allen	C	0.9995
Jaxson Hayes	C-F	0.9474
Joel Embiid	C-F	0.9855
John Collins	F-C	0.8139
Jonas Valanciunas	C	0.9997
Jusuf Nurkic	C	0.9974
Karl-Anthony Towns	C-F	0.866
Kevon Looney	F	0.855
Khem Birch	C	0.9864
Kristaps Porzingis	F-C	0.8957
LaMarcus Aldridge	C-F	0.8844
Larry Nance Jr.	F-C	0.9448
Marc Gasol	C	0.7239
Marvin Bagley III	F	0.7416
Mason Plumlee	F-C	0.991
Mitchell Robinson	C-F	0.9541

Name	Label	Center Prob.
Mo Bamba	C	0.9326
Montrezl Harrell	F-C	0.9987
Moritz Wagner	F-C	0.6372
Moses Brown	C	0.9922
Naz Reid	C-F	0.8236
Nerlens Noel	C-F	0.9481
Nic Claxton	F-C	0.9834
Nikola Jokic	C	0.995
Nikola Vucevic	C	0.9822
Onyeka Okongwu	F-C	0.998
Precious Achiuwa	F	0.9978
Richaun Holmes	F	0.9981
Robert Williams III	C-F	0.9929
Robin Lopez	C	0.9971
Rudy Gobert	C	0.9993
Serge Ibaka	F	0.9771
Steven Adams	C	0.9989
Taj Gibson	F	0.9918
Thaddeus Young	F	0.9966
Tony Bradley	C-F	0.9951
Tristan Thompson	C-F	0.9952
Wendell Carter Jr.	C-F	0.9993
Willie Cauley-Stein	C	0.9733
Willy Hernangomez	C-F	0.9977
Xavier Tillman Sr.	F	0.9165

Tab. 6: Centers on defense.

Name	Label	Center Prob.
Al Horford	C-F	0.9973
Alex Len	C	1.0
Andre Drummond	C	0.9999
Anthony Davis	F-C	0.6205
Aron Baynes	C-F	0.9998
Bam Adebayo	C-F	0.9932
Bismack Biyombo	C	0.9999
Blake Griffin	F	0.6853
Bobby Portis	F	0.9995
Brook Lopez	C	1.0
Chimezie Metu	F-C	0.9981
Chris Boucher	F-C	1.0
Christian Wood	F	0.9999
Clint Capela	C	1.0
Cody Zeller	F-C	1.0
Damian Jones	C	0.9993
Daniel Gafford	F-C	1.0
Daniel Theis	F-C	0.9997
Dario Saric	F-C	0.9998
Darius Bazley	F-G	0.63
DeAndre Jordan	C	1.0
DeMarcus Cousins	C	1.0
Deandre Ayton	C	1.0
Derrick Favors	F	1.0
Domantas Sabonis	F-C	0.9999
Draymond Green	F	0.9324
Drew Eubanks	F-C	1.0
Dwight Howard	C-F	1.0
Dwight Powell	F-C	0.9991
Enes Freedom	C	1.0
Eric Paschall	F	0.9257
Frank Kaminsky	F-C	1.0

Name	Label	Center Prob.
Giannis Antetokounmpo	F	0.7901
Goga Bitadze	C-F	1.0
Gorgui Dieng	C	0.9997
Hassan Whiteside	C	1.0
Isaiah Hartenstein	C-F	1.0
Isaiah Roby	F	0.8878
Isaiah Stewart	F-C	1.0
Ivica Zubac	C	1.0
JaVale McGee	C-F	1.0
Jakob Poeltl	C	1.0
James Wiseman	C	1.0
Jarred Vanderbilt	F	0.9351
Jarrett Allen	C	1.0
Jaxson Hayes	C-F	0.9682
Joel Embiid	C-F	1.0
John Collins	F-C	0.9987
Jonas Valanciunas	C	1.0
Juan Toscano-Anderson	F	0.5787
Julius Randle	F-C	0.9336
Jusuf Nurkic	C	1.0
Karl-Anthony Towns	C-F	0.9997
Kelly Olynyk	F-C	0.9395
Kevin Love	F-C	0.9572
Kevon Looney	F	1.0
Khem Birch	C	0.914
Kristaps Porzingis	F-C	1.0
LaMarcus Aldridge	C-F	0.9993
Lauri Markkanen	F-C	0.8389
Marc Gasol	C	1.0
Marvin Bagley III	F	0.8526
Mason Plumlee	F-C	1.0
Maxi Kleber	F	0.8928

Name	Label	Center Prob.
Mike Muscala	F-C	1.0
Mitchell Robinson	C-F	0.9942
Mo Bamba	C	1.0
Montrezl Harrell	F-C	0.9993
Moritz Wagner	F-C	0.9997
Moses Brown	C	1.0
Myles Turner	C-F	1.0
Naz Reid	C-F	1.0
Nerlens Noel	C-F	1.0
Nic Claxton	F-C	0.9999
Nicolo Melli	F	0.7364
Nikola Jokic	C	1.0
Nikola Vucevic	C	1.0
Onyeka Okongwu	F-C	0.9998
Oshae Brissett	F-G	0.967
P.J. Washington	F	0.9618
Precious Achiuwa	F	1.0
Richaun Holmes	F	1.0
Robert Williams III	C-F	0.9987
Robin Lopez	C	1.0
Rudy Gobert	C	1.0
Serge Ibaka	F	1.0
Steven Adams	C	0.9963
Taj Gibson	F	0.9982
Tony Bradley	C-F	1.0
Tristan Thompson	C-F	0.9989
Wendell Carter Jr.	C-F	1.0
Willie Cauley-Stein	C	1.0
Willy Hernangomez	C-F	1.0
Xavier Tillman Sr.	F	0.9997
Yuta Watanabe	G-F	0.5727

Part E

See pages 18–25.

Tab. 7: Non-centers on offense (1).

Name	Label	0 Prob.	1 Prob.	2 Prob.	3 Prob.	4 Prob.
Aaron Gordon	F	0.0	0.9956	0.0	0.0044	0.0
Aaron Holiday	G	0.9296	0.0	0.0667	0.0037	0.0
Aaron Nesmith	G-F	0.0344	0.0	0.0472	0.9183	0.0
Al Horford	C-F	0.0	0.9994	0.0	0.0006	0.0
Alec Burks	G	0.8604	0.0	0.1336	0.006	0.0
Alekesej Pokusevski	C	0.8906	0.0	0.0444	0.065	0.0
Alex Caruso	G	0.0251	0.0002	0.9026	0.0722	0.0
Andre Iguodala	G-F	0.0	0.0007	0.0615	0.9378	0.0
Andrew Wiggins	F	0.0	0.0039	0.7374	0.2588	0.0
Anfernee Simons	G	0.9091	0.0	0.0333	0.0575	0.0
Anthony Edwards	G	0.159	0.0	0.84	0.0009	0.0
Armoni Brooks	G	0.6087	0.0	0.0148	0.3765	0.0
Austin Rivers	G	0.9466	0.0	0.0363	0.0171	0.0
Avery Bradley	G	0.645	0.0	0.0395	0.3155	0.0
Ben McLemore	G	0.4168	0.0	0.0203	0.563	0.0
Ben Simmons	G-F	0.0	1.0	0.0	0.0	0.0
Blake Griffin	F	0.0	0.9988	0.0	0.0012	0.0
Bogdan Bogdanovic	G	0.6469	0.0	0.3221	0.031	0.0
Bojan Bogdanovic	F	0.0012	0.0004	0.6728	0.3256	0.0
Brad Wanamaker	G	0.815	0.0	0.1846	0.0003	0.0
Bradley Beal	G	0.0	0.005	0.995	0.0	0.0
Brandon Goodwin	G	0.8899	0.0	0.1101	0.0	0.0
Brandon Ingram	F	0.0225	0.0001	0.9774	0.0	0.0
Brook Lopez	C	0.0	0.8817	0.0	0.1182	0.0
Bryn Forbes	G	0.4711	0.0	0.0082	0.5207	0.0
Buddy Hield	G	0.9227	0.0	0.0288	0.0485	0.0
CJ McCollum	G	0.8042	0.0	0.1958	0.0	0.0
Caleb Martin	F	0.041	0.0	0.3664	0.5926	0.0
Cam Reddish	F-G	0.0151	0.0	0.1986	0.7863	0.0
Cameron Johnson	F	0.2441	0.0	0.0386	0.7172	0.0
Cameron Payne	G	0.8204	0.0	0.1796	0.0	0.0
Caris LeVert	G	0.09	0.0	0.9099	0.0	0.0
Carmelo Anthony	F	0.0	0.1072	0.0105	0.8824	0.0
Cedi Osman	F	0.773	0.0	0.1967	0.0303	0.0
Chasson Randle	G	0.9305	0.0	0.0655	0.004	0.0
Chris Paul	G	0.4006	0.0	0.5994	0.0	0.0

Name	Label	0 Prob.	1 Prob.	2 Prob.	3 Prob.	4 Prob.
Christian Wood	F	0.0	0.9999	0.0	0.0001	0.0
Chuma Okeke	F	0.0	0.0009	0.0541	0.9451	0.0
Coby White	G	0.7815	0.0	0.2185	0.0	0.0
Cody Martin	F	0.0	0.0004	0.1865	0.8131	0.0
Cole Anthony	G	0.6183	0.0	0.3817	0.0	0.0
Collin Sexton	G	0.0	0.0024	0.9976	0.0	0.0
Cory Joseph	G	0.3979	0.0	0.6016	0.0005	0.0
D'Angelo Russell	G	0.0154	0.0001	0.9845	0.0	0.0
D.J. Augustin	G	0.88	0.0	0.12	0.0	0.0
DaQuan Jeffries	G-F	0.0278	0.0	0.044	0.9282	0.0
Damian Lillard	G	0.2425	0.0	0.7575	0.0	0.0
Damion Lee	G-F	0.6257	0.0	0.0118	0.3625	0.0
Damyean Dotson	G	0.9467	0.0	0.0364	0.0169	0.0
Danilo Gallinari	F	0.0	0.1577	0.005	0.8373	0.0
Danny Green	G	0.467	0.0	0.0142	0.5188	0.0
Danuel House Jr.	F-G	0.3192	0.0	0.1522	0.5286	0.0
Darius Bazley	F-G	0.0174	0.0001	0.5531	0.4294	0.0
Darius Garland	G	0.5777	0.0	0.4223	0.0	0.0
David Nwaba	G-F	0.0	0.0002	0.0422	0.9575	0.0
Davis Bertans	F	0.472	0.0	0.0077	0.5203	0.0
De'Aaron Fox	G	0.2152	0.0	0.7848	0.0	0.0
De'Andre Hunter	F-G	0.0	0.0198	0.1543	0.8259	0.0
De'Anthony Melton	G	0.2983	0.0	0.6442	0.0575	0.0
DeAndre' Bembry	G-F	0.0	0.002	0.3302	0.6678	0.0
DeMar DeRozan	G-F	0.0	0.0222	0.9778	0.0	0.0
Dean Wade	F-C	0.0	0.0001	0.0477	0.9522	0.0
Dejounte Murray	G	0.7145	0.0	0.2855	0.0	0.0
Delon Wright	G	0.2953	0.0	0.7046	0.0001	0.0
Deni Avdija	F	0.0271	0.0	0.0436	0.9293	0.0
Dennis Schroder	G	0.3095	0.0	0.6905	0.0	0.0
Denzel Valentine	G	0.8773	0.0	0.0428	0.0799	0.0
Derrick Jones Jr.	F	0.0	0.0008	0.0354	0.9638	0.0
Derrick Rose	G	0.0981	0.0	0.9019	0.0	0.0
Derrick White	G	0.7524	0.0	0.2464	0.0011	0.0
Desmond Bane	G	0.7307	0.0	0.0212	0.2481	0.0
Devin Booker	G	0.0	0.9244	0.0756	0.0	0.0

Tab. 8: Non-centers on offense (2).

Name	Label	0 Prob.	1 Prob.	2 Prob.	3 Prob.	4 Prob.
Devin Vassell	G-F	0.3218	0.0	0.0268	0.6514	0.0
Devonte' Graham	G	0.8353	0.0	0.1647	0.0	0.0
Dillon Brooks	G-F	0.0944	0.0001	0.8524	0.0531	0.0
Donovan Mitchell	G	0.2583	0.0	0.7417	0.0	0.0
Donte DiVincenzo	G	0.001	0.0002	0.2756	0.7232	0.0
Dorian Finney-Smith	F	0.002	0.0	0.05	0.948	0.0
Doug McDermott	F	0.0344	0.0	0.0737	0.8919	0.0
Draymond Green	F	0.0	0.9984	0.0	0.0016	0.0
Duncan Robinson	F	0.5655	0.0	0.011	0.4235	0.0
Dwayne Bacon	G-F	0.8709	0.0	0.0878	0.0413	0.0
Dylan Windler	G-F	0.0036	0.0	0.0712	0.9252	0.0
Edmond Sumner	G	0.5344	0.0	0.0827	0.3829	0.0
Elfrid Payton	G	0.0	0.0017	0.9982	0.0	0.0
Eric Bledsoe	G	0.9322	0.0	0.0674	0.0005	0.0
Eric Gordon	G	0.9124	0.0	0.0873	0.0003	0.0
Eric Paschall	F	0.0	0.9951	0.0	0.0049	0.0
Evan Fournier	G-F	0.9234	0.0	0.0725	0.004	0.0
Facundo Campazzo	G	0.9462	0.0	0.0516	0.0022	0.0
Frank Jackson	G	0.2249	0.0	0.2682	0.5069	0.0
Fred VanVleet	G	0.7168	0.0	0.2832	0.0	0.0
Furkan Korkmaz	G-F	0.9136	0.0	0.029	0.0573	0.0
Gabe Vincent	G	0.9384	0.0	0.0378	0.0238	0.0
Garrett Temple	G-F	0.3487	0.0	0.0412	0.6101	0.0
Garrison Mathews	G	0.458	0.0	0.0067	0.5354	0.0
Gary Clark	F	0.0002	0.0001	0.0552	0.9446	0.0
Gary Harris	G	0.0	0.0013	0.2195	0.7792	0.0
Gary Trent Jr.	G-F	0.829	0.0	0.0214	0.1495	0.0
George Hill	G	0.5211	0.0	0.4669	0.012	0.0
Georges Niang	F	0.5367	0.0	0.0155	0.4478	0.0
Giannis Antetokounmpo	F	0.0	0.0	0.0	0.0	1.0
Goran Dragic	G	0.0087	0.0002	0.9912	0.0	0.0
Gordon Hayward	F	0.0	0.0076	0.9792	0.0132	0.0
Grant Williams	F	0.0	0.0223	0.0052	0.9725	0.0
Grayson Allen	G	0.8901	0.0	0.0249	0.085	0.0
Hamidou Diallo	G	0.0003	0.0009	0.8781	0.1207	0.0
Harrison Barnes	F	0.0	0.3087	0.0049	0.6865	0.0

Name	Label	0 Prob.	1 Prob.	2 Prob.	3 Prob.	4 Prob.
Immanuel Quickley	G	0.881	0.0	0.119	0.0	0.0
Isaac Okoro	F-G	0.0023	0.0001	0.1586	0.839	0.0
Isaiah Roby	F	0.0	0.992	0.0	0.008	0.0
Ish Smith	G	0.8196	0.0	0.1804	0.0	0.0
JJ Redick	G	0.8206	0.0	0.0278	0.1516	0.0
Ja Morant	G	0.0	0.0011	0.9989	0.0	0.0
Jaden McDaniels	F	0.0032	0.0	0.0472	0.9496	0.0
Jae Crowder	F	0.0424	0.0	0.0469	0.9107	0.0
Jae'Sean Tate	F	0.0	0.8245	0.0	0.1755	0.0
Jake Layman	F	0.0089	0.0	0.0518	0.9394	0.0
Jalen Brunson	G	0.8673	0.0	0.1327	0.0	0.0
Jalen McDaniels	F-C	0.0	0.0003	0.0984	0.9013	0.0
Jamal Murray	G	0.004	0.0003	0.9957	0.0	0.0
James Ennis III	F	0.0089	0.0	0.0832	0.9079	0.0
James Harden	G	0.0202	0.0	0.9798	0.0	0.0
James Johnson	F	0.0	0.0012	0.1379	0.8609	0.0
Jarrett Culver	G-F	0.0	0.0018	0.1891	0.8091	0.0
Jaylen Brown	G-F	0.0	0.0276	0.9662	0.0062	0.0
Jaylen Nowell	G	0.7053	0.0	0.2335	0.0612	0.0
Jayson Tatum	F-G	0.0	0.7718	0.2282	0.0	0.0
Jeff Green	F	0.0	0.0041	0.0149	0.9809	0.0
Jeff Teague	G	0.7611	0.0	0.2389	0.0	0.0
Jerami Grant	F	0.0	0.0036	0.9914	0.005	0.0
Jeremy Lamb	G-F	0.6556	0.0	0.2927	0.0517	0.0
Jevon Carter	G	0.8447	0.0	0.0214	0.1339	0.0
Jimmy Butler	F	0.0	1.0	0.0	0.0	0.0
Joe Harris	G-F	0.3295	0.0	0.0452	0.6253	0.0
Joe Ingles	F-G	0.884	0.0	0.116	0.0	0.0
John Konchar	G	0.0	0.0002	0.045	0.9547	0.0
John Wall	G	0.3955	0.0	0.6045	0.0	0.0
Jordan Clarkson	G	0.5584	0.0	0.4414	0.0002	0.0
Jordan McLaughlin	G	0.8211	0.0	0.1789	0.0	0.0
Jordan Poole	G	0.9283	0.0	0.0707	0.001	0.0
Josh Hart	G	0.2081	0.0	0.073	0.7189	0.0
Josh Jackson	G-F	0.0004	0.0009	0.9218	0.077	0.0
Josh Okogie	G	0.0	0.0015	0.0234	0.9751	0.0

Tab. 9: Non-centers on offense (3).

Name	Label	0 Prob.	1 Prob.	2 Prob.	3 Prob.	4 Prob.
Josh Richardson	G	0.7618	0.0	0.1907	0.0475	0.0
Jrue Holiday	G	0.0	0.0251	0.974	0.0009	0.0
Juan Toscano-Anderson	F	0.0	0.0151	0.0203	0.9646	0.0
Juancho Hernangomez	F	0.0	0.0016	0.0215	0.9769	0.0
Julius Randle	F-C	0.0	1.0	0.0	0.0	0.0
Justin Holiday	F-G	0.4647	0.0	0.0135	0.5217	0.0
Justin Jackson	F	0.8415	0.0	0.1054	0.053	0.0
Justise Winslow	F-G	0.0	0.0179	0.3681	0.614	0.0
Kawhi Leonard	F	0.0	1.0	0.0	0.0	0.0
Keldon Johnson	F-G	0.0007	0.0002	0.2975	0.7016	0.0
Kelly Olynyk	F-C	0.0	0.8427	0.0	0.1573	0.0
Kelly Oubre Jr.	F-G	0.0	0.0012	0.0692	0.9296	0.0
Kemba Walker	G	0.8192	0.0	0.1808	0.0	0.0
Kendrick Nunn	G	0.821	0.0	0.1487	0.0302	0.0
Kenrich Williams	G-F	0.0	0.0012	0.3176	0.6812	0.0
Kent Bazemore	G-F	0.0	0.0002	0.1199	0.8799	0.0
Kentavious Caldwell-Pope	G	0.5957	0.0	0.0225	0.3818	0.0
Kenyon Martin Jr.	F	0.0	0.0216	0.0052	0.9732	0.0
Kevin Durant	F	0.0	1.0	0.0	0.0	0.0
Kevin Huerter	G-F	0.8821	0.0	0.0542	0.0637	0.0
Kevin Love	F-C	0.0	0.991	0.0	0.009	0.0
Kevin Porter Jr.	G-F	0.307	0.0	0.693	0.0	0.0
Khris Middleton	F	0.0	0.01	0.9888	0.0013	0.0
Killian Hayes	G	0.7832	0.0	0.2168	0.0	0.0
Kira Lewis Jr.	G	0.8985	0.0	0.1001	0.0014	0.0
Kyle Anderson	F-G	0.0003	0.0007	0.6641	0.3349	0.0
Kyle Kuzma	F	0.0001	0.0002	0.1424	0.8574	0.0
Kyle Lowry	G	0.4878	0.0	0.5122	0.0	0.0
Kyrie Irving	G	0.001	0.0004	0.9986	0.0	0.0
LaMelo Ball	G	0.0228	0.0001	0.9772	0.0	0.0
Lamar Stevens	F	0.0	0.0163	0.0139	0.9698	0.0
Landry Shamet	G	0.6985	0.0	0.0143	0.2871	0.0
Lauri Markkanen	F-C	0.0	0.004	0.014	0.982	0.0
LeBron James	F	0.0	1.0	0.0	0.0	0.0
Lonnie Walker IV	G-F	0.9301	0.0	0.0308	0.0391	0.0
Lonzo Ball	G	0.8978	0.0	0.1002	0.002	0.0

Name	Label	0 Prob.	1 Prob.	2 Prob.	3 Prob.	4 Prob.
Lou Williams	G	0.6909	0.0	0.3091	0.0	0.0
Luguentz Dort	G	0.8035	0.0	0.1633	0.0332	0.0
Luka Doncic	F-G	0.0002	0.0003	0.9995	0.0	0.0
Luke Kennard	G	0.6782	0.0	0.1004	0.2214	0.0
Malachi Flynn	G	0.9087	0.0	0.0912	0.0001	0.0
Malcolm Brogdon	G	0.388	0.0	0.612	0.0	0.0
Malik Beasley	G	0.7346	0.0	0.1442	0.1212	0.0
Malik Monk	G	0.9387	0.0	0.0603	0.001	0.0
Marcus Morris Sr.	F	0.0	0.022	0.0119	0.9661	0.0
Marcus Smart	G	0.0002	0.0012	0.9618	0.0369	0.0
Markieff Morris	F	0.0	0.3024	0.0003	0.6974	0.0
Matisse Thybulle	G-F	0.3029	0.0	0.02	0.677	0.0
Maurice Harkless	F-G	0.0027	0.0	0.0477	0.9496	0.0
Max Strus	G-F	0.4046	0.0	0.0056	0.5898	0.0
Maxi Kleber	F	0.0	0.0002	0.0492	0.9506	0.0
Michael Carter-Williams	G	0.0032	0.0003	0.9965	0.0	0.0
Michael Porter Jr.	F	0.0	0.0788	0.0017	0.9195	0.0
Mikal Bridges	F	0.0005	0.0	0.0731	0.9264	0.0
Mike Conley	G	0.6672	0.0	0.3328	0.0	0.0
Mike Muscala	F-C	0.0	0.0074	0.0103	0.9822	0.0
Mike Scott	F	0.2891	0.0	0.0208	0.6901	0.0
Miles Bridges	F	0.0	0.0721	0.0057	0.9221	0.0
Miye Oni	G-F	0.0092	0.0	0.0472	0.9436	0.0
Monte Morris	G	0.7315	0.0	0.2669	0.0016	0.0
Mychal Mulder	G	0.4762	0.0	0.0084	0.5154	0.0
Myles Turner	C-F	0.0	0.7785	0.0	0.2215	0.0
Naji Marshall	F	0.5209	0.0	0.2631	0.2159	0.0
Nassir Little	F-G	0.0126	0.0	0.052	0.9354	0.0
Nemanja Bjelica	F	0.0	0.1337	0.022	0.8443	0.0
Nickeil Alexander-Walker	G	0.9312	0.0	0.0677	0.0011	0.0
Nicolas Batum	G-F	0.0	0.0003	0.0341	0.9655	0.0
Nicolo Melli	F	0.0	0.029	0.0042	0.9668	0.0
Norman Powell	G	0.806	0.0	0.1538	0.0402	0.0
OG Anunoby	F	0.0	0.0309	0.0099	0.9592	0.0
Obi Toppin	F	0.0	0.0033	0.0157	0.981	0.0
Oshae Brissett	F-G	0.0	0.0252	0.0064	0.9684	0.0

Tab. 10: Non-centers on offense (4).

Name	Label	0 Prob.	1 Prob.	2 Prob.	3 Prob.	4 Prob.
Otto Porter Jr.	F	0.0	0.0005	0.0659	0.9336	0.0
P.J. Dozier	G-F	0.0002	0.0003	0.3239	0.6756	0.0
P.J. Tucker	F	0.0	0.0003	0.05	0.9497	0.0
P.J. Washington	F	0.0	0.9992	0.0	0.0008	0.0
Pascal Siakam	F	0.0	1.0	0.0	0.0	0.0
Pat Connaughton	G	0.006	0.0	0.0463	0.9477	0.0
Patrick Beverley	G	0.2786	0.0	0.4841	0.2373	0.0
Patrick Patterson	F	0.0	0.0001	0.0498	0.95	0.0
Patrick Williams	F	0.0005	0.0	0.0769	0.9226	0.0
Patty Mills	G	0.9264	0.0	0.0299	0.0437	0.0
Paul George	F	0.0001	0.0012	0.9988	0.0	0.0
Paul Millsap	F	0.0	0.9945	0.0	0.0055	0.0
Payton Pritchard	G	0.9448	0.0	0.0467	0.0085	0.0
R.J. Hampton	G	0.1128	0.0001	0.8309	0.0562	0.0
RJ Barrett	F-G	0.0033	0.0004	0.975	0.0213	0.0
Rajon Rondo	G	0.1224	0.0	0.8776	0.0	0.0
Raul Neto	G	0.9087	0.0	0.0781	0.0132	0.0
Reggie Bullock	G-F	0.5069	0.0	0.0097	0.4834	0.0
Reggie Jackson	G	0.8838	0.0	0.1154	0.0008	0.0
Ricky Rubio	G	0.7579	0.0	0.2421	0.0	0.0
Robert Covington	F	0.0046	0.0	0.0468	0.9486	0.0
Rodney Hood	G-F	0.1723	0.0	0.1087	0.719	0.0
Royce O'Neale	F	0.0673	0.0	0.0559	0.8768	0.0
Rudy Gay	F-G	0.0	0.0028	0.1816	0.8156	0.0
Rui Hachimura	F	0.0	0.9519	0.0	0.0481	0.0
Russell Westbrook	G	0.0	0.0097	0.9903	0.0	0.0
Saben Lee	G	0.061	0.0	0.9389	0.0	0.0
Saddiq Bey	F	0.0174	0.0	0.0961	0.8864	0.0
Sekou Doumbouya	F	0.0	0.1369	0.0009	0.8622	0.0
Semi Ojeleye	F	0.0064	0.0	0.0473	0.9463	0.0
Seth Curry	G	0.9322	0.0	0.0333	0.0345	0.0
Shai Gilgeous-Alexander	G-F	0.0869	0.0	0.9131	0.0	0.0
Shake Milton	G-F	0.6718	0.0	0.3282	0.0	0.0
Solomon Hill	F	0.2236	0.0	0.027	0.7494	0.0
Stanley Johnson	F-G	0.037	0.0	0.1048	0.8582	0.0
Stephen Curry	G	0.3372	0.0	0.6627	0.0	0.0

Name	Label	0 Prob.	1 Prob.	2 Prob.	3 Prob.	4 Prob.
Sterling Brown	G-F	0.0753	0.0	0.0627	0.862	0.0
Svi Mykhailiuk	G-F	0.9304	0.0	0.0439	0.0257	0.0
T.J. McConnell	G	0.1387	0.0	0.8613	0.0	0.0
Talen Horton-Tucker	G	0.4684	0.0	0.5316	0.0	0.0
Taurean Prince	F	0.7667	0.0	0.1289	0.1044	0.0
Terance Mann	G-F	0.0007	0.0002	0.2912	0.708	0.0
Terence Davis	G	0.8724	0.0	0.0607	0.0669	0.0
Terrence Ross	G-F	0.3044	0.0	0.6023	0.0933	0.0
Terry Rozier	G	0.8205	0.0	0.1794	0.0001	0.0
Thanasis Antetokounmpo	F	0.0	0.935	0.0	0.065	0.0
Theo Maledon	G	0.9087	0.0	0.0912	0.0001	0.0
Tim Hardaway Jr.	G-F	0.8614	0.0	0.0339	0.1047	0.0
Timothe Luwawu-Cabarrot	G-F	0.3083	0.0	0.0344	0.6573	0.0
Tobias Harris	F	0.0	0.9112	0.0702	0.0186	0.0
Tomas Satoransky	G	0.0041	0.0003	0.9953	0.0003	0.0
Tony Snell	G	0.4562	0.0	0.0142	0.5296	0.0
Torrey Craig	F	0.0	0.0027	0.0176	0.9797	0.0
Trae Young	G	0.0323	0.0	0.9677	0.0	0.0
Trevor Ariza	F	0.0	0.0002	0.045	0.9547	0.0
Trey Burke	G	0.9474	0.0	0.0461	0.0065	0.0
Troy Brown Jr.	G-F	0.0003	0.0001	0.0786	0.921	0.0
Ty Jerome	G-F	0.9312	0.0	0.0685	0.0004	0.0
Tyler Herro	G	0.9073	0.0	0.0926	0.0002	0.0
Tyler Johnson	G	0.6044	0.0	0.0146	0.3811	0.0
Tyrese Haliburton	G	0.9109	0.0	0.089	0.0001	0.0
Tyrese Maxey	G	0.8577	0.0	0.1423	0.0	0.0
Tyus Jones	G	0.8686	0.0	0.1314	0.0	0.0
Victor Oladipo	G	0.5815	0.0	0.4185	0.0	0.0
Wayne Ellington	G	0.4568	0.0	0.0113	0.5318	0.0
Wes Iwundu	F	0.089	0.0	0.0426	0.8684	0.0
Wesley Matthews	G	0.0997	0.0	0.0393	0.8611	0.0
Will Barton	G	0.0048	0.0003	0.8428	0.1521	0.0
Yuta Watanabe	G-F	0.0011	0.0	0.0533	0.9456	0.0
Zach LaVine	G-F	0.0183	0.0001	0.9816	0.0	0.0
Zion Williamson	F	0.0	0.0	0.0	0.0	1.0

Tab. 11: Non-centers on defense (1).

Name	Label	0 Prob.	1 Prob.	2 Prob.	3 Prob.
Aaron Gordon	F	0.7057	0.294	0.0	0.0003
Aaron Holiday	G	0.072	0.0156	0.0131	0.8994
Aaron Nesmith	G-F	0.3201	0.6761	0.0	0.0038
Alec Burks	G	0.6134	0.3352	0.0	0.0513
Alekesej Pokusevski	C	0.0151	0.9849	0.0	0.0
Alex Caruso	G	0.0	0.0001	0.9821	0.0178
Andre Iguodala	G-F	0.0429	0.1237	0.0007	0.8328
Andrew Wiggins	F	0.2045	0.7886	0.0	0.0069
Anfernee Simons	G	0.6036	0.3928	0.0	0.0036
Anthony Edwards	G	0.9012	0.0578	0.0	0.041
Armoni Brooks	G	0.9551	0.0392	0.0	0.0057
Austin Rivers	G	0.4991	0.0054	0.0001	0.4955
Avery Bradley	G	0.4088	0.083	0.0011	0.5072
Ben McLemore	G	0.8332	0.1233	0.0	0.0435
Ben Simmons	G-F	0.1564	0.0184	0.005	0.8203
Bogdan Bogdanovic	G	0.3389	0.582	0.0	0.0791
Bojan Bogdanovic	F	0.8047	0.195	0.0	0.0003
Brad Wanamaker	G	0.0611	0.0067	0.0198	0.9125
Bradley Beal	G	0.9187	0.0129	0.0	0.0685
Brandon Clarke	F	0.0159	0.9831	0.0	0.001
Brandon Goodwin	G	0.8002	0.1522	0.0	0.0475
Brandon Ingram	F	0.8218	0.1779	0.0	0.0003
Bruce Brown	G-F	0.0657	0.8048	0.0	0.1295
Bryn Forbes	G	0.9691	0.0295	0.0	0.0014
Buddy Hield	G	0.4341	0.5471	0.0	0.0188
CJ McCollum	G	0.8106	0.0624	0.0001	0.127
Caleb Martin	F	0.0012	0.0526	0.0007	0.9454
Cam Reddish	F-G	0.0145	0.007	0.0981	0.8804
Cameron Johnson	F	0.5496	0.3965	0.0	0.0539
Cameron Payne	G	0.5125	0.1883	0.0004	0.2988
Caris LeVert	G	0.3999	0.0353	0.0011	0.5637
Carmelo Anthony	F	0.7152	0.2847	0.0	0.0
Cedi Osman	F	0.9337	0.0243	0.0	0.042
Chasson Randle	G	0.6232	0.0246	0.0002	0.352

Name	Label	0 Prob.	1 Prob.	2 Prob.	3 Prob.
Chris Paul	G	0.8223	0.0045	0.0	0.1732
Chuma Okeke	F	0.0743	0.3236	0.0001	0.6021
Coby White	G	0.788	0.1671	0.0	0.0449
Cody Martin	F	0.0136	0.174	0.0	0.8124
Cole Anthony	G	0.5374	0.3381	0.0001	0.1244
Collin Sexton	G	0.8974	0.0009	0.0	0.1018
Cory Joseph	G	0.1319	0.118	0.0015	0.7486
D'Angelo Russell	G	0.8031	0.024	0.0	0.1728
D.J. Augustin	G	0.9858	0.0014	0.0	0.0128
DaQuan Jeffries	G-F	0.5052	0.4263	0.0	0.0685
Damian Lillard	G	0.9756	0.0022	0.0	0.0221
Damion Lee	G-F	0.2896	0.6328	0.0	0.0776
Damyean Dotson	G	0.9424	0.0558	0.0	0.0018
Danilo Gallinari	F	0.049	0.951	0.0	0.0
Danny Green	G	0.0056	0.1604	0.0	0.834
Danuel House Jr.	F-G	0.6492	0.3334	0.0	0.0173
Darius Garland	G	0.1508	0.0023	0.0016	0.8454
David Nwaba	G-F	0.0091	0.0374	0.0054	0.948
Davis Bertans	F	0.5387	0.4608	0.0	0.0006
De'Aaron Fox	G	0.2079	0.0047	0.0013	0.7861
De'Andre Hunter	F-G	0.3528	0.6231	0.0	0.0241
De'Anthony Melton	G	0.0053	0.0215	0.0239	0.9493
DeAndre' Bembry	G-F	0.0965	0.0066	0.0092	0.8877
DeMar DeRozan	G-F	0.9945	0.0039	0.0	0.0015
Dean Wade	F-C	0.0275	0.9721	0.0	0.0003
Dejounte Murray	G	0.1915	0.6481	0.0	0.1604
Delon Wright	G	0.0103	0.0025	0.2594	0.7278
Deni Avdija	F	0.0137	0.9863	0.0	0.0
Dennis Schroder	G	0.0732	0.0099	0.0146	0.9023
Denzel Valentine	G	0.5438	0.4346	0.0	0.0216
Derrick Jones Jr.	F	0.0847	0.1153	0.0014	0.7987
Derrick Rose	G	0.4238	0.004	0.0001	0.5721
Derrick White	G	0.1603	0.4227	0.0001	0.417
Desmond Bane	G	0.6675	0.0623	0.0003	0.2699

Tab. 12: Non-centers on defense (2).

Name	Label	0 Prob.	1 Prob.	2 Prob.	3 Prob.
Devin Booker	G	0.8621	0.1357	0.0	0.0022
Devin Vassell	G-F	0.1552	0.3035	0.0002	0.5411
Devonte' Graham	G	0.4109	0.0038	0.0001	0.5852
Dillon Brooks	G-F	0.0179	0.0059	0.094	0.8822
Donovan Mitchell	G	0.9847	0.0018	0.0	0.0135
Donte DiVincenzo	G	0.3955	0.2873	0.0003	0.317
Dorian Finney-Smith	F	0.3791	0.6083	0.0	0.0126
Doug McDermott	F	0.6373	0.3618	0.0	0.0009
Duncan Robinson	F	0.4643	0.5194	0.0	0.0163
Dwayne Bacon	G-F	0.8381	0.1238	0.0	0.0381
Dylan Windler	G-F	0.0889	0.7509	0.0	0.1602
Edmond Sumner	G	0.1344	0.0037	0.0033	0.8586
Elfrid Payton	G	0.0552	0.0355	0.0079	0.9014
Eric Bledsoe	G	0.6678	0.0964	0.0003	0.2355
Eric Gordon	G	0.998	0.0018	0.0	0.0002
Evan Fournier	G-F	0.4695	0.1029	0.0008	0.4269
Facundo Campazzo	G	0.0	0.0	1.0	0.0
Frank Jackson	G	0.848	0.0204	0.0	0.1316
Fred VanVleet	G	0.0184	0.005	0.1018	0.8748
Furkan Korkmaz	G-F	0.4116	0.0107	0.0004	0.5773
Gabe Vincent	G	0.0	0.0	0.9919	0.008
Garrett Temple	G-F	0.1312	0.0578	0.0037	0.8073
Garrison Mathews	G	0.3097	0.0217	0.0016	0.6671
Gary Clark	F	0.0468	0.9531	0.0	0.0001
Gary Harris	G	0.0514	0.0006	0.0143	0.9336
Gary Trent Jr.	G-F	0.7829	0.0023	0.0	0.2148
George Hill	G	0.193	0.0143	0.0034	0.7894
Georges Niang	F	0.5502	0.4479	0.0	0.0019
Goran Dragic	G	0.9116	0.0288	0.0	0.0596
Gordon Hayward	F	0.8036	0.1695	0.0	0.0268
Grant Williams	F	0.0466	0.9486	0.0	0.0048
Grayson Allen	G	0.7627	0.1118	0.0001	0.1254
Hamidou Diallo	G	0.5714	0.3244	0.0001	0.1041
Harrison Barnes	F	0.3252	0.6747	0.0	0.0001

Name	Label	0 Prob.	1 Prob.	2 Prob.	3 Prob.
Immanuel Quickley	G	0.7093	0.0735	0.0002	0.217
Isaac Okoro	F-G	0.1093	0.0789	0.0027	0.809
Ish Smith	G	0.17	0.0135	0.0041	0.8124
JJ Redick	G	0.98	0.0051	0.0	0.0149
Ja Morant	G	0.9701	0.0233	0.0	0.0066
JaMychal Green	F-C	0.0996	0.9003	0.0	0.0001
Jaden McDaniels	F	0.0231	0.9638	0.0	0.0131
Jae Crowder	F	0.2627	0.735	0.0	0.0023
Jae'Sean Tate	F	0.0336	0.071	0.0022	0.8933
Jake Layman	F	0.2132	0.0954	0.0019	0.6895
Jalen Brunson	G	0.9286	0.062	0.0	0.0095
Jalen McDaniels	F-C	0.0121	0.9526	0.0	0.0352
Jamal Murray	G	0.15	0.0051	0.0032	0.8417
James Ennis III	F	0.2122	0.0814	0.0022	0.7042
James Harden	G	0.6247	0.3753	0.0	0.0
James Johnson	F	0.1542	0.8388	0.0	0.0069
Jarrett Culver	G-F	0.1693	0.1352	0.0013	0.6942
Jaylen Brown	G-F	0.7836	0.1066	0.0001	0.1097
Jaylen Nowell	G	0.5451	0.3122	0.0001	0.1425
Jayson Tatum	F-G	0.8539	0.1455	0.0	0.0006
Jeff Green	F	0.0936	0.9064	0.0	0.0
Jeff Teague	G	0.4759	0.0228	0.0005	0.5008
Jerami Grant	F	0.7937	0.206	0.0	0.0003
Jeremy Lamb	G-F	0.0721	0.8788	0.0	0.049
Jevon Carter	G	0.075	0.0087	0.0143	0.902
Jimmy Butler	F	0.0204	0.0101	0.0522	0.9173
Joe Harris	G-F	0.6351	0.3636	0.0	0.0014
Joe Ingles	F-G	0.9745	0.0238	0.0	0.0016
John Konchar	G	0.0197	0.2062	0.0	0.7741
John Wall	G	0.9624	0.0128	0.0	0.0248
Jordan Clarkson	G	0.8596	0.037	0.0	0.1034
Jordan McLaughlin	G	0.0031	0.0008	0.6418	0.3542
Jordan Poole	G	0.9801	0.0044	0.0	0.0154
Josh Hart	G	0.0109	0.989	0.0	0.0001

Tab. 13: Non-centers on defense (3).

Name	Label	0 Prob.	1 Prob.	2 Prob.	3 Prob.
Josh Jackson	G-F	0.3144	0.1692	0.0008	0.5156
Josh Okogie	G	0.0002	0.0006	0.9053	0.0939
Josh Richardson	G	0.3234	0.0047	0.0003	0.6715
Jrue Holiday	G	0.0161	0.002	0.1837	0.7981
Juancho Hernangomez	F	0.0353	0.9647	0.0	0.0
Justin Holiday	F-G	0.3562	0.371	0.0002	0.2727
Justin Jackson	F	0.8182	0.1466	0.0	0.0352
Justise Winslow	F-G	0.0537	0.9463	0.0	0.0
Kawhi Leonard	F	0.7467	0.1502	0.0001	0.103
Keldon Johnson	F-G	0.1058	0.8942	0.0	0.0001
Kelly Oubre Jr.	F-G	0.2986	0.531	0.0	0.1704
Kemba Walker	G	0.0994	0.0198	0.0086	0.8721
Kendrick Nunn	G	0.443	0.1303	0.0007	0.426
Kenrich Williams	G-F	0.1943	0.2903	0.0003	0.5151
Kent Bazemore	G-F	0.0149	0.2173	0.0	0.7678
Kentavious Caldwell-Pope	G	0.5857	0.0155	0.0002	0.3987
Kenyon Martin Jr.	F	0.211	0.789	0.0	0.0
Kevin Durant	F	0.0524	0.9476	0.0	0.0
Kevin Huerter	G-F	0.2989	0.0359	0.0019	0.6633
Kevin Porter Jr.	G-F	0.9128	0.0636	0.0	0.0235
Khris Middleton	F	0.6959	0.2952	0.0	0.0088
Killian Hayes	G	0.0551	0.0213	0.0133	0.9103
Kira Lewis Jr.	G	0.0472	0.0028	0.0301	0.9199
Kyle Anderson	F-G	0.1286	0.8295	0.0	0.0418
Kyle Kuzma	F	0.2129	0.787	0.0	0.0001
Kyle Lowry	G	0.7299	0.1595	0.0001	0.1105
Kyrie Irving	G	0.5312	0.0142	0.0002	0.4544
LaMelo Ball	G	0.0687	0.0069	0.0165	0.9079
Lamar Stevens	F	0.0379	0.9573	0.0	0.0047
Landry Shamet	G	0.652	0.3282	0.0	0.0197
Larry Nance Jr.	F-C	0.0326	0.5074	0.0	0.4601
LeBron James	F	0.6759	0.324	0.0	0.0
Lonnie Walker IV	G-F	0.9038	0.0893	0.0	0.0068
Lonzo Ball	G	0.2388	0.1186	0.0014	0.6412
Lou Williams	G	0.9725	0.0009	0.0	0.0266
Luguentz Dort	G	0.2922	0.0627	0.0019	0.6432
Luka Doncic	F-G	0.2139	0.7861	0.0	0.0
Luke Kennard	G	0.4814	0.5185	0.0	0.0001
Malachi Flynn	G	0.0274	0.0095	0.0432	0.9198
Malcolm Brogdon	G	0.8365	0.1282	0.0	0.0353
Malik Beasley	G	0.9601	0.024	0.0	0.0158
Malik Monk	G	0.9704	0.0282	0.0	0.0014
Marcus Morris Sr.	F	0.4021	0.5978	0.0	0.0
Marcus Smart	G	0.0398	0.0029	0.0416	0.9156
Markieff Morris	F	0.0741	0.9259	0.0	0.0
Matisse Thybulle	G-F	0.0	0.0	1.0	0.0
Maurice Harkless	F-G	0.0931	0.1562	0.0008	0.75
Max Strus	G-F	0.1283	0.0435	0.0047	0.8235
Michael Carter-Williams	G	0.2384	0.1078	0.0016	0.6522
Michael Porter Jr.	F	0.0608	0.9392	0.0	0.0
Mikal Bridges	F	0.2222	0.2236	0.0006	0.5537
Mike Conley	G	0.4115	0.0065	0.0002	0.5818
Mike Scott	F	0.1013	0.8985	0.0	0.0003
Miles Bridges	F	0.034	0.9658	0.0	0.0002
Miye Oni	G-F	0.0878	0.8972	0.0	0.015
Monte Morris	G	0.8879	0.023	0.0	0.0891
Mychal Mulder	G	0.3803	0.6122	0.0	0.0075
Naji Marshall	F	0.3517	0.6267	0.0	0.0217
Nassir Little	F-G	0.0073	0.9927	0.0	0.0
Nemanja Bjelica	F	0.0162	0.9837	0.0	0.0
Nickeil Alexander-Walker	G	0.0836	0.0604	0.0039	0.8522
Nicolas Batum	G-F	0.2417	0.5991	0.0	0.1591
Norman Powell	G	0.5623	0.0039	0.0	0.4338
OG Anunoby	F	0.1657	0.4025	0.0001	0.4317
Obi Toppin	F	0.0335	0.9665	0.0	0.0001
Otto Porter Jr.	F	0.5854	0.4143	0.0	0.0002
P.J. Dozier	G-F	0.5534	0.2335	0.0002	0.2129
P.J. Tucker	F	0.3482	0.5371	0.0	0.1146

Tab. 14: Non-centers on defense (4).

Name	Label	0 Prob.	1 Prob.	2 Prob.	3 Prob.
Pascal Siakam	F	0.0834	0.9113	0.0	0.0053
Pat Connaughton	G	0.0375	0.9618	0.0	0.0007
Patrick Beverley	G	0.128	0.0068	0.0054	0.8598
Patrick Patterson	F	0.0345	0.9641	0.0	0.0013
Patrick Williams	F	0.3234	0.6725	0.0	0.0041
Patty Mills	G	0.6612	0.0011	0.0	0.3377
Paul George	F	0.3458	0.6472	0.0	0.007
Paul Millsap	F	0.0225	0.9342	0.0	0.0434
Payton Pritchard	G	0.7338	0.1333	0.0001	0.1327
R.J. Hampton	G	0.6023	0.3767	0.0	0.021
RJ Barrett	F-G	0.2865	0.6978	0.0	0.0156
Rajon Rondo	G	0.6297	0.0325	0.0002	0.3375
Raul Neto	G	0.0344	0.0047	0.049	0.9119
Reggie Bullock	G-F	0.5925	0.0515	0.0004	0.3556
Reggie Jackson	G	0.6571	0.3038	0.0	0.0391
Ricky Rubio	G	0.1074	0.0014	0.0025	0.8887
Robert Covington	F	0.0349	0.939	0.0	0.0261
Rodney Hood	G-F	0.9363	0.0509	0.0	0.0127
Royce O'Neale	F	0.0764	0.9229	0.0	0.0007
Rudy Gay	F-G	0.179	0.8188	0.0	0.0022
Rui Hachimura	F	0.2185	0.7813	0.0	0.0003
Russell Westbrook	G	0.7497	0.2502	0.0	0.0001
Saben Lee	G	0.0207	0.0123	0.0418	0.9252
Saddiq Bey	F	0.74	0.2549	0.0	0.0051
Sekou Doumbouya	F	0.1576	0.8419	0.0	0.0005
Semi Ojeleye	F	0.4517	0.5463	0.0	0.002
Seth Curry	G	0.9562	0.003	0.0	0.0408
Shai Gilgeous-Alexander	G-F	0.4935	0.504	0.0	0.0026
Shake Milton	G-F	0.9293	0.0392	0.0	0.0315
Solomon Hill	F	0.3503	0.4456	0.0001	0.204
Stanley Johnson	F-G	0.0221	0.1092	0.0005	0.8682
Stephen Curry	G	0.9892	0.0041	0.0	0.0067
Sterling Brown	G-F	0.0461	0.9482	0.0	0.0057
Svi Mykhailiuk	G-F	0.6023	0.0404	0.0003	0.357

Name	Label	0 Prob.	1 Prob.	2 Prob.	3 Prob.
T.J. McConnell	G	0.0	0.0	0.9999	0.0001
Talen Horton-Tucker	G	0.048	0.082	0.002	0.8681
Taurean Prince	F	0.5538	0.3699	0.0001	0.0763
Terance Mann	G-F	0.1966	0.7771	0.0	0.0263
Terence Davis	G	0.6721	0.0863	0.0003	0.2414
Terrence Ross	G-F	0.7536	0.021	0.0001	0.2253
Terry Rozier	G	0.0836	0.014	0.0115	0.8909
Thaddeus Young	F	0.018	0.9687	0.0	0.0133
Thanasis Antetokounmpo	F	0.0198	0.0693	0.0016	0.9093
Theo Maledon	G	0.8963	0.0724	0.0	0.0313
Tim Hardaway Jr.	G-F	0.8197	0.1651	0.0	0.0153
Timothe Luwawu-Cabarrot	G-F	0.304	0.6643	0.0	0.0317
Tobias Harris	F	0.1042	0.8958	0.0	0.0
Tomas Satoransky	G	0.5889	0.0539	0.0004	0.3568
Tony Snell	G	0.5552	0.4397	0.0	0.0051
Torrey Craig	F	0.0456	0.8886	0.0	0.0659
Trae Young	G	0.9888	0.0003	0.0	0.0109
Trevor Ariza	F	0.0244	0.178	0.0001	0.7975
Trey Burke	G	0.5238	0.0018	0.0	0.4744
Troy Brown Jr.	G-F	0.2473	0.7326	0.0	0.02
Ty Jerome	G-F	0.9569	0.0181	0.0	0.025
Tyler Herro	G	0.408	0.5548	0.0	0.0372
Tyler Johnson	G	0.7192	0.1443	0.0001	0.1363
Tyrese Haliburton	G	0.3525	0.005	0.0003	0.6423
Tyrese Maxey	G	0.6801	0.0629	0.0002	0.2568
Tyus Jones	G	0.2638	0.0047	0.0007	0.7308
Victor Oladipo	G	0.0203	0.0337	0.0086	0.9374
Wayne Ellington	G	0.9722	0.0139	0.0	0.0139
Wes Iwundu	F	0.407	0.4503	0.0001	0.1425
Wesley Matthews	G	0.1358	0.0139	0.006	0.8443
Will Barton	G	0.7728	0.2094	0.0	0.0177
Zach LaVine	G-F	0.6864	0.3112	0.0	0.0024
Zion Williamson	F	0.367	0.557	0.0	0.0759

Part F

Tab. 15: Replacement centers on offense.

Name	Label	Center Prob.
Alen Smailagic	F	0.5199
Alize Johnson	F	0.8368
Amida Brimah	C	0.9305
Anzejs Pasecniks	C-F	0.8642
Boban Marjanovic	C	0.9999
Bruno Fernando	F-C	0.9887
Chris Silva	F	0.7219
Cristiano Felicio	F-C	0.851
Daniel Oturu	C	0.9951
Devontae Cacok	F	0.7649
Dewayne Dedmon	C	0.9979
Donta Hall	C	0.9974
Ed Davis	C-F	0.9872
Freddie Gillespie	F	0.9774
Harry Giles III	F-C	0.8783
JaKarr Sampson	F	0.641
Jahlil Okafor	C-F	0.9856
Jalen Smith	F-C	0.8459
Jontay Porter	C-F	0.7384
Justin Patton	C	0.6701
Juwan Morgan	F	0.8614
Mamadi Diakite	F	0.8934
Marques Bolden	C	0.9272
Nathan Knight	F-C	0.5402
Nick Richards	C	0.9371
Norvel Pelle	C	0.9007
Paul Reed	F	0.6969
Reggie Perry	F-C	0.9482
Rondae Hollis-Jefferson	F	0.6854
Tacko Fall	C	0.9901
Thomas Bryant	C-F	0.7566
Thon Maker	F-C	0.7815
Tyler Cook	F	0.9487
Udoka Azubuike	C-F	0.9139
Vernon Carey Jr.	F-C	0.6026
Vincent Poirier	C-F	0.8907
Will Magnay	C	0.9139

Tab. 16: Replacement centers on defense.

Name	Label	Center Prob.
Alen Smailagic	F	0.5279
Alize Johnson	F	0.6414
Amida Brimah	C	0.9701
Anzejs Pasecniks	C-F	0.996
Boban Marjanovic	C	1.0
Bruno Fernando	F-C	0.996
Cameron Oliver	F	0.5279
Chris Silva	F	0.5458
Cristiano Felicio	F-C	0.8625
D.J. Wilson	F	0.9998
Devontae Cacok	F	0.5637
Dewayne Dedmon	C	0.994
Donta Hall	C	0.99
Ed Davis	C-F	1.0
Ersan Ilyasova	F	0.6713
Freddie Gillespie	F	0.9811
Harry Giles III	F-C	0.9999
Henry Ellenson	F-C	0.8705
JaKarr Sampson	F	1.0
Jabari Parker	F	0.7629
Jahlil Okafor	C-F	1.0
Jalen Smith	F-C	0.9223
Jaren Jackson Jr.	F-C	0.9996
Jontay Porter	C-F	0.99
Justin Patton	C	0.9951
Luka Samanic	F	0.7003
Luke Kornet	F-C	1.0
Mamadi Diakite	F	0.7112
Marques Bolden	C	0.8845
Meyers Leonard	F-C	0.8645
Mfiondu Kabengele	F-C	0.9993
Nathan Knight	F-C	0.9457
Nick Richards	C	0.9661
Norvel Pelle	C	0.9522
Reggie Perry	F-C	0.9821
Tacko Fall	C	0.9841
Thomas Bryant	C-F	1.0
Thon Maker	F-C	0.8924
Trey Lyles	F	0.9119
Udoka Azubuike	C-F	0.996
Vernon Carey Jr.	F-C	0.9183
Vincent Poirier	C-F	0.996
Will Magnay	C	0.9661

Tab. 17: Replacement non-centers on offense (1).

Name	Label	0 Prob.	1 Prob.	2 Prob.	3 Prob.	4 Prob.
Abdel Nader	F	0.338	0.0795	0.0706	0.506	0.006
Adam Mokoka	G	0.5359	0.008	0.3038	0.1514	0.001
Al-Farouq Aminu	F	0.008	0.6779	0.0129	0.2982	0.003
Alfonzo McKinnie	F	0.0269	0.1414	0.0697	0.752	0.01
Amir Coffey	G-F	0.7237	0.0089	0.0636	0.2028	0.001
Anderson Varejao	F	0.0567	0.3751	0.0945	0.4527	0.0209
Andre Roberson	G-F	0.2587	0.1642	0.197	0.3791	0.001
Anthony Gill	F	0.0328	0.2068	0.0567	0.6918	0.0119
Anthony Lamb	F	0.007	0.0358	0.0507	0.9035	0.003
Anthony Tolliver	F	0.1127	0.2542	0.0847	0.5304	0.0179
Ashton Hagans	G-F	0.3019	0.0447	0.2244	0.428	0.001
Axel Toupane	G-F	0.2584	0.0398	0.2018	0.499	0.001
Bol Bol	C-F	0.1373	0.6169	0.0189	0.2259	0.001
Brian Bowen II	F-G	0.1095	0.0438	0.2726	0.5731	0.001
Brodric Thomas	G	0.1781	0.002	0.4438	0.3751	0.001
Bruno Caboclo	F	0.0557	0.3771	0.0945	0.4517	0.0209
CJ Elleby	F-G	0.0538	0.0259	0.1954	0.7238	0.001
Cameron Oliver	F	0.0498	0.3672	0.0836	0.4816	0.0179
Cam Reynolds	F	0.0557	0.2945	0.0975	0.5313	0.0209
Carsen Edwards	G	0.6458	0.004	0.1522	0.197	0.001
Cassius Stanley	G	0.4269	0.007	0.3095	0.2557	0.001
Cassius Winston	G	0.5298	0.007	0.3191	0.1431	0.001
Chandler Hutchison	F-G	0.0229	0.011	0.1086	0.8566	0.001
Charlie Brown Jr.	G	0.503	0.006	0.243	0.247	0.001
Chris Chiozza	G	0.6109	0.005	0.3254	0.0577	0.001
D.J. Wilson	F	0.006	0.7582	0.009	0.2239	0.003
Dakota Mathias	G	0.606	0.006	0.2667	0.1204	0.001
Dante Exum	G	0.4245	0.007	0.327	0.2406	0.001
Darius Miller	F	0.2607	0.1841	0.0726	0.4697	0.0129
Deividas Sirvydis	F-G	0.1968	0.0328	0.2038	0.5656	0.001
Dennis Smith Jr.	G	0.7781	0.002	0.202	0.0169	0.001
Devin Cannady	G	0.498	0.007	0.2908	0.2032	0.001
Devon Dotson	G	0.498	0.008	0.3926	0.1004	0.001
Didi Louzada	G	0.4831	0.008	0.326	0.1819	0.001
E'Twaun Moore	G	0.4821	0.002	0.4542	0.0608	0.001

Name	Label	0 Prob.	1 Prob.	2 Prob.	3 Prob.	4 Prob.
Elijah Bryant	G	0.5522	0.008	0.3244	0.1144	0.001
Elijah Hughes	G	0.5259	0.007	0.3207	0.1454	0.001
Ersan Ilyasova	F	0.0428	0.2261	0.0857	0.6295	0.0159
Frank Mason III	G	0.4657	0.007	0.4318	0.0945	0.001
Frank Ntilikina	G	0.6199	0.004	0.1303	0.2448	0.001
Gabriel Deck	F	0.0338	0.5224	0.0557	0.3751	0.0129
Gary Payton II	G	0.4841	0.0089	0.3141	0.1918	0.001
Glenn Robinson III	F	0.1046	0.0677	0.0608	0.762	0.005
Grant Riller	G	0.5194	0.008	0.3652	0.1065	0.001
Greg Whittington	F	0.0746	0.3144	0.0995	0.4896	0.0219
Henry Ellenson	F-C	0.001	0.7781	0.008	0.2119	0.001
Ignas Brazdeikis	F	0.0537	0.1542	0.2318	0.5493	0.0109
Iman Shumpert	G	0.5339	0.008	0.3367	0.1205	0.001
Isaac Bonga	G	0.0338	0.001	0.0656	0.8986	0.001
Isaiah Joe	G	0.3632	0.002	0.0746	0.5592	0.001
Isaiah Thomas	G	0.4776	0.1075	0.3124	0.1015	0.001
Jabari Parker	F	0.0398	0.5572	0.0677	0.3204	0.0149
Jahmi'us Ramsey	G	0.5755	0.007	0.2763	0.1402	0.001
Jalen Harris	G	0.6836	0.006	0.2308	0.0786	0.001
Jalen Lecque	G	0.5194	0.008	0.3622	0.1095	0.001
James Nunnally	F	0.0568	0.2898	0.0966	0.5369	0.0199
Jared Dudley	F	0.0507	0.2684	0.0895	0.5726	0.0189
Jared Harper	G	0.5483	0.008	0.3343	0.1085	0.001
Jaren Jackson Jr.	F-C	0.001	0.9204	0.004	0.0736	0.001
Jarrell Brantley	F	0.0428	0.2271	0.1125	0.6016	0.0159
Javonte Green	G-F	0.001	0.003	0.0189	0.9761	0.001
Jay Scrubb	G	0.4378	0.007	0.2876	0.2667	0.001
Jaylen Adams	G	0.5502	0.008	0.3323	0.1085	0.001
Jaylen Hoard	F	0.0189	0.7602	0.0318	0.1811	0.008
Jeremiah Martin	G	0.591	0.007	0.3055	0.0955	0.001
Jerome Robinson	G	0.79	0.004	0.1433	0.0617	0.001
Jordan Bell	F	0.0507	0.4328	0.0856	0.4119	0.0189
Jordan Bone	G	0.6833	0.005	0.2131	0.0976	0.001
Jordan Nwora	F	0.0328	0.1282	0.1491	0.6799	0.0099
Josh Green	G	0.0378	0.003	0.0607	0.8975	0.001

Tab. 18: Replacement non-centers on offense (2).

Name	Label	0 Prob.	1 Prob.	2 Prob.	3 Prob.	4 Prob.
Josh Hall	F	0.1413	0.0866	0.1881	0.5771	0.007
Justin James	G-F	0.1104	0.0149	0.2498	0.6239	0.001
Justin Robinson	G	0.5637	0.007	0.2839	0.1444	0.001
KZ Okpala	F-G	0.009	0.005	0.0657	0.9194	0.001
Karim Mane	G	0.5244	0.007	0.2856	0.1821	0.001
Keita Bates-Diop	F	0.0438	0.1534	0.0807	0.7112	0.011
Kelan Martin	F	0.0836	0.1025	0.0816	0.7244	0.008
Keljin Blevins	G	0.4418	0.007	0.2935	0.2567	0.001
Kevin Knox II	F	0.005	0.0129	0.0617	0.9184	0.002
Khyri Thomas	G	0.3552	0.0796	0.3532	0.2109	0.001
Killian Tillie	F-C	0.001	0.5194	0.0239	0.4547	0.001
Kostas Antetokounmpo	F	0.0527	0.3562	0.0896	0.4816	0.0199
Kris Dunn	G	0.5706	0.008	0.3171	0.1034	0.001
Kyle Guy	G	0.6402	0.005	0.2962	0.0577	0.001
Langston Galloway	G	0.5902	0.001	0.0339	0.3739	0.001
Louis King	F	0.0498	0.2647	0.1075	0.5592	0.0189
Luka Samanic	F	0.0219	0.1164	0.0478	0.805	0.009
Luke Kornet	F-C	0.001	0.9184	0.003	0.0766	0.001
Malik Fitts	F	0.0627	0.3154	0.1015	0.4985	0.0219
Markelle Fultz	G	0.503	0.005	0.4303	0.0608	0.001
Markus Howard	G	0.7012	0.005	0.2271	0.0657	0.001
Marquese Chriss	F	0.0577	0.3632	0.0965	0.4617	0.0209
Mason Jones	G	0.7278	0.003	0.2373	0.0309	0.001
Matt Thomas	G	0.79	0.003	0.1244	0.0816	0.001
Matthew Dellavedova	G	0.6328	0.005	0.3005	0.0607	0.001
Meyers Leonard	F-C	0.001	0.798	0.008	0.192	0.001
Mfiondu Kabengele	F-C	0.001	0.9114	0.003	0.0836	0.001
Mike James	G	0.5234	0.005	0.4129	0.0577	0.001
Nate Darling	G	0.5388	0.008	0.327	0.1252	0.001
Nate Hinton	G-F	0.4289	0.0358	0.1871	0.3473	0.001
Nico Mannion	G	0.7602	0.003	0.21	0.0259	0.001
Noah Vonleh	F	0.0597	0.3383	0.1005	0.4796	0.0219
Patrick McCaw	G	0.51	0.008	0.3247	0.1564	0.001
Paul Watson	G	0.4866	0.004	0.1323	0.3761	0.001
Quinn Cook	G	0.668	0.006	0.2495	0.0755	0.001

Name	Label	0 Prob.	1 Prob.	2 Prob.	3 Prob.	4 Prob.
Quinndary Weatherspoon	G	0.4	0.007	0.4527	0.1393	0.001
Ray Spalding	F	0.0586	0.3519	0.0984	0.4692	0.0219
Rayjon Tucker	G	0.4771	0.007	0.4163	0.0986	0.001
Robert Franks	F	0.0478	0.2537	0.0866	0.594	0.0179
Robert Woodard II	F	0.0548	0.2888	0.0966	0.5398	0.0199
Rodions Kurucs	F	0.0438	0.2321	0.0876	0.6205	0.0159
Rodney McGruder	G	0.3303	0.006	0.2806	0.3821	0.001
Romeo Langford	G-F	0.1224	0.0189	0.1214	0.7363	0.001
Ryan Arcidiacono	G	0.8884	0.001	0.0508	0.0588	0.001
Sam Merrill	G	0.6935	0.005	0.1811	0.1194	0.001
Sean McDermott	F	0.1662	0.2129	0.0736	0.5323	0.0149
Shaquille Harrison	G	0.6905	0.003	0.2438	0.0617	0.001
Sindarius Thornwell	G	0.3085	0.005	0.2129	0.4726	0.001
Skylar Mays	G	0.2886	0.004	0.6488	0.0577	0.001
Spencer Dinwiddie	G	0.4587	0.007	0.4169	0.1164	0.001
T.J. Leaf	F	0.0557	0.2955	0.0985	0.5294	0.0209
T.J. Warren	F	0.0458	0.247	0.0886	0.6016	0.0169
Terrance Ferguson	G	0.5194	0.008	0.3085	0.1632	0.001
Theo Pinson	G-F	0.2816	0.0418	0.208	0.4677	0.001
Tim Frazier	G	0.5727	0.007	0.3217	0.0976	0.001
Tre Jones	G	0.3755	0.004	0.5697	0.0498	0.001
Tremont Waters	G	0.6213	0.005	0.3171	0.0557	0.001
Trent Forrest	G	0.7177	0.004	0.2336	0.0437	0.001
Trey Lyles	F	0.0845	0.0835	0.0666	0.7584	0.007
Ty-Shon Alexander	G	0.5234	0.008	0.3323	0.1353	0.001
Tyler Bey	F	0.0517	0.2704	0.0895	0.5696	0.0189
Tyrell Terry	G	0.5721	0.008	0.3194	0.0995	0.001
Udonis Haslem	F	0.0607	0.3274	0.1015	0.4886	0.0219
Vlatko Cancar	F	0.0239	0.1243	0.0517	0.7913	0.0089
Wenyen Gabriel	F	0.0299	0.194	0.0527	0.7124	0.0109
Yogi Ferrell	G	0.3831	0.007	0.5284	0.0806	0.001
Zeke Nnaji	F-C	0.001	0.1352	0.0318	0.831	0.001

Tab. 19: Replacement non-centers on defense (1).

Name	Label	0 Prob.	1 Prob.	2 Prob.	3 Prob.
Abdel Nader	F	0.2566	0.6802	0.0008	0.0623
Adam Mokoka	G	0.5233	0.1013	0.0457	0.3297
Al-Farouq Aminu	F	0.1827	0.7276	0.0008	0.0889
Alfonzo McKinnie	F	0.2782	0.6528	0.0008	0.0681
Amir Coffey	G-F	0.7907	0.0997	0.01	0.0997
Anderson Varejao	F	0.2135	0.6794	0.0017	0.1055
Andre Roberson	G-F	0.3807	0.3408	0.0258	0.2527
Anthony Gill	F	0.1784	0.7386	0.0017	0.0813
Anthony Lamb	F	0.0989	0.8712	0.0008	0.0291
Anthony Tolliver	F	0.2195	0.5993	0.0017	0.1796
Ashton Hagans	G-F	0.4302	0.2608	0.0282	0.2807
Axel Toupane	G-F	0.3779	0.2334	0.137	0.2517
Bol Bol	C-F	0.0183	0.9801	0.0008	0.0008
Brian Bowen II	F-G	0.2664	0.5062	0.0066	0.2207
Brodric Thomas	G	0.1754	0.4871	0.0158	0.3217
Bruno Caboclo	F	0.2135	0.6802	0.0017	0.1047
CJ Elleby	F-G	0.4423	0.3826	0.005	0.1701
Cam Reynolds	F	0.2118	0.6827	0.0017	0.1038
Carsen Edwards	G	0.4385	0.0606	0.0266	0.4743
Cassius Stanley	G	0.4448	0.2108	0.0423	0.3021
Cassius Winston	G	0.5507	0.1071	0.0424	0.2998
Chandler Hutchison	F-G	0.1404	0.7741	0.0033	0.0822
Charlie Brown Jr.	G	0.4534	0.1131	0.0366	0.3968
Chris Chiozza	G	0.3679	0.0764	0.0316	0.5241
Dakota Mathias	G	0.3776	0.2996	0.0398	0.283
Daniel Oturu	C	0.0889	0.858	0.0008	0.0523
Dante Exum	G	0.478	0.1842	0.0407	0.2971
Darius Miller	F	0.3104	0.4299	0.0017	0.2581
Deividas Sirvydis	F-G	0.2193	0.6013	0.0058	0.1736
Dennis Smith Jr.	G	0.1545	0.3081	0.015	0.5224
Devin Cannady	G	0.4133	0.0996	0.0573	0.4299
Devon Dotson	G	0.4294	0.103	0.1395	0.3281
Didi Louzada	G	0.451	0.1096	0.0457	0.3937
E'Twaun Moore	G	0.2932	0.088	0.0183	0.6005
Elijah Bryant	G	0.446	0.1661	0.0473	0.3405
Elijah Hughes	G	0.4797	0.156	0.0448	0.3195
Frank Mason III	G	0.5174	0.1246	0.044	0.314
Frank Ntilikina	G	0.2176	0.0523	0.2558	0.4743
Gabriel Deck	F	0.3892	0.5378	0.0017	0.0714
Gary Payton II	G	0.4377	0.1047	0.1229	0.3347
Glenn Robinson III	F	0.211	0.7467	0.0008	0.0415
Grant Riller	G	0.4547	0.1081	0.049	0.3882
Greg Whittington	F	0.245	0.6437	0.0017	0.1096
Ignas Brazdeikis	F	0.25	0.6852	0.0008	0.064
Iman Shumpert	G	0.4643	0.1113	0.0673	0.3571
Isaac Bonga	G	0.2542	0.4277	0.0116	0.3065
Isaiah Joe	G	0.217	0.0441	0.0233	0.7157
Isaiah Thomas	G	0.5195	0.1037	0.0465	0.3303
Jahmi'us Ramsey	G	0.4938	0.1602	0.0415	0.3046
Jalen Harris	G	0.3596	0.0831	0.0382	0.5191
Jalen Lecque	G	0.4635	0.1329	0.0498	0.3538
James Nunnally	F	0.2929	0.6025	0.0017	0.1029
Jared Dudley	F	0.1978	0.7007	0.0017	0.0998
Jared Harper	G	0.4826	0.1188	0.049	0.3497
Jarrell Brantley	F	0.2149	0.6979	0.0017	0.0855
Javonte Green	G-F	0.0888	0.0473	0.1037	0.7602
Jay Scrubb	G	0.5087	0.0964	0.0432	0.3516
Jaylen Adams	G	0.4676	0.1337	0.049	0.3497
Jaylen Hoard	F	0.3577	0.5416	0.0008	0.0998
Jeremiah Martin	G	0.412	0.1047	0.0449	0.4385
Jerome Robinson	G	0.235	0.0756	0.0266	0.6628
Jordan Bell	F	0.1968	0.7051	0.0017	0.0963
Jordan Bone	G	0.6528	0.0756	0.0332	0.2384
Jordan Nwora	F	0.2377	0.5569	0.0008	0.2045
Josh Green	G	0.2076	0.0299	0.015	0.7475
Josh Hall	F	0.6071	0.348	0.0008	0.044

Tab. 20: Replacement non-centers on defense (2).

Name	Label	0 Prob.	1 Prob.	2 Prob.	3 Prob.
Justin James	G-F	0.3544	0.4523	0.0133	0.1801
Justin Robinson	G	0.4958	0.0963	0.0432	0.3646
Juwan Morgan	F	0.1711	0.745	0.0017	0.0822
KZ Okpala	F-G	0.0739	0.7841	0.0025	0.1395
Karim Mane	G	0.4141	0.2357	0.0432	0.3071
Keita Bates-Diop	F	0.2121	0.5732	0.0008	0.2138
Kelan Martin	F	0.1304	0.8198	0.0008	0.049
Keljin Blevins	G	0.4746	0.1189	0.0432	0.3633
Kevin Knox II	F	0.6055	0.3422	0.0008	0.0515
Khyri Thomas	G	0.3787	0.093	0.0382	0.49
Killian Tillie	F-C	0.0947	0.8912	0.0008	0.0133
Kostas Antetokounmpo	F	0.2041	0.6564	0.0149	0.1245
Kris Dunn	G	0.436	0.1088	0.0465	0.4086
Kyle Guy	G	0.6373	0.1253	0.029	0.2083
Langston Galloway	G	0.8022	0.1031	0.0116	0.0831
Louis King	F	0.1934	0.556	0.1494	0.1012
Malik Fitts	F	0.2243	0.6636	0.0017	0.1105
Markelle Fultz	G	0.5112	0.079	0.0316	0.3782
Markus Howard	G	0.6199	0.1228	0.0315	0.2257
Marquese Chriss	F	0.2168	0.6752	0.0017	0.1063
Mason Jones	G	0.6384	0.1995	0.02	0.1421
Matt Thomas	G	0.5723	0.2517	0.0216	0.1545
Matthew Dellavedova	G	0.6409	0.0998	0.0316	0.2278
Mike James	G	0.6376	0.1131	0.0299	0.2195
Nate Darling	G	0.4576	0.1105	0.0482	0.3837
Nate Hinton	G-F	0.4734	0.2218	0.0241	0.2807
Nico Mannion	G	0.2841	0.0482	0.0208	0.647
Noah Vonleh	F	0.2243	0.6636	0.0017	0.1105
Patrick McCaw	G	0.4515	0.1079	0.049	0.3917
Paul Reed	F	0.1578	0.5947	0.0017	0.2458
Paul Watson	G	0.2882	0.2051	0.0241	0.4826
Quinn Cook	G	0.5718	0.0954	0.0365	0.2963
Quinndary Weatherspoon	G	0.3713	0.0889	0.2566	0.2832

Name	Label	0 Prob.	1 Prob.	2 Prob.	3 Prob.
Ray Spalding	F	0.2201	0.6703	0.0017	0.108
Rayjon Tucker	G	0.4136	0.0988	0.1611	0.3264
Robert Franks	F	0.2434	0.6512	0.0017	0.1038
Robert Woodard II	F	0.2085	0.6877	0.0017	0.1022
Rodions Kurucs	F	0.1909	0.7095	0.0017	0.0979
Rodney McGruder	G	0.3367	0.079	0.0416	0.5428
Romeo Langford	G-F	0.3643	0.1859	0.0158	0.434
Rondae Hollis-Jefferson	F	0.1993	0.7076	0.0017	0.0914
Ryan Arcidiacono	G	0.4639	0.0581	0.0124	0.4656
Sam Merrill	G	0.6218	0.1297	0.0291	0.2195
Sean McDermott	F	0.2633	0.6487	0.0017	0.0864
Shaquille Harrison	G	0.2292	0.064	0.0266	0.6802
Sindarius Thornwell	G	0.358	0.0806	0.0332	0.5282
Skylar Mays	G	0.2791	0.0631	0.0382	0.6196
Spencer Dinwiddie	G	0.4373	0.1983	0.0448	0.3195
T.J. Leaf	F	0.2133	0.6805	0.0017	0.1046
T.J. Warren	F	0.3776	0.522	0.0017	0.0988
Terrance Ferguson	G	0.5104	0.1131	0.0466	0.33
Theo Pinson	G-F	0.3987	0.3106	0.0266	0.2641
Tim Frazier	G	0.485	0.1478	0.0449	0.3223
Tre Jones	G	0.5648	0.0648	0.0274	0.343
Tremont Waters	G	0.2988	0.0656	0.0324	0.6033
Trent Forrest	G	0.4767	0.3007	0.0249	0.1977
Ty-Shon Alexander	G	0.4369	0.1836	0.0465	0.3331
Tyler Bey	F	0.1977	0.7035	0.0017	0.0972
Tyler Cook	F	0.3056	0.6653	0.0008	0.0282
Tyrell Terry	G	0.4261	0.1022	0.142	0.3297
Udonis Haslem	F	0.2274	0.6539	0.0058	0.1129
Vlatko Cancar	F	0.3461	0.5923	0.0008	0.0607
Wenyen Gabriel	F	0.1379	0.7957	0.0008	0.0656
Yogi Ferrell	G	0.5373	0.0871	0.0821	0.2935
Zeke Nnaji	F-C	0.0166	0.9767	0.0008	0.0058
